# Carbon–carbon bond activation of cyclobutenones enabled by the addition of chiral organocatalyst to ketone

**DOI:** 10.1038/ncomms7207

**Published:** 2015-02-05

**Authors:** Bao-Sheng Li, Yuhuang Wang, Zhichao Jin, Pengcheng Zheng, Rakesh Ganguly, Yonggui Robin Chi

**Affiliations:** 1 Nanyang Technological University, Division of Chemistry & Biological Chemistry, School of Physical & Mathematical Sciences, Singapore, 637371, Singapore; 2 Laboratory Breeding Base of Green Pesticide and Agricultural Bioengineering, Key Laboratory of Green Pesticide and Agricultural Bioengineering, Ministry of Education, Guizhou University, Huaxi District, Guiyang 550025, China

## Abstract

The activation of carbon–carbon (C–C) bonds is an effective strategy in building functional molecules. The C–C bond activation is typically accomplished via metal catalysis, with which high levels of enantioselectivity are difficult to achieve due to high reactivity of metal catalysts and the metal-bound intermediates. It remains largely unexplored to use organocatalysis for C–C bond activation. Here we describe an organocatalytic activation of C–C bonds through the addition of an NHC to a ketone moiety that initiates a C–C single bond cleavage as a key step to generate an NHC-bound intermediate for chemo- and stereo-selective reactions. This reaction constitutes an asymmetric functionalization of cyclobutenones using organocatalysts via a C–C bond activation process. Structurally diverse and multicyclic compounds could be obtained with high optical purities via an atom and redox economic process.

## Abstract (web summary)

Activation of C–C bonds is a powerful method for directly functionalizing organic molecules, but is still a challenging task. Here, the authors show that *N*-heterocyclic carbenes can activate the C–C bonds in cyclic ketones, allowing the enantioselective formation of lactams.

## Graphical abstract

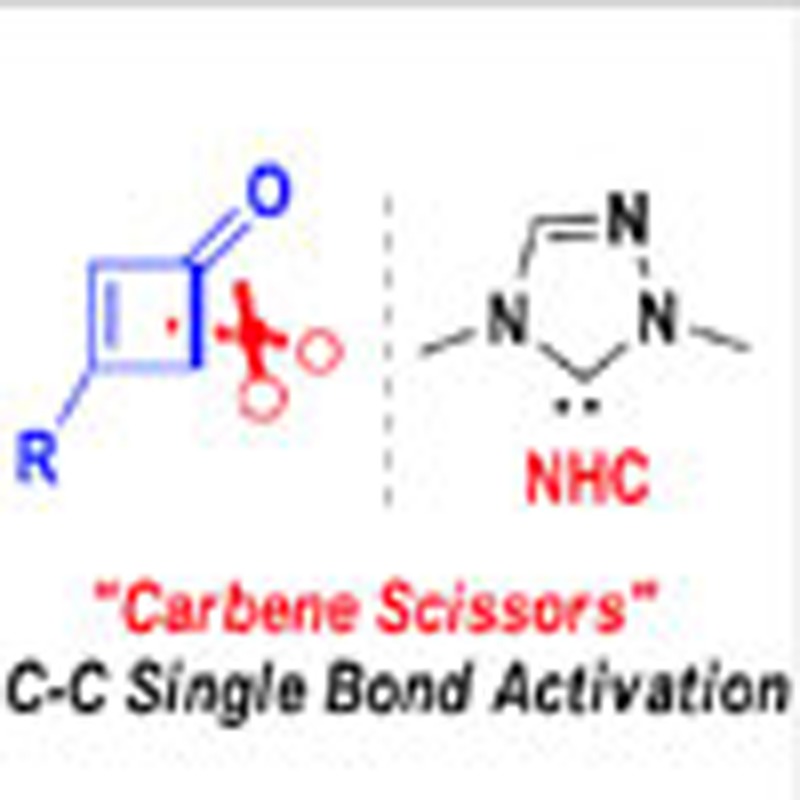

The catalytic activation of a carbon–carbon (C–C) single bond of cyclobutenones can provide direct methods towards building useful molecules^1^,^2^,^3^,^4^,^5^. Despite the rather clear practical significance, C–C bond activation remains challenging. Traditionally, this process is initiated by the oxidative addition of a transition metal catalyst to the C–C bond followed by other bond breaking and formation events (Fig. 1a). Due to the high reactivities of the metal catalyst and the metal-bound intermediates, chemoselectivity is generally difficult to control. In addition, it still remains difficult to achieve high levels of enantioselectivity using the transition metal-catalysed C–C bond activation approach^6^,^7^,^8^,^9^,^10^,^11^. In many cases, intramolecular reactions were used to overcome the challenging selectivity issues.

Our laboratory is interested in developing organocatalysis for challenging bond activations while maintaining the power of organocatalysis for chemo- and stereo-selectivity control. Herein, we report the addition of an *N*-heterocyclic carbene (NHC) organocatalyst to a ketone moiety that initiates a C–C single bond cleavage to generate an NHC-bound intermediate for chemo- and stereo-selective reactions (Fig. 1b). Compared with the earlier NHC catalysis (such as oxidative NHC catalysis for γ-carbon functionalization of enals)^12^,^13^,^14^,^15^,^16^,^17^,^18^, in this approach all atoms of the substrate end up in the product (atom economy) and the overall reaction is redox-neutral (redox economy)^19^. Specifically, the addition of an NHC catalyst to an unsaturated four-membered cyclo-ketone substrate to form intermediate **I**. Breaking a C–C bond of the four-membered ring eventually generates a vinyl enolate intermediate^12^,^13^,^14^,^15^,^16^,^17^,^18^,^19^,^20^,^21^,^22^ **II** that reacts with an imine substrate to form the lactam product. NHC catalysis is routinely used in the activation of aldehydes through the formation of Breslow intermediates^23^,^24^,^25^,^26^,^27^,^28^,^29^,^30^,^31^,^32^. The addition of NHC catalyst to ketone moiety for reactions is much less studied, except for the activation of α-hydroxyl ketones via retro-benzoin pathways as nicely illustrated by Bode and co-workers^33^,^34^.

Our interest in aza-quaternary center compounds^35^ with important biological activity motivated us to use four-membered cyclo-ketone substrate (**1a**) and imine (**2a**) as model substrates for the search of suitable catalytic conditions (Table 1). As an important note, although four-membered cyclo-ketones were nearly untouched in organocatalysis, this class of molecules caught considerable attentions in the field of transition metal catalysis (Fig. 1a). Murakami *et al*.^36^,^37^ have pioneered the non-enantioselective C–C bond activation of four-membered cyclic ketones to react with olefins in an intramolecular fashion^38^,^39^,^40^,^41^,^42^. Recently, impressive enantioselective intramolecular reactions enabled by the metal-catalysed C–C bond activation of four-membered cyclo-ketones were reported by the groups of Dong^10^ and Cramer^11^. The related cyclobutanol has also been used in the synthesis via C–C bond breaking to build sophisticated molecules, as illustrated by Trost,^43^ Tu^44^,^45^ and others^43^,^44^,^45^,^46^,^47^.

## Results

### Reaction optimization

As briefed in Table 1, triazolium NHCs (**A**, **B**, entries 1 and 2) could smoothly mediate the formation of desired product **3a** as essentially a single diastereomer. The *N*-aryl substituent (phenyl or mesityl) of pre-catalyst **A**^48^ and **B**^49^,^50^ had little effect on the reaction yield. Next the enantioselectivity of this transformation was evaluated with aminoindanol-derived triazolium salts **C**–**F**^49^,^50^,^51^,^52^ (entries 3–6). In all cases, the product **3a** was formed essentially as a single diastereomer with good yields (entries 1–6). Among precatalysts **C**–**F**, the *N*-aryl substituents could affect the reaction enantioselectivities (entries 3–6). The use of *N*-mesityl substituted triazolium catalyst **D**^48^ gave the product **3a** with the highest enantioselectivity (90:10 er) and good yield (84%, entry 4). We then noticed that increasing the reaction temperature to 55 °C could reduce the reaction time from 48 to 24 h and there was a small but reproducible increase of er without sacrificing the yield or er (entry 7) for unclear reasons. Interestingly, there was a small but reproducible increase of er when increasing the reaction temperature from room temperature (90:10 er, entry 4) to 55 °C (92:8 er, entry 7).

### Substrates scope with sulfonyl imines

**Figure 2:**
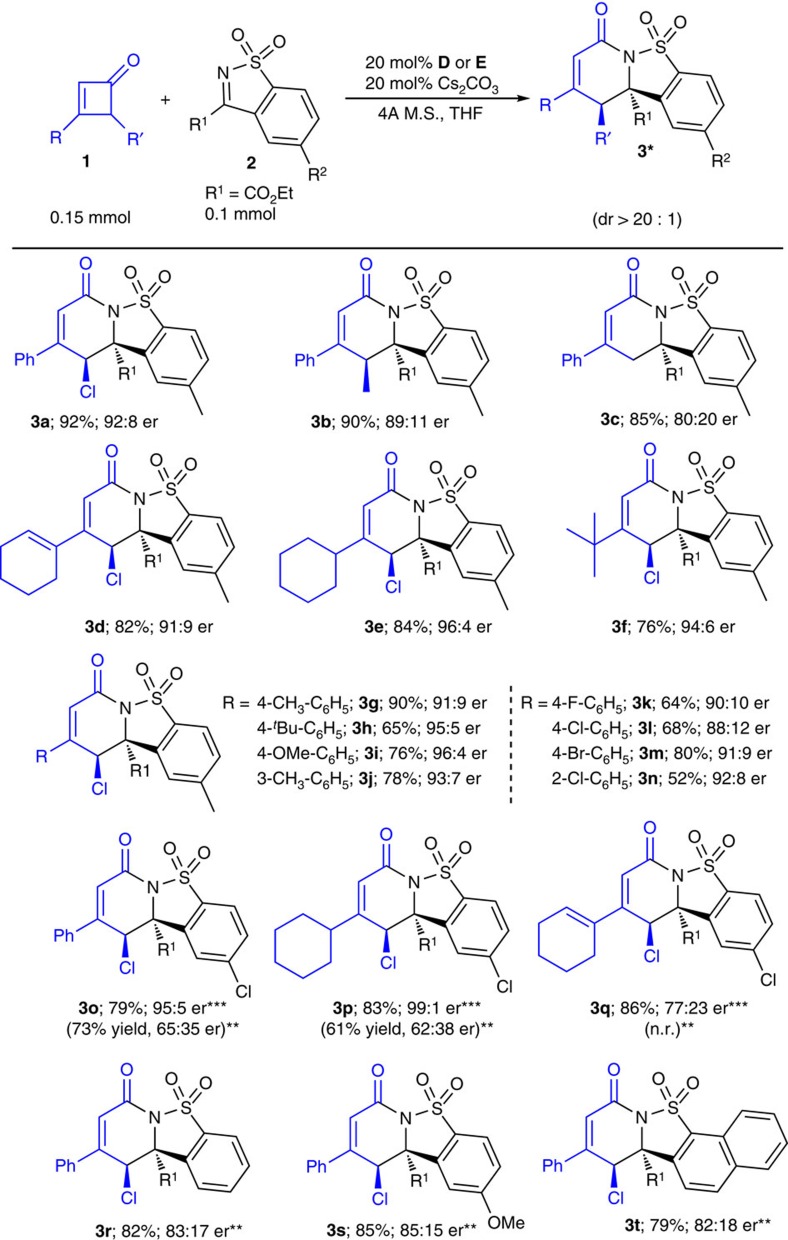
Reaction scope. *The scope of this catalytic transformation was evaluated under standard conditions (Table 1, entry 7). Substrate scope includes γ- (**3a**–**3c**) and β-substituents (**3d**–**3n**) cyclobutenones (using **2a** as the optimal imine), and various imines (**3o**–**3t**, using **1a** as the optimal substrate). Reported yields were isolated yields of **3** based on imine **2**. Diastereoselective ratio (dr of **3** was determined via ^1^H NMR analysis of the unpurified reaction mixture. Relative configuration of the major diastereoisomer was assigned based on X-ray structure of **3b** and **3m** (CCDC 988901, CCDC 988902, see Supplementary Information for more details). **The reactions were performed at 25 °C for 36 h. ***The reactions were performed using pre-catalyst **E** at 25 °C for 36 h (the reaction temperature was 0 °C for **3p**).

With optimized condition (Table 1, entry 7) in hand, we next evaluated the scope of this reaction (Fig. 2). The R′ substituent at the γ-carbon of cyclobutenone **1** could be Cl (**3a)**, methyl (**3b)** or a proton (**3c)**. When the R’ substituent was an aryl unit, the α,β-double bond in the four-membered ring could easily migrate to the β,γ-carbons. Placing a substituent (such as a CH_3_ or Cl unit) at the α-carbon of **1** led to nearly no reaction, and the ketone substrate was recovered under the reaction condition. The β-aryl group of **1** could be replaced with an alkenyl (**3d**), cyclohexyl (**3e**) or a *tert*-butyl (**3f**) substituent. Placing substituents with various electronic properties at the β-aryl group of **1** were all tolerated (**3g**–**n**). When an electron withdrawing group (Cl) was used to replace the methyl group of imine substrate **2** (**3o–3q**), the reaction gave good yields but much lower ers. We then found that by using the more electron-deficient NHC pre-catalyst **E**, the products (**3o–3q**) could be obtained with good to excellent enantiomeric excesses. It appears that the electronic properties of both the imine substrates and NHC catalysts could significantly affect the enantioselectivities in our reaction system^53^,^54^,^55^,^56^. For example, with the *N*-mesityl catalyst **D** as the NHC pre-catalyst, the reaction with imine **2a** gave product **3a** with 92:8 er; while under the same conditions the use of imine substrate with a chlorine substituent gave product **3o** with 65:35 er. Similar effects with *N*-trichloro phenyl catalyst **E** were observed. When **E** was the catalyst, **3a** was obtained with 64:36 er (Table 1, entry 5) while **3o** was observed with 95:5 er. The imine substrate bearing no substituent at the phenyl group also reacted well to give **3r** with good yield, albeit with lower er. Results comparable to those of **3r** were observed when imines bearing a methoxylphenyl (**3s**) or naphthyl (**3t**) unit were used.

### Substrates scope with isatin imines

**Figure 3:**
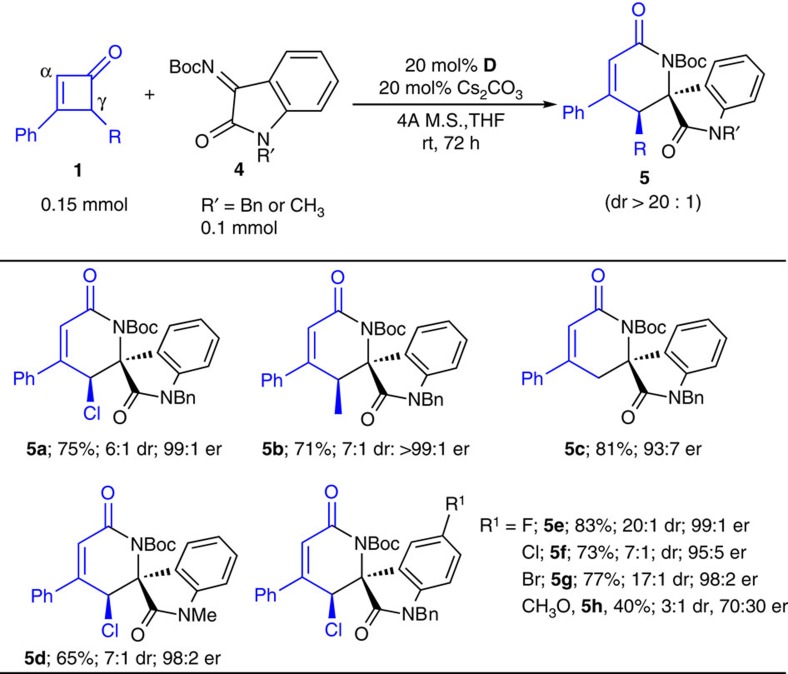
Reaction scope. Substrate scope includes β-phenyl with various γ-substituents (**1a**–**1c**; using **4a** as the optimal imine), and various imines (using **1a** as the optimal substrate). Reported yields were isolated yields of **5** based on imine **4**. Diastereoselective ratio (dr of **5** was determined via ^1^H NMR analysis of the unpurified reaction mixture. Relative configuration of the major diastereoisomer was assigned based on X-ray structure of **5f** and **5g** (CCDC 1011138 and CCDC 1011137, see Supplementary Information for more details). Ers (major diastereomer) were determined via chiral phase high-performance liquid chromatography analysis.

To further explore the scope of the C–C bond activation reaction, we next examined Boc-protected imines derived from isatins to react with cyclobutenones **1** (Fig. 3). Catalyst **D** used in the earlier reactions (Table 1) was found effective here. Optimal results were obtained when the reactions were performed at room temperature for 72 h. The reactions proceeded to give tricyclic molecules (**5a**–**g**) containing spiro quaternary carbon centres with good drs and excellent ers. When substrate with electron-donating substituent (such as a methoxyl group, **5h**) was used, a drop in both the reaction yields and stereo-selectivities were observed.

## Discussion

These two types of reactions summarized in Figs 2 and 3 exemplified the potential value of our C–C activation strategy. The resulting products contains two contiguous stereogenic centres and one aza-quaternary center. Notably, our approach also allows for the construction of chiral carbon stereocenters bearing a Cl atom. The absolute configurations of the reaction products were determined based on their ^1^H nuclear magnetic resonance (^1^H NMR) spectra and X-ray diffraction of the products **3b**, **3m**, **5f** and **5g**.

As a note, installing substituents at the ketone β-aryl group led to a significant drop in the reaction yields with little or no enantioselectivities for the isatin imine reactions, for reasons unclear at this moment (Fig. 3). The use of other imines (such as *N*-tosyl imine derived from benzaldehyde or aryl trifluoroacetone) led to no detecable lactam product. The imine substrates were hydrolysed at an elongated time in our reaction system. A better understanding between the substrate structures and reactivities require further investigations.

In addition, cyclobutenones are known to undergo ring opening to form vinyl ketenes under thermal conditions. However, in our reactions such ring opening was unlikely to occur because: (a) the cyclobutenone substrates were stable (no ring opening) in the absence of carbene catalysts under our reaction condition, (b) our catalytic condition could occur at room or lower temperatures, such as synthesis of the product **3p** in Fig. 2. In our reaction, the addition of carbene catalyst initiated a key C–C breaking step to generate the vinyl enolate intermediate^57^.

In summary, we have demonstrated the use of an NHC catalyst to catalyse the breaking of C–C single bonds for asymmetric reactions^58^,^59^,^60^,^61^,^62^,^63^,^64^,^65^,^66^,^67^. To the best of our knowledge, this is the first example demonstrating asymmetric functionalization of cyclobutenone using organocatalyst. Structurally diverse and multicyclic compounds could be obtained with high optical purities via an atom and redox economical process. Built upon this strained four-membered ring ketone activation, we are looking into other more common ketone compounds. We also expect this study to encourage further investigations into organocatalytic strategies for new C–C activations and highly economical syntheses.

## Methods

### Materials

For ^1^H, ^13^C NMR and high-performance liquid chromatography spectra of compounds in this manuscript, see Supplementary Figs 1–68. For details of the synthetic procedures, see Supplementary Methods.

### Syntheses of products 3 and 5

To a dry Schlenk tube equipped with a magnetic stir bar, were added cyclobuteneones **1** (0.15 mmol), imines **2** or **4** (0.1 mmol), triazolium salt **D** or **E** (0.02 mmol), Cs_2_CO_3_ (0.02 mmol, 6.5 mg) and 4A molecular sieve (100 mg). The tube was closed with a septum, evacuated and refilled with nitrogen. Then, the freshly distilled tetrahydrofuran (1.0 ml) was added. The reaction mixture was stirred at the specified temperature as showed in Fig. 2 and Fig. 3 in the text. After the complete consumption of imines by was monitoring by thin-layer chromatography, the mixture was concentrated under reduced pressure. The resulting crude residue was purified via column chromatography on silica gel to afford the desired products **3** or **5**.

## Author contributions

B.-S.L. conducted most of the experiments; Y.W., Z.J. and P.Z. prepared the substrates and catalysts for the reaction scope evaluation; R.G. contributed to X-ray analysis. Y.R.C. conceptualized and directed the project, and drafted the manuscript with the assistance from all the co-authors. All authors contributed to discussions.

## Additional information

**Accession codes**: For ORTEPs of products **3b**, **3m**, **5f** and **5g**, see Supplementary Information. CCDC 988901, CCDC 988902, CCDC 1011138 and CCDC 1011137 contain supplementary crystallographic data for this paper. These data could be obtained free of charge from The Cambridge Crystallographic Data Centre via www.ccdc.cam.ac.uk/data_request/cif.

**How to cite this article:** Li, B.-S. *et al*. Carbon–carbon bond activation of cyclobutenones enabled by the addition of chiral organocatalyst to ketone. *Nat. Commun.* 6:6207 doi: 10.1038/ncomms7207 (2015).

## Supplementary Material

Supplementary Figures, Supplementary Methods and Supplementary References Supplementary Figures 1-68, Supplementary Methods and Supplementary References

## Figures and Tables

**Figure 1:**
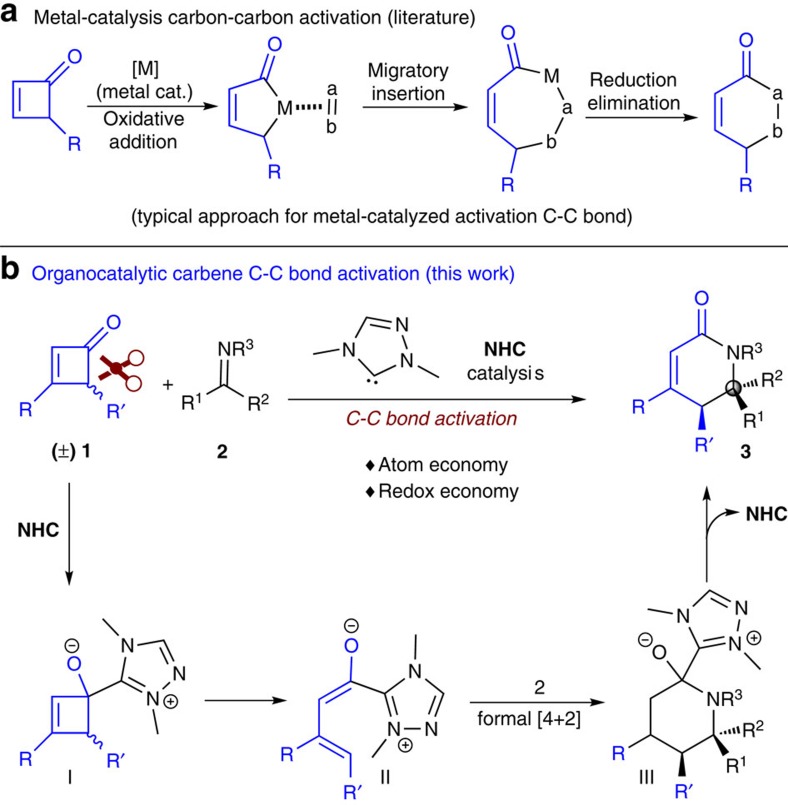
NHC-catalysed cyclization via carbon–carbon bond activation of ketones. (**a**) Metal-catalysed activation of carbon–carbon bond. (**b**) Our synthetic proposal via an organocatalysis. NHCs react with cyclobutenone to generate chiral vinylenolate intermediate to give novel formal cycloaddition reactions.

**Table 1:** Condition optimization.
