# Supplementary material for: Carbon–carbon bond activation of cyclobutenones enabled by the addition of chiral organocatalyst to ketone
Source: Nat Commun. 2015 Feb 5;6:6207. doi: 10.1038/ncomms7207 (PMC4327543; doi:10.1038/ncomms7207)
Supplement: Supplementary Figures, Supplementary Methods and Supplementary References — Supplementary Figures 1-68, Supplementary Methods and Supplementary References [file ncomms7207-s1.pdf]

Supplementary Figures

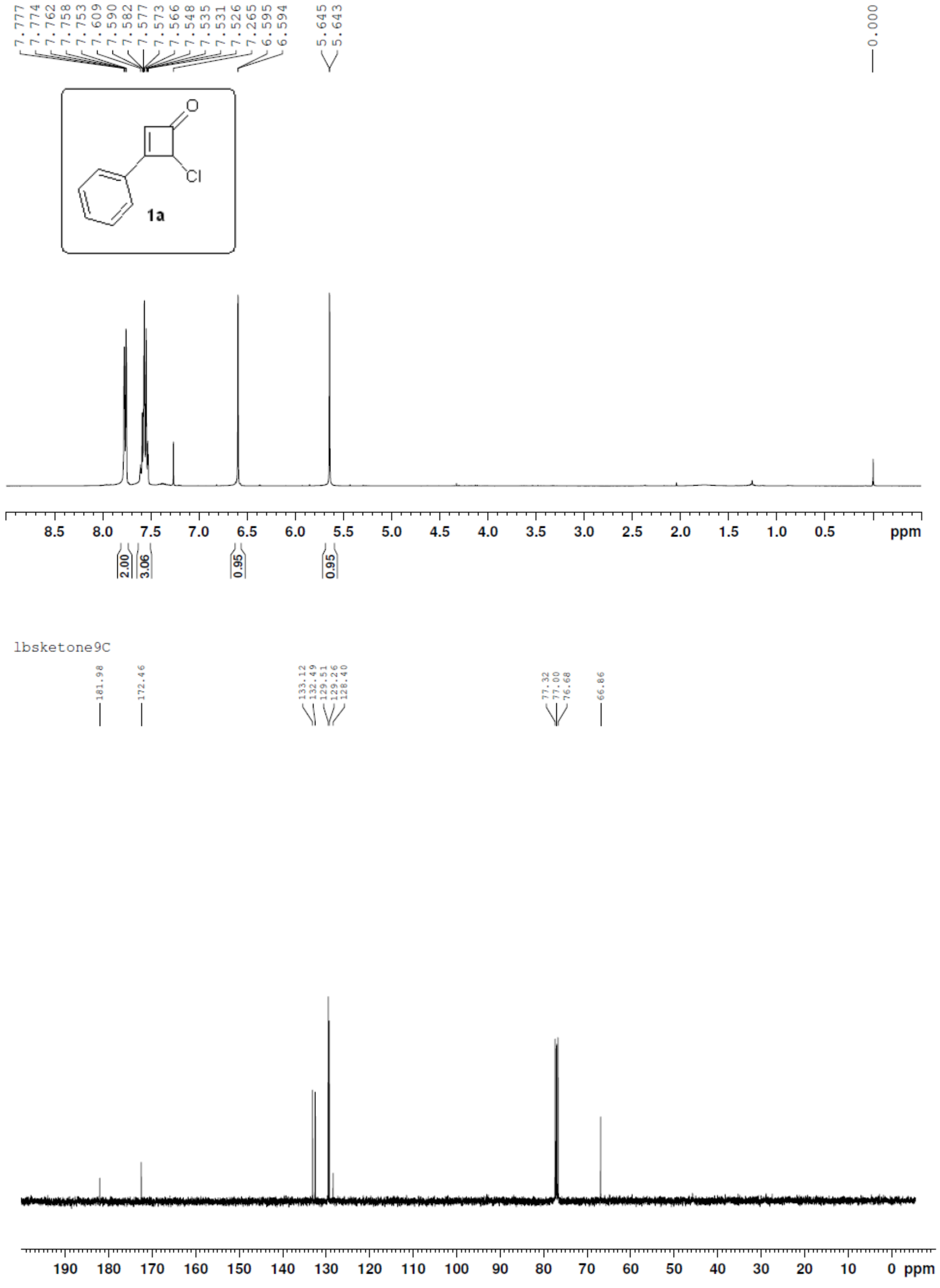

Supplementary figure 1. ^{1}H and ^{13}C NMR spectra for substrate **1a**

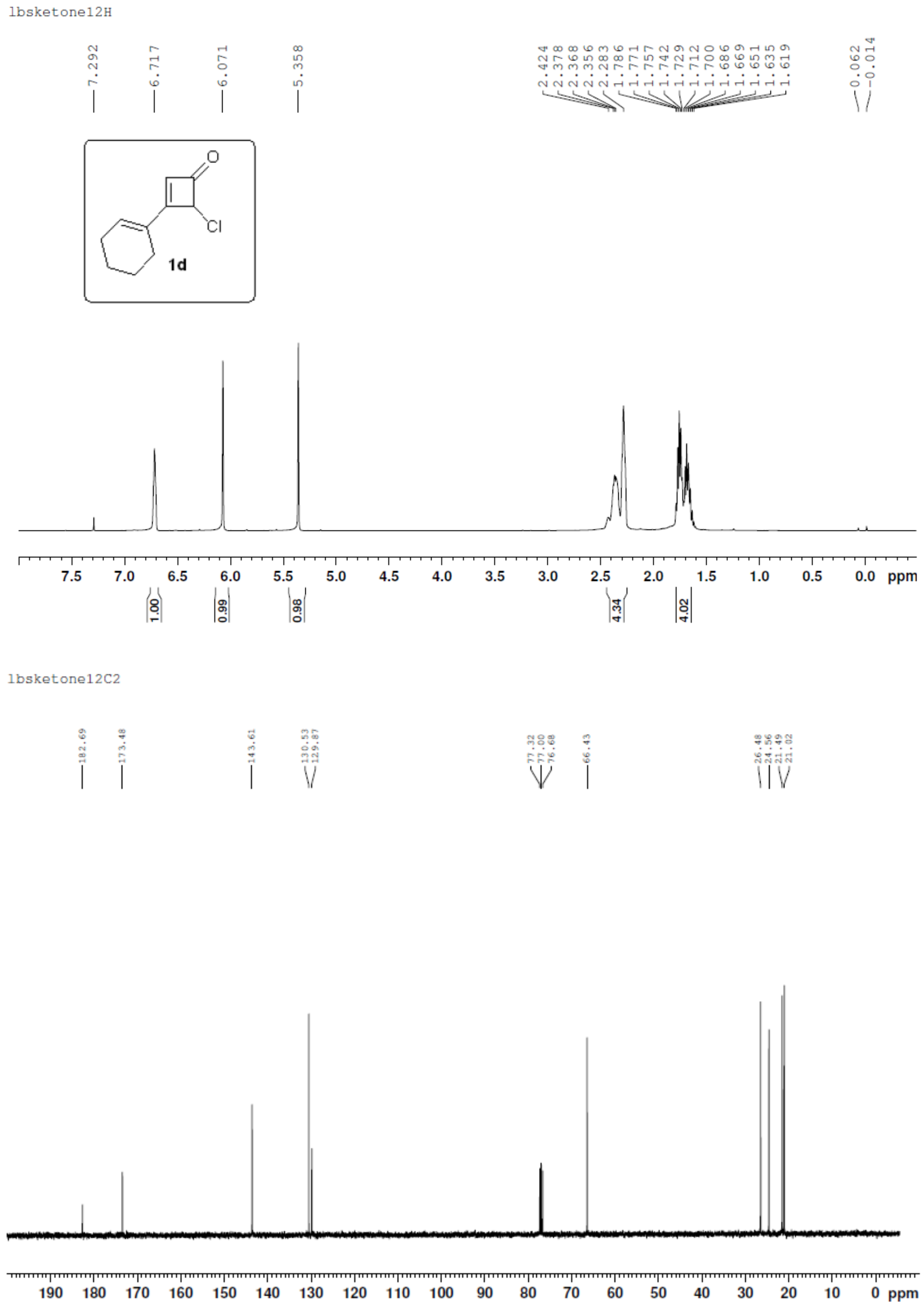

Supplementary figure 2. ^{1}H and ^{13}C NMR spectra for substrate **1d**

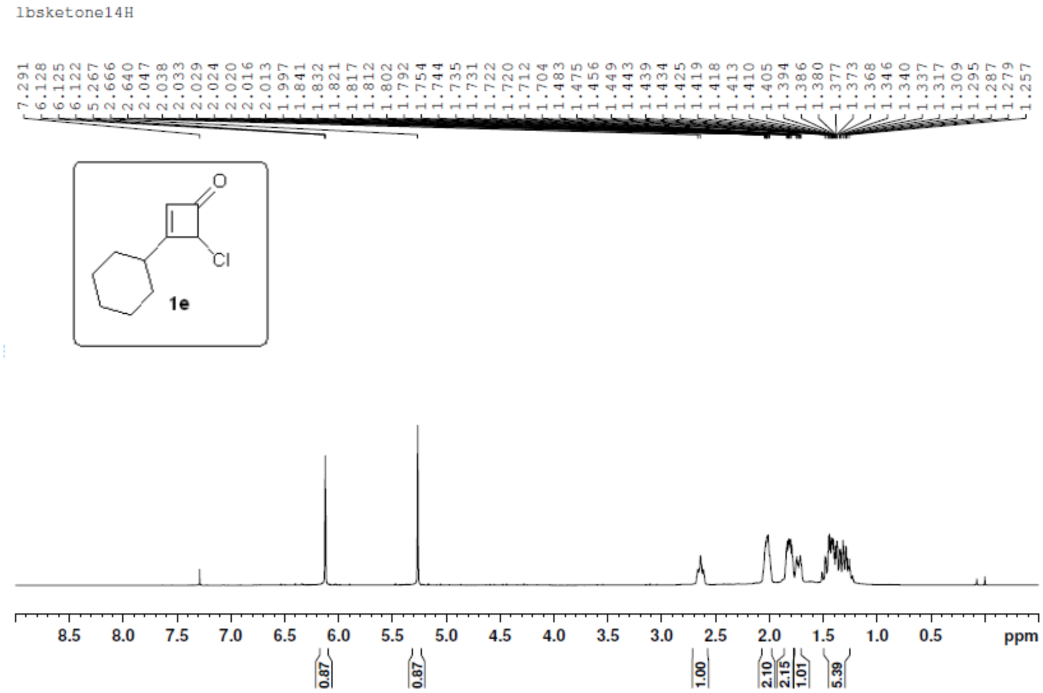

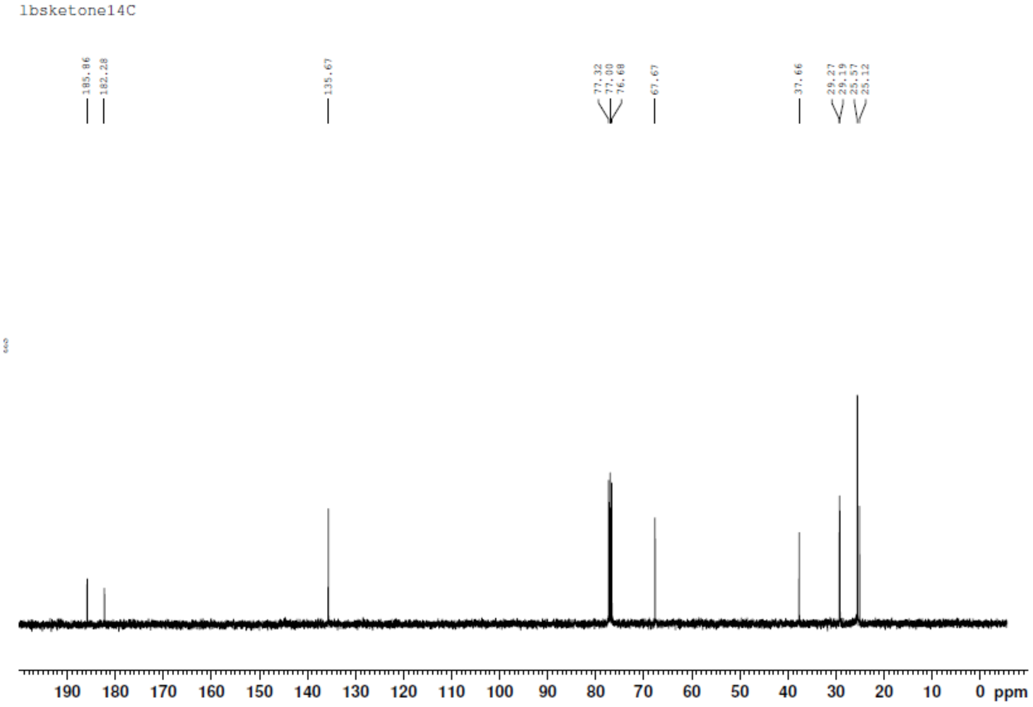

Supplementary figure 3. ^{1}H and ^{13}C NMR spectra for substrate **1e**

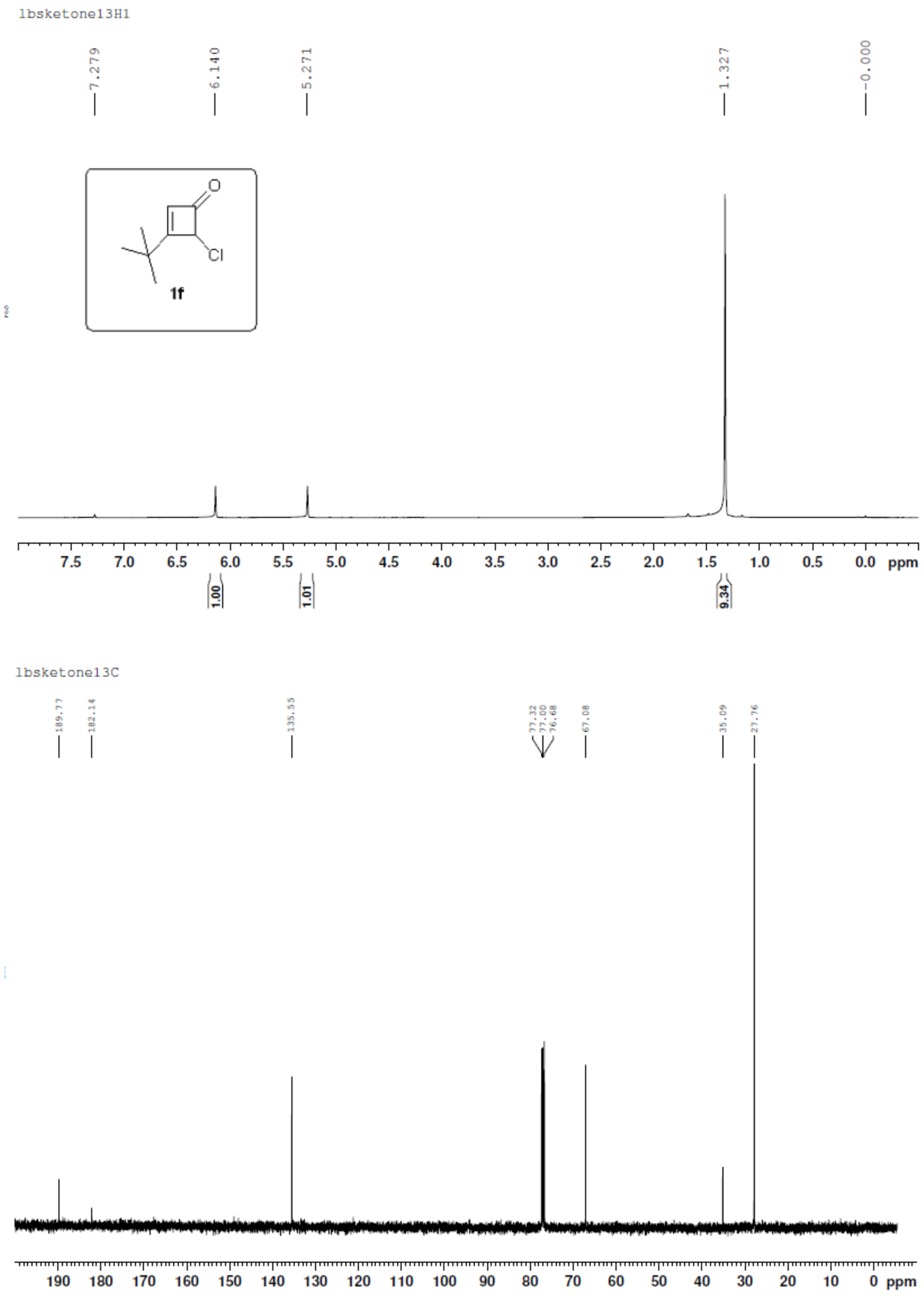

Supplementary figure 4. ^{1}H and ^{13}C NMR spectra for substrate **1f**

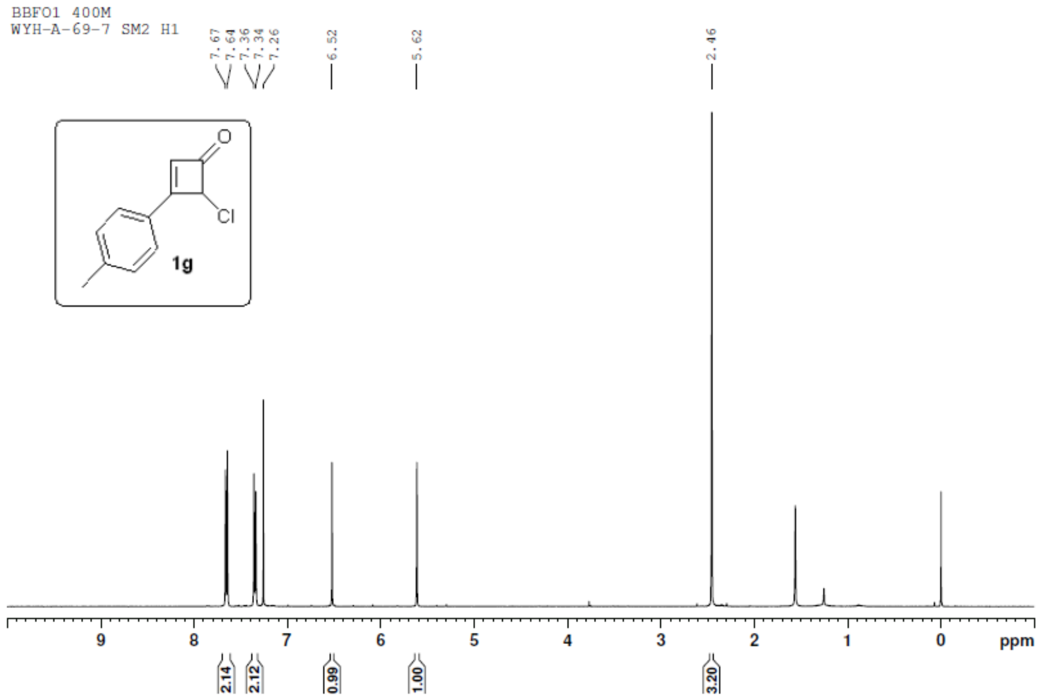

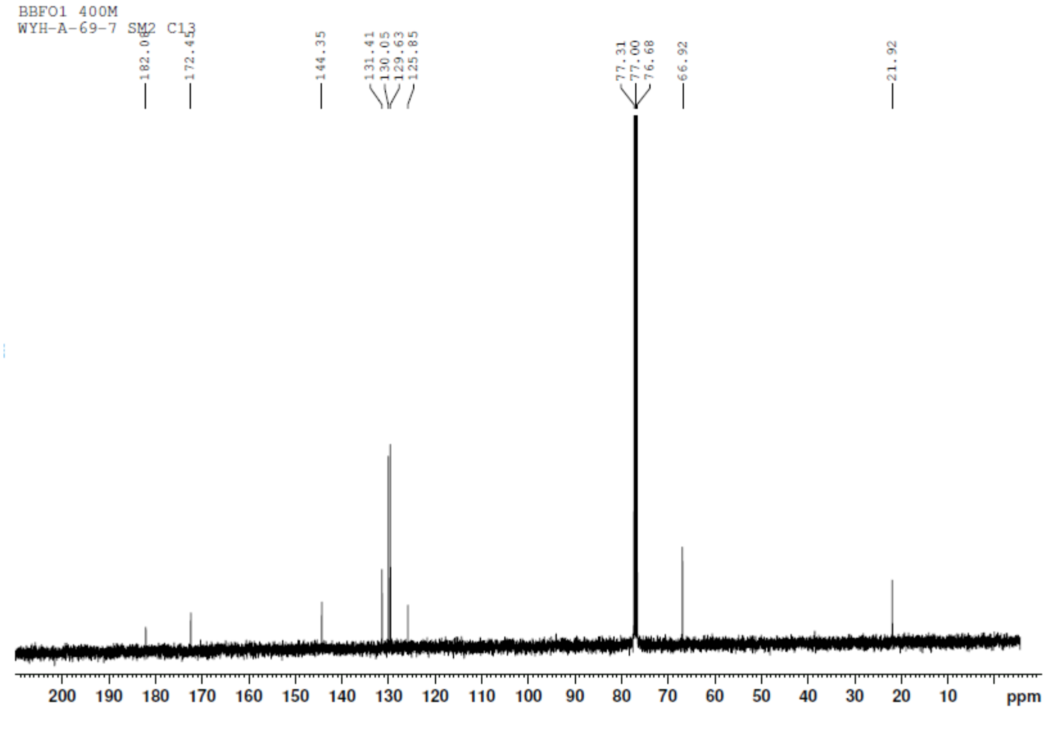

Supplementary figure 5. ^{1}H and ^{13}C NMR spectra for substrate **1g**

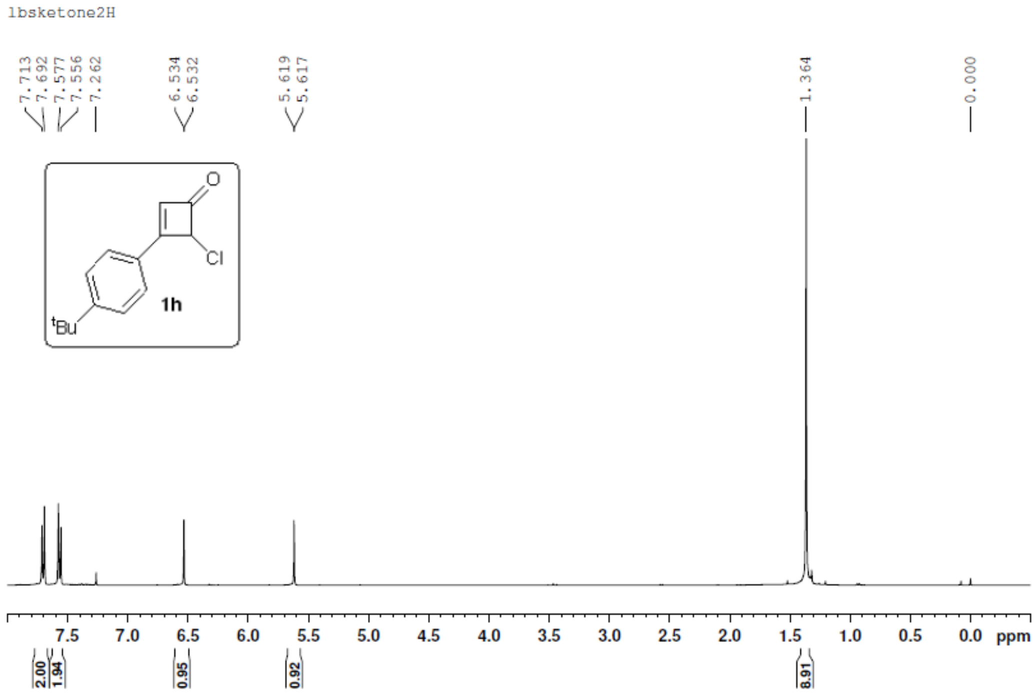

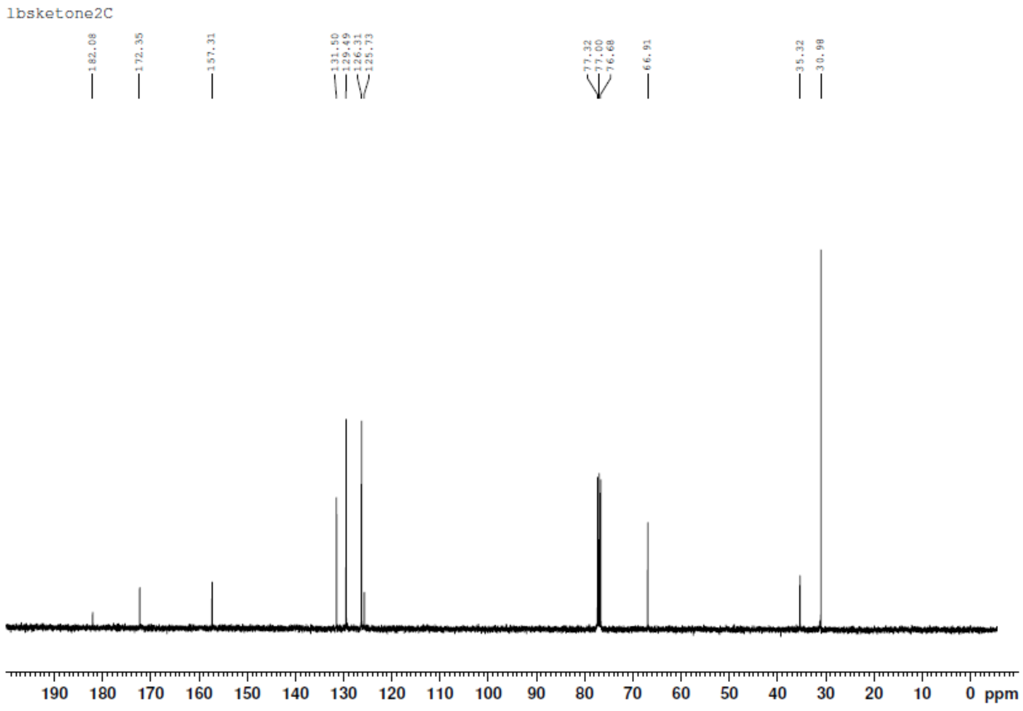

Supplementary figure 6. ^{1}H and ^{13}C NMR spectra for substrate **1h**

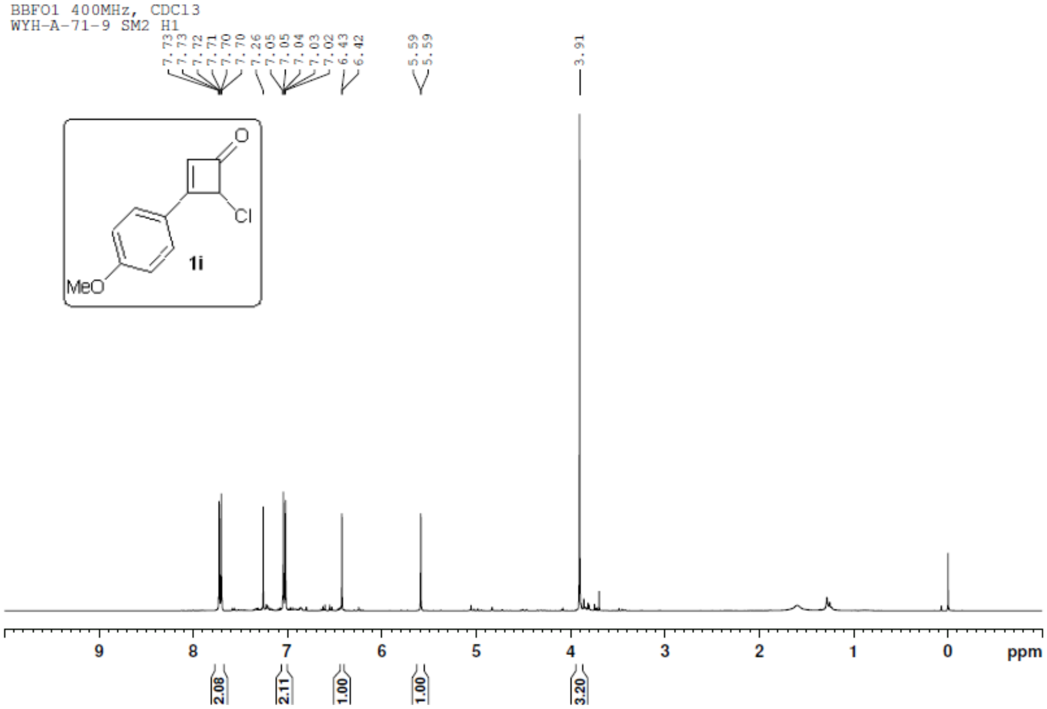

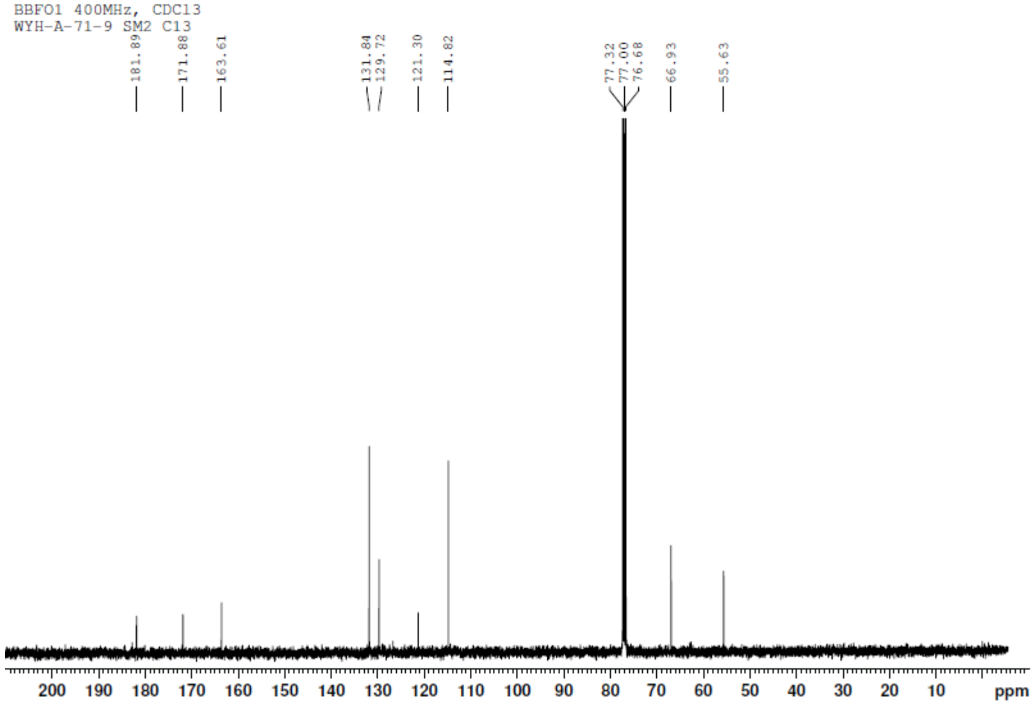

Supplementary figure 7. ^{1}H and ^{13}C NMR spectra for substrate **1i**

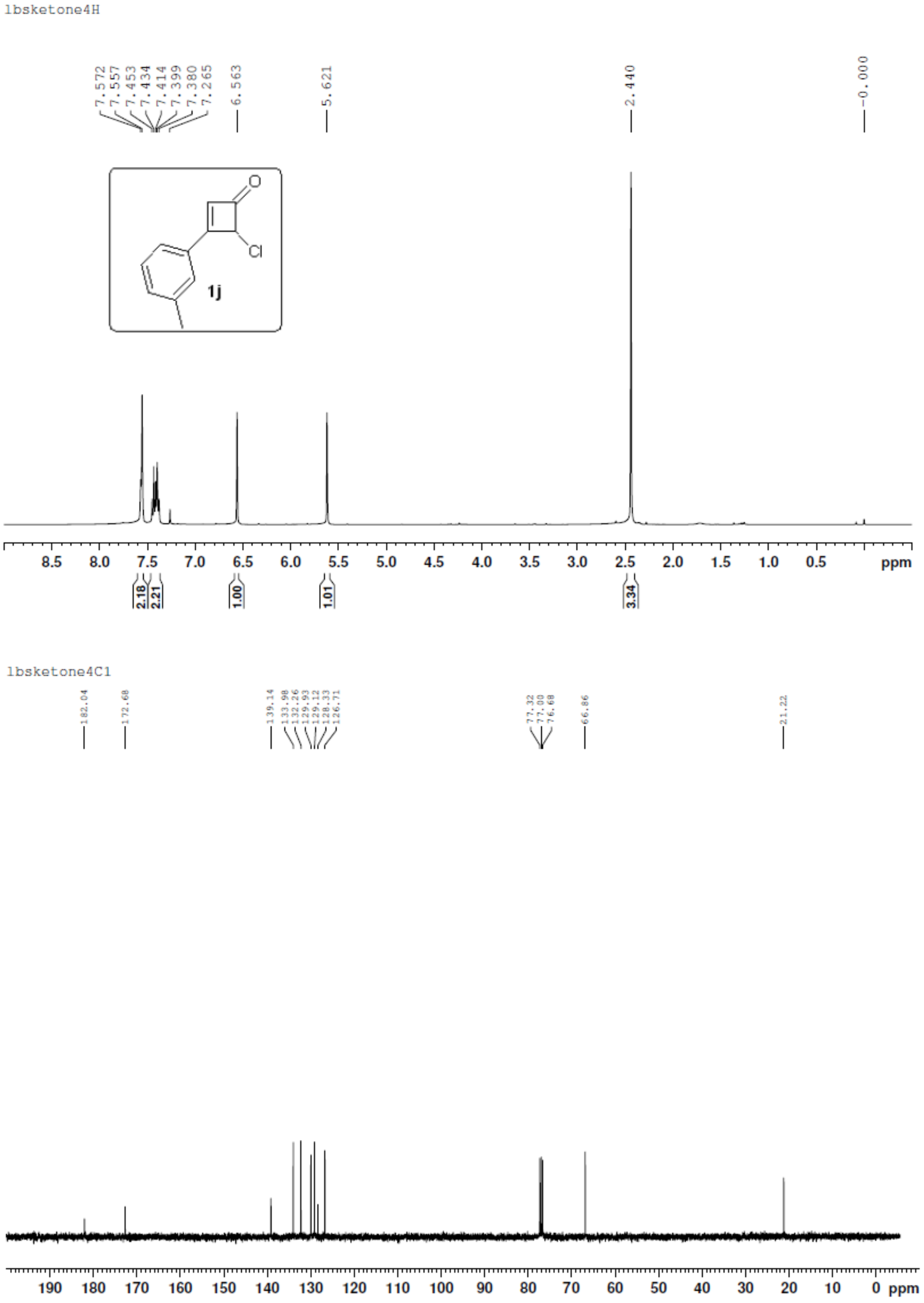

Supplementary figure 8. ^{1}H and ^{13}C NMR spectra for substrate **1j**

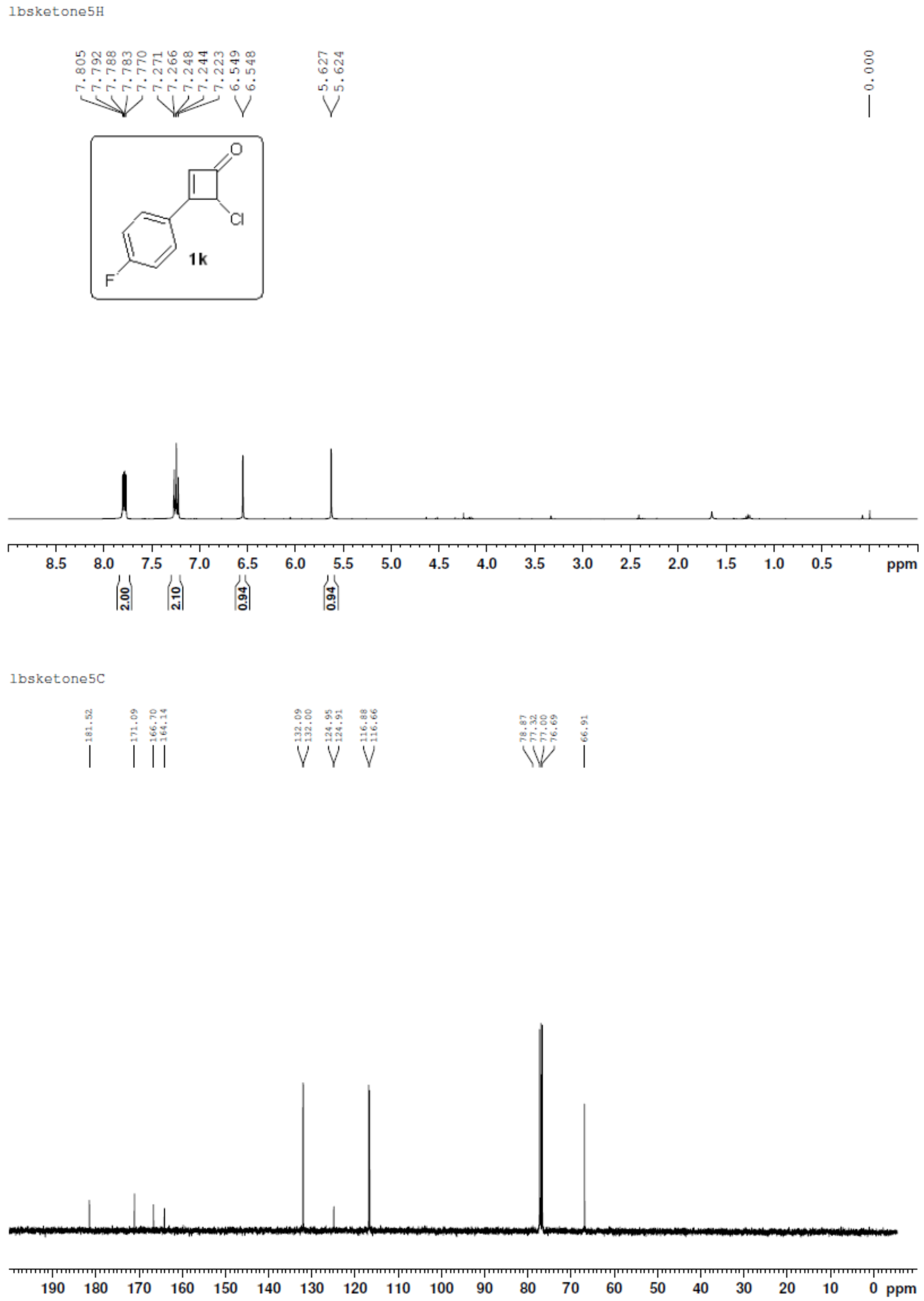

Supplementary figure 9. ^{1}H and ^{13}C NMR spectra for substrate **1k**

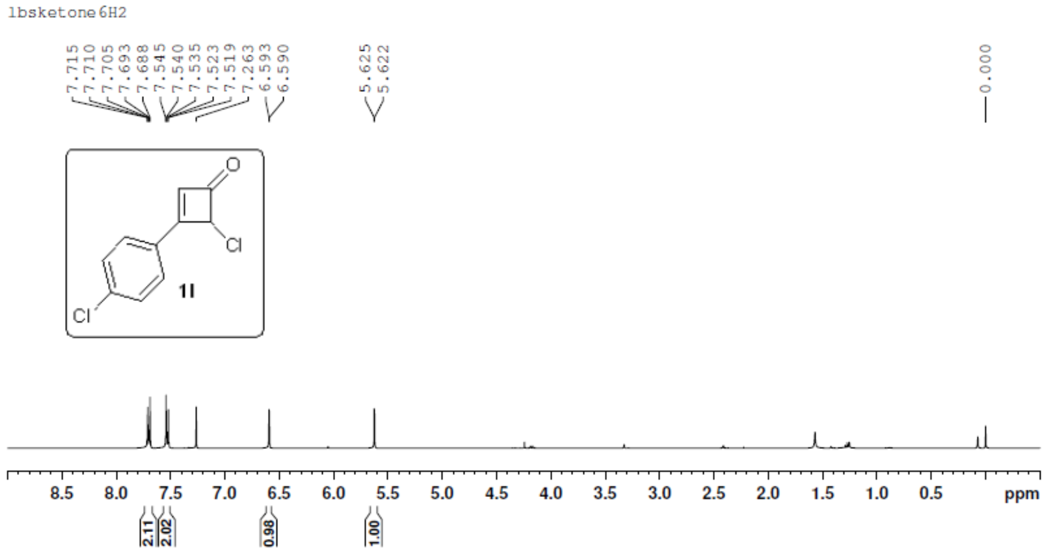

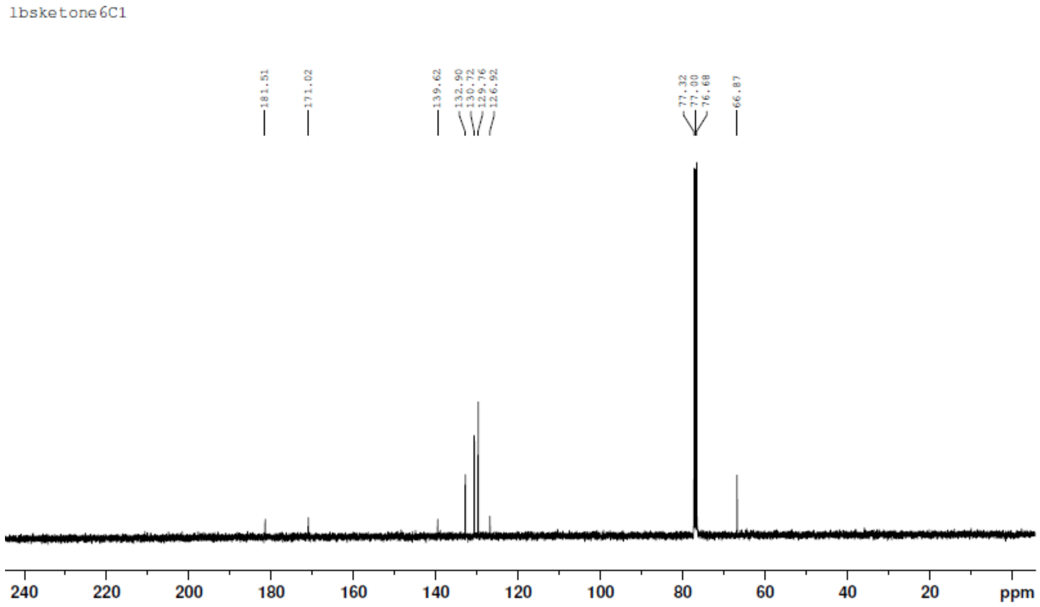

Supplementary figure 10. ^{1}H and ^{13}C NMR spectra for substrate **1l**

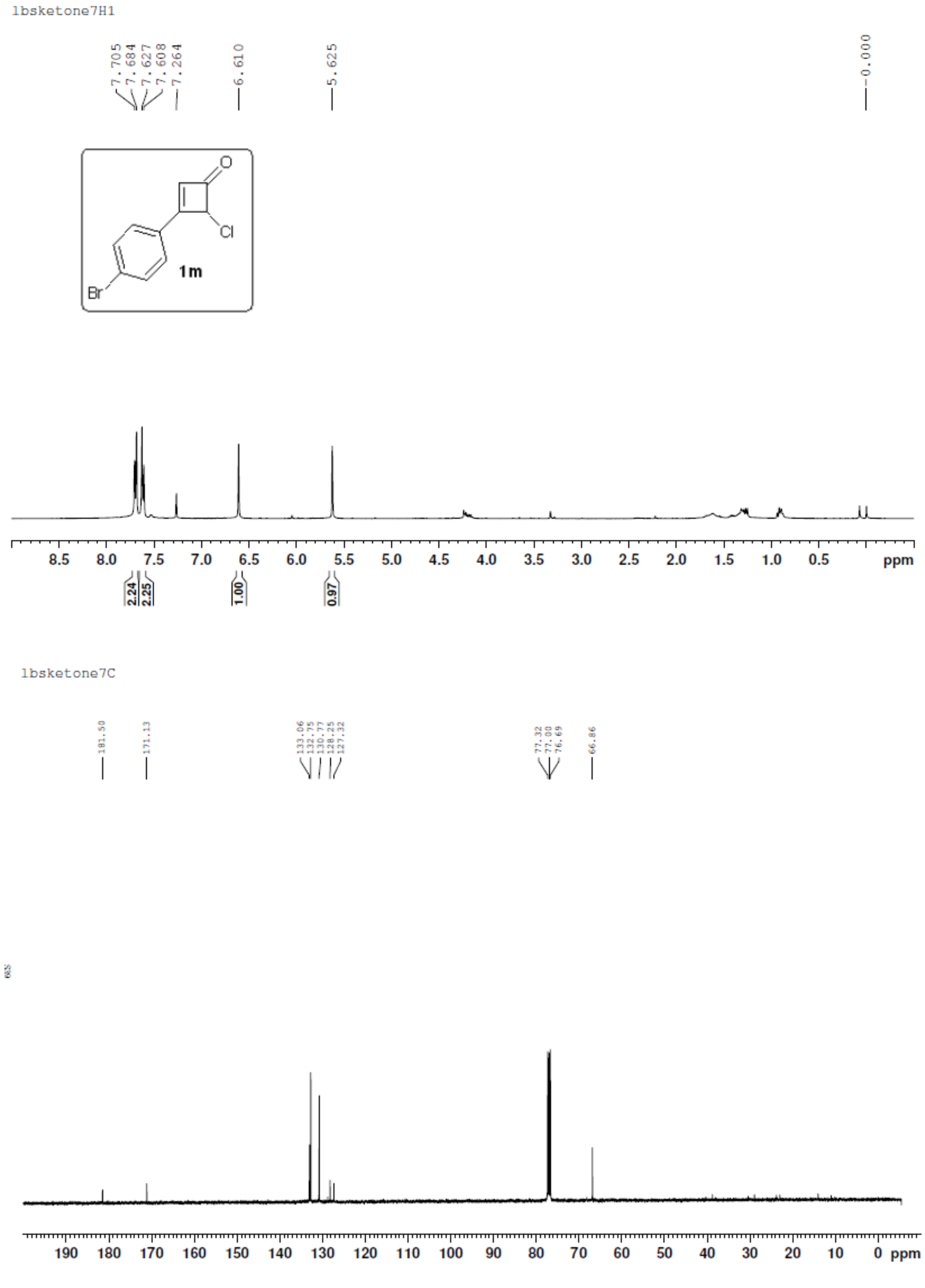

Supplementary figure 11. ^{1}H and ^{13}C NMR spectra for substrate **1m**

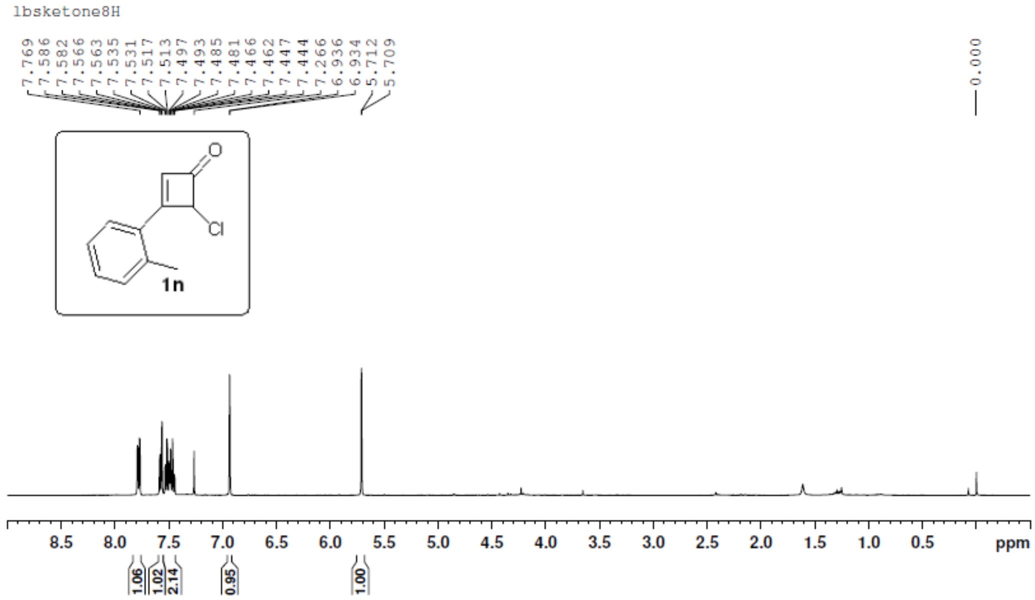

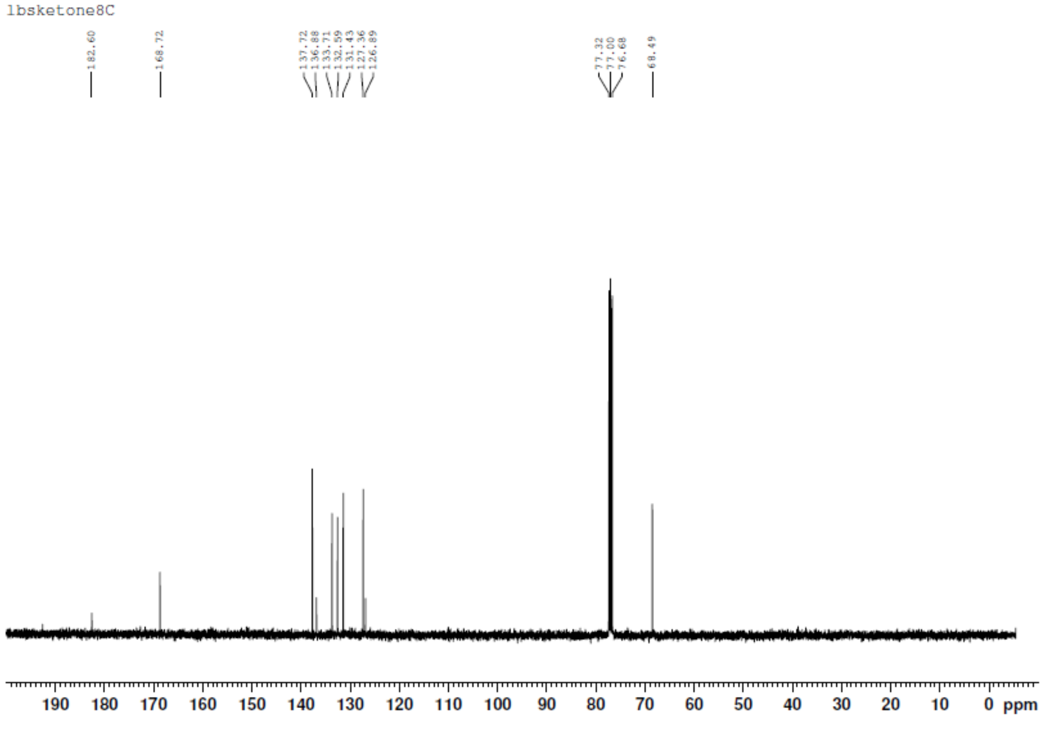

Supplementary figure 12. ^{1}H and ^{13}C NMR spectra for substrate **1n**

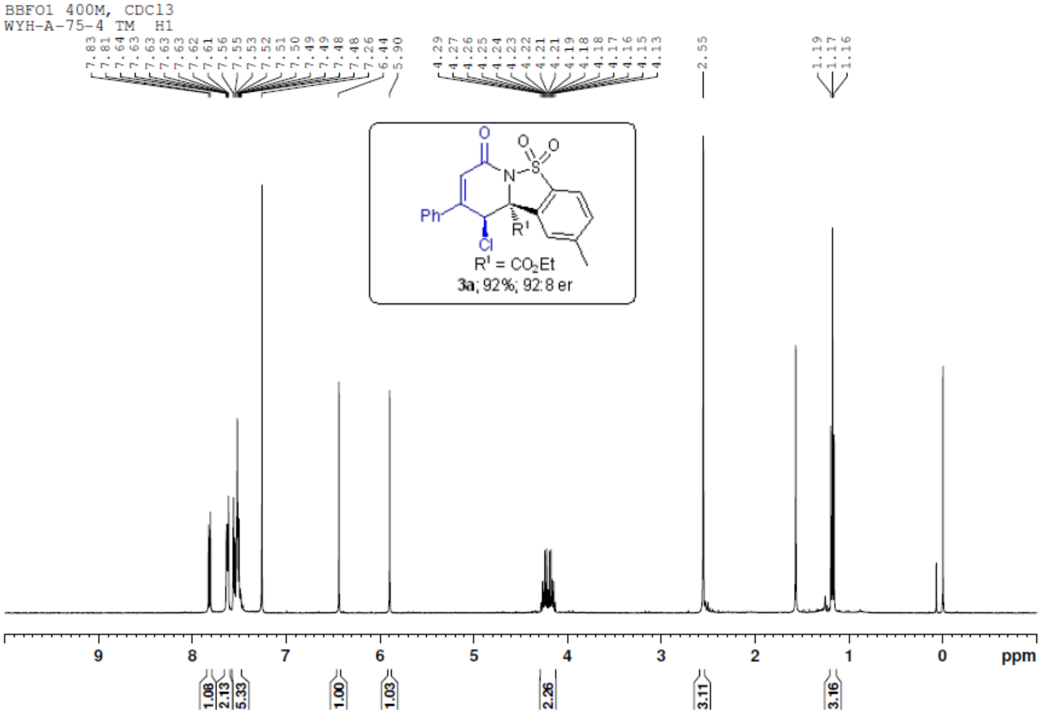

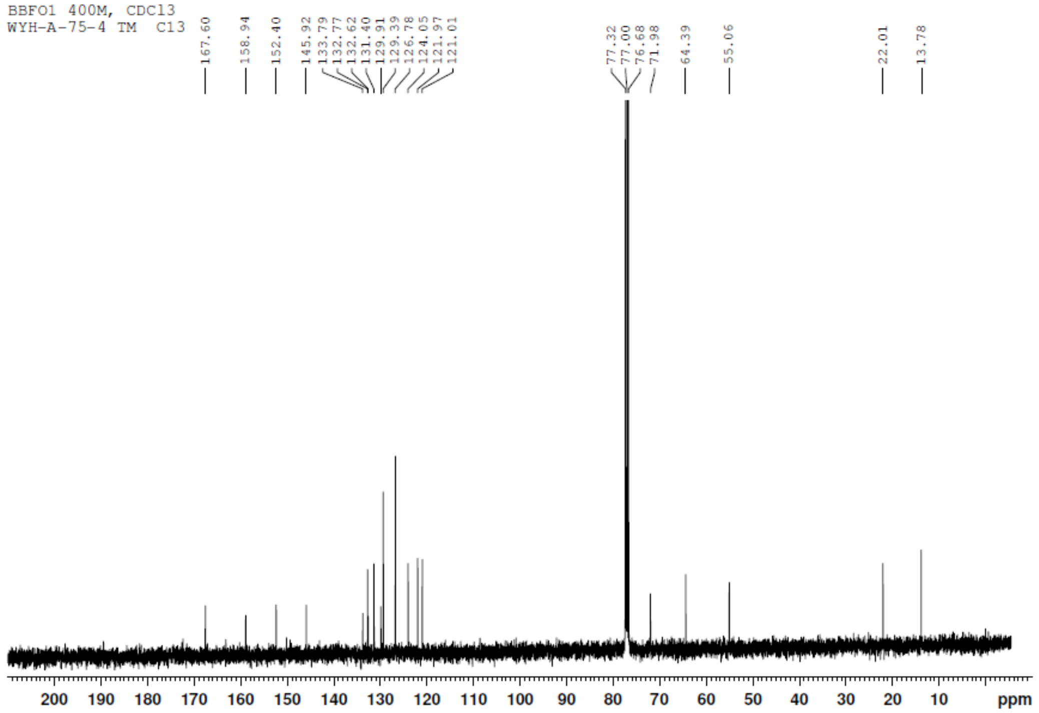

Supplementary figure 13. ^{1}H and ^{13}C NMR spectra for product **3a**

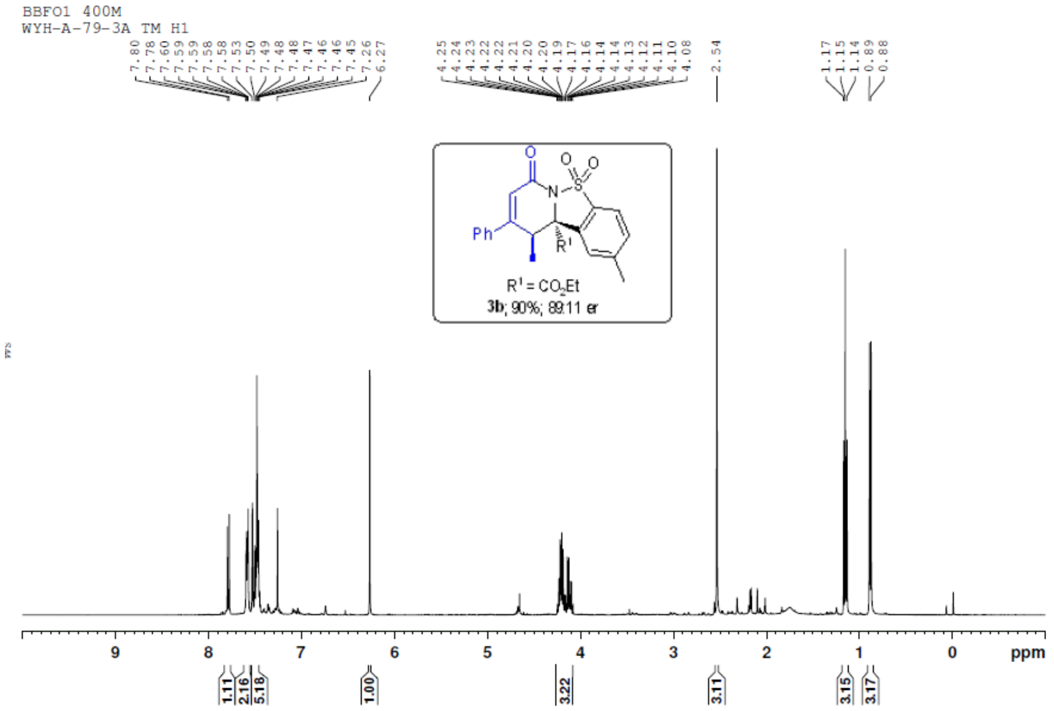

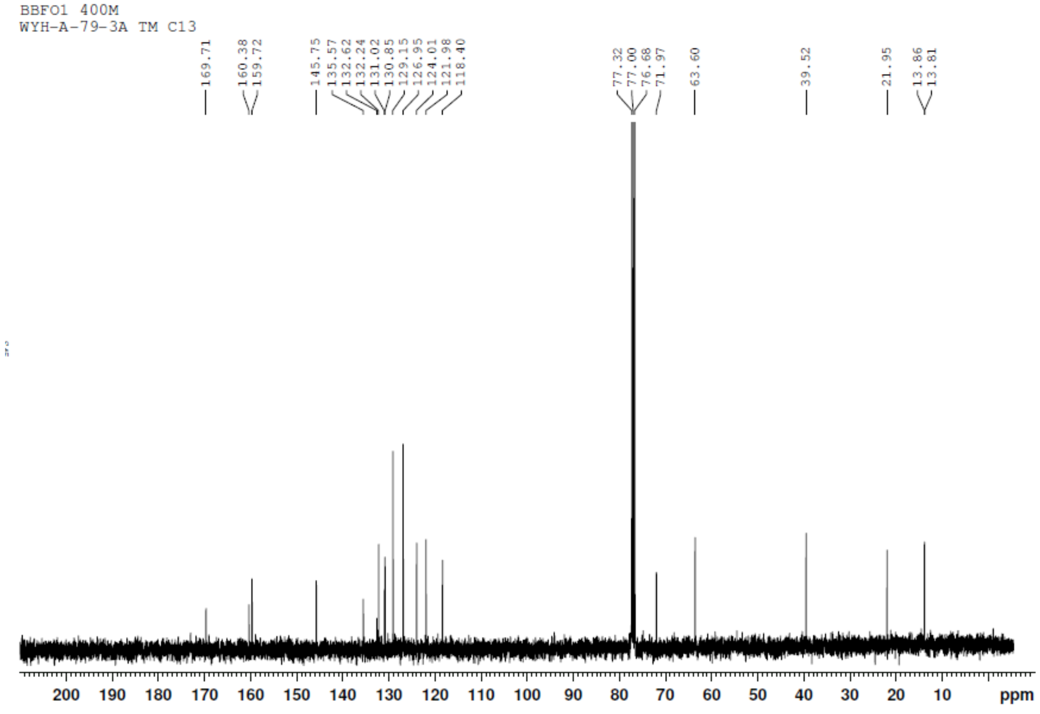

Supplementary figure 14. ^{1}H and ^{13}C NMR spectra for product **3b**

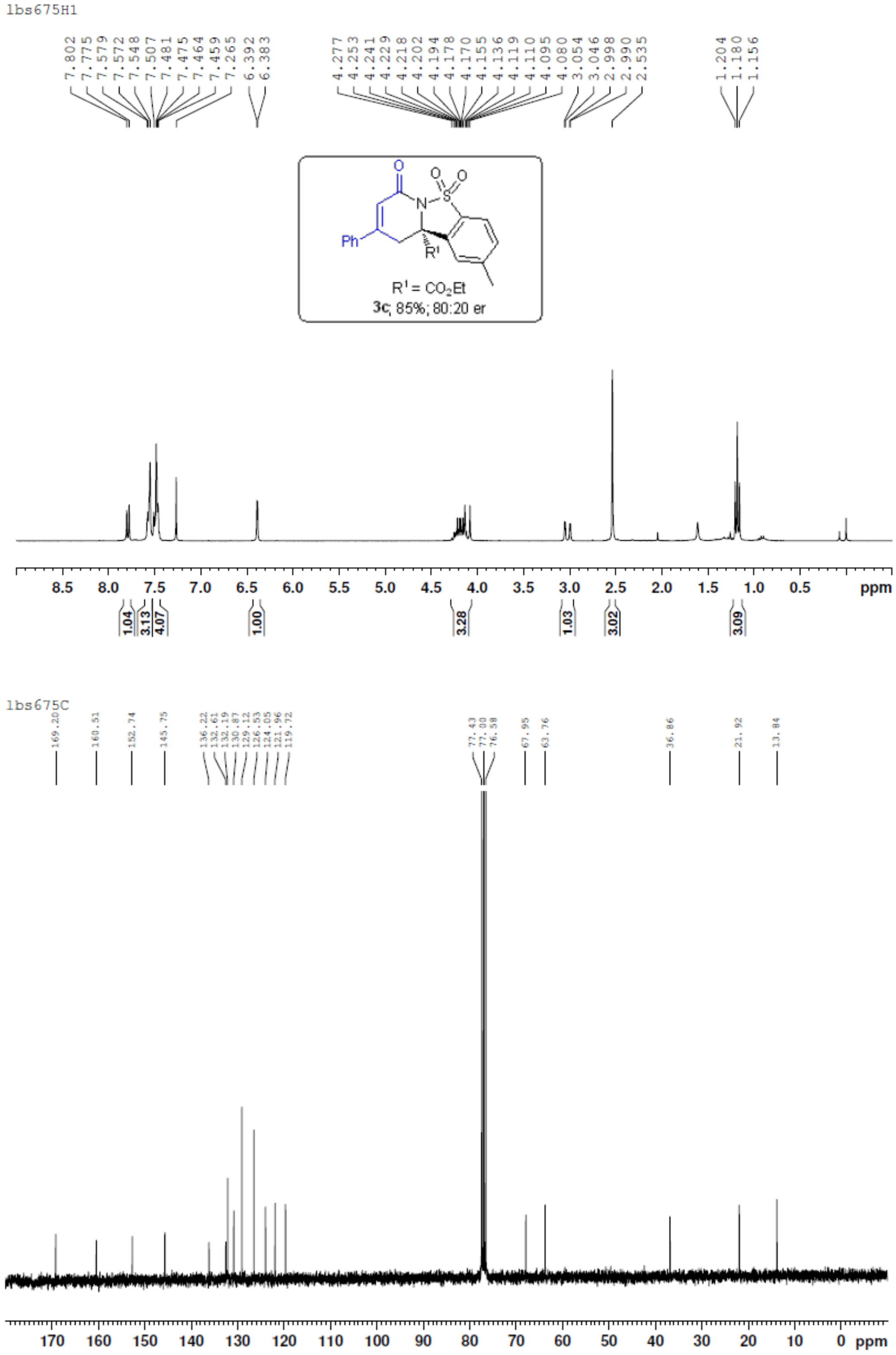

Supplementary figure 15. ^{1}H and ^{13}C NMR spectra for product **3c**

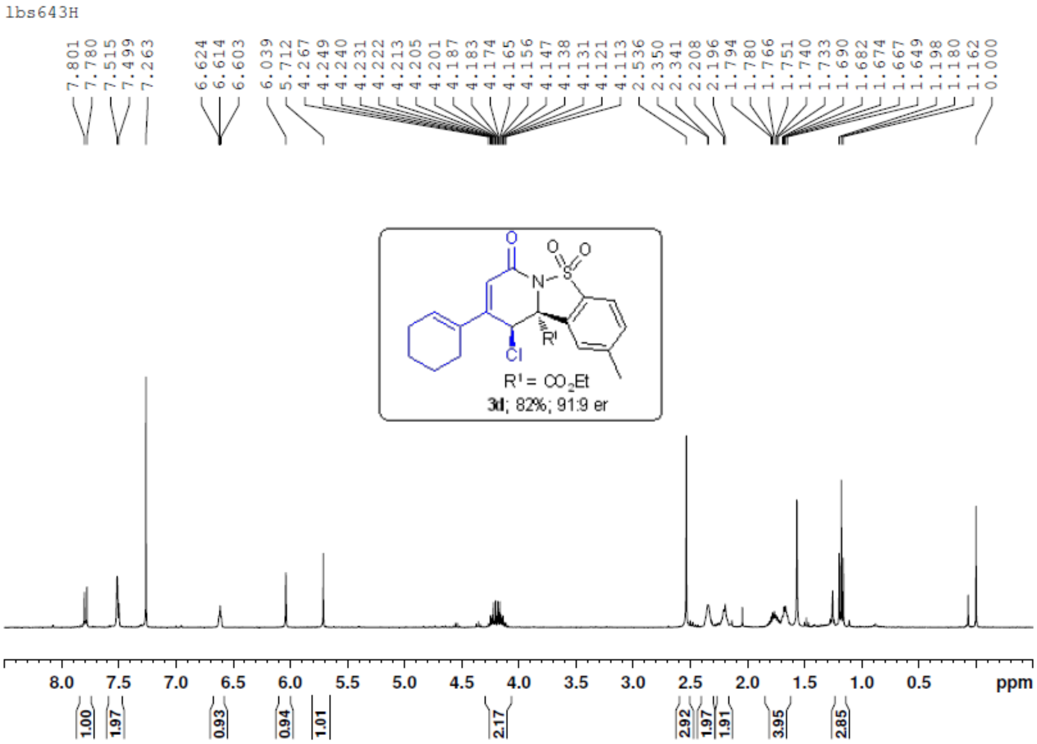

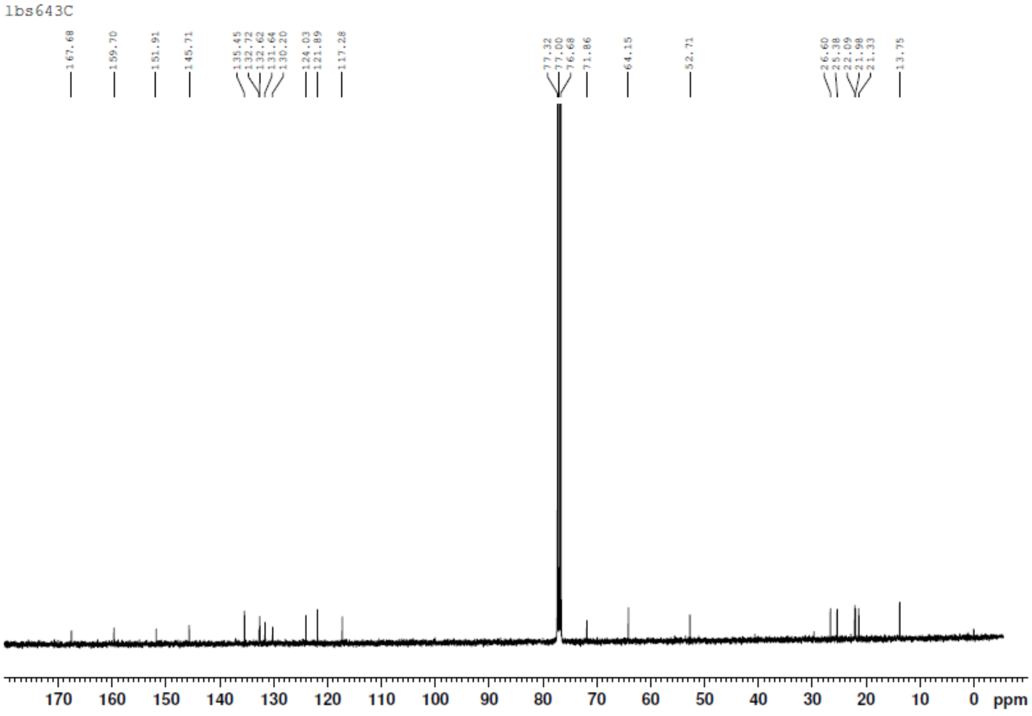

Supplementary figure 16. ^{1}H and ^{13}C NMR spectra for product **3d**

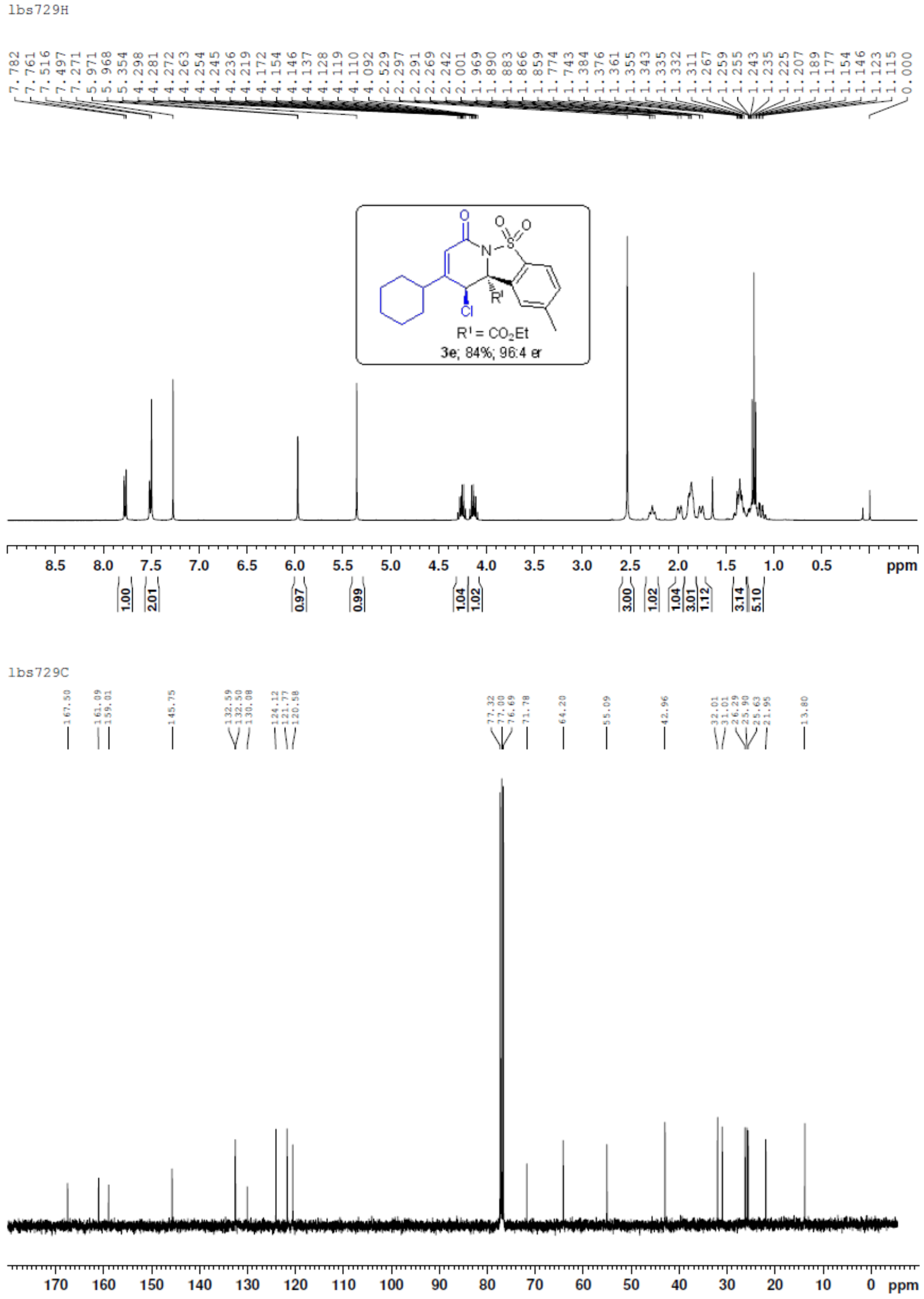

Supplementary figure 17. ^{1}H and ^{13}C NMR spectra for product **3e**

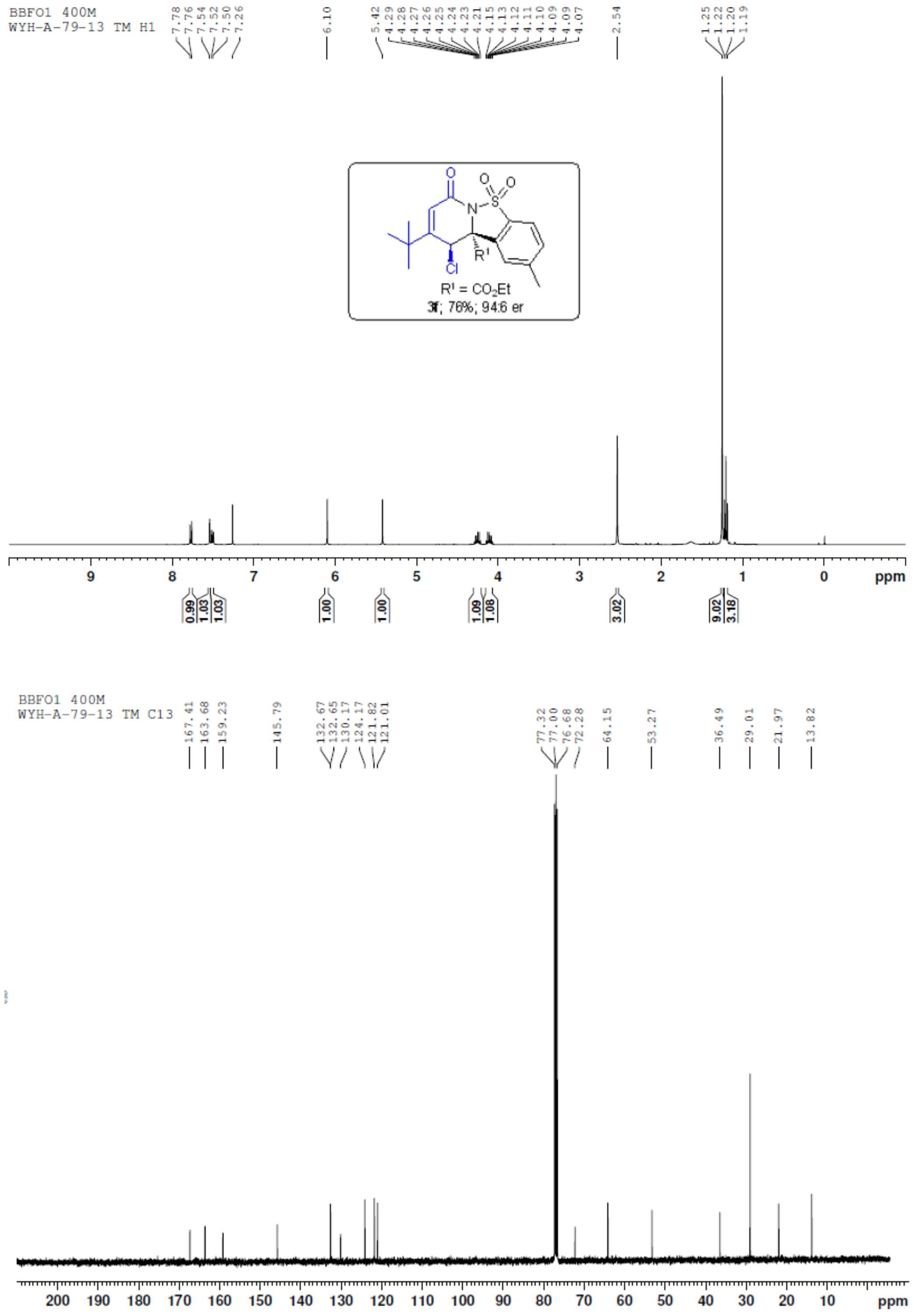

Supplementary figure 18. ^{1}H and ^{13}C NMR spectra for product **3f**

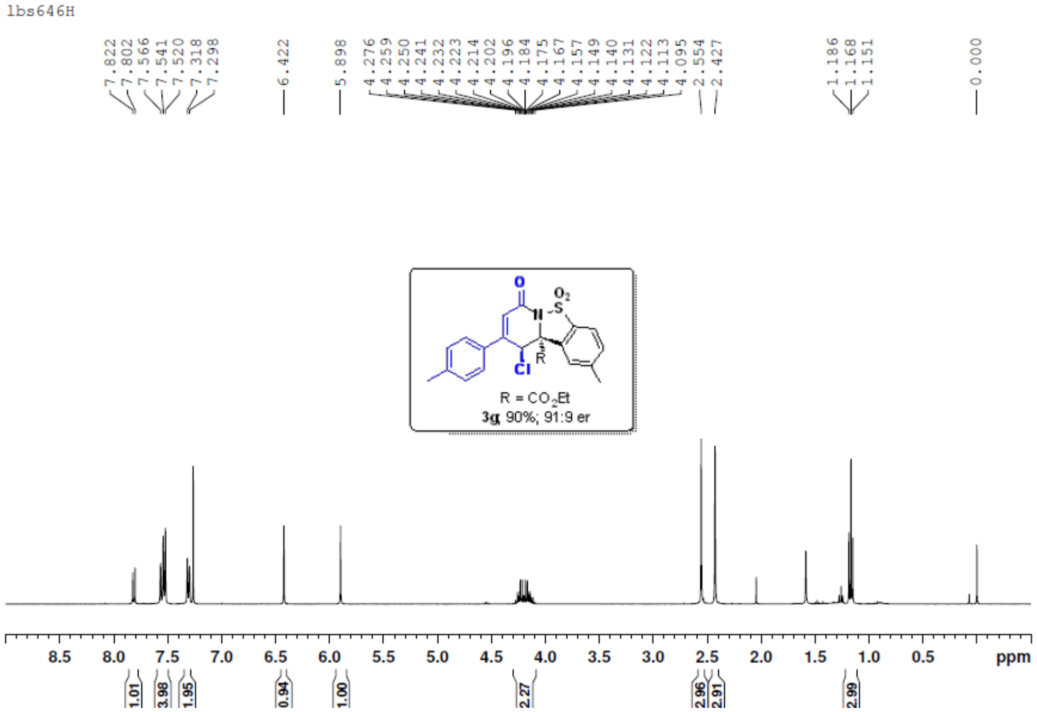

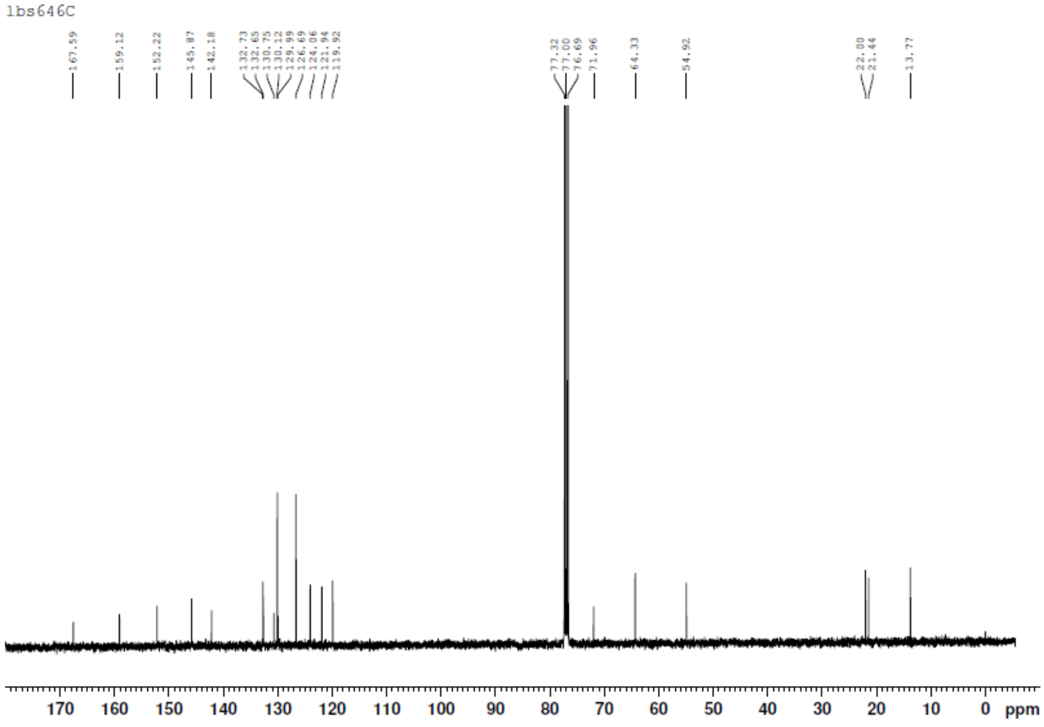

Supplementary figure 19. ^{1}H and ^{13}C NMR spectra for product **3g**

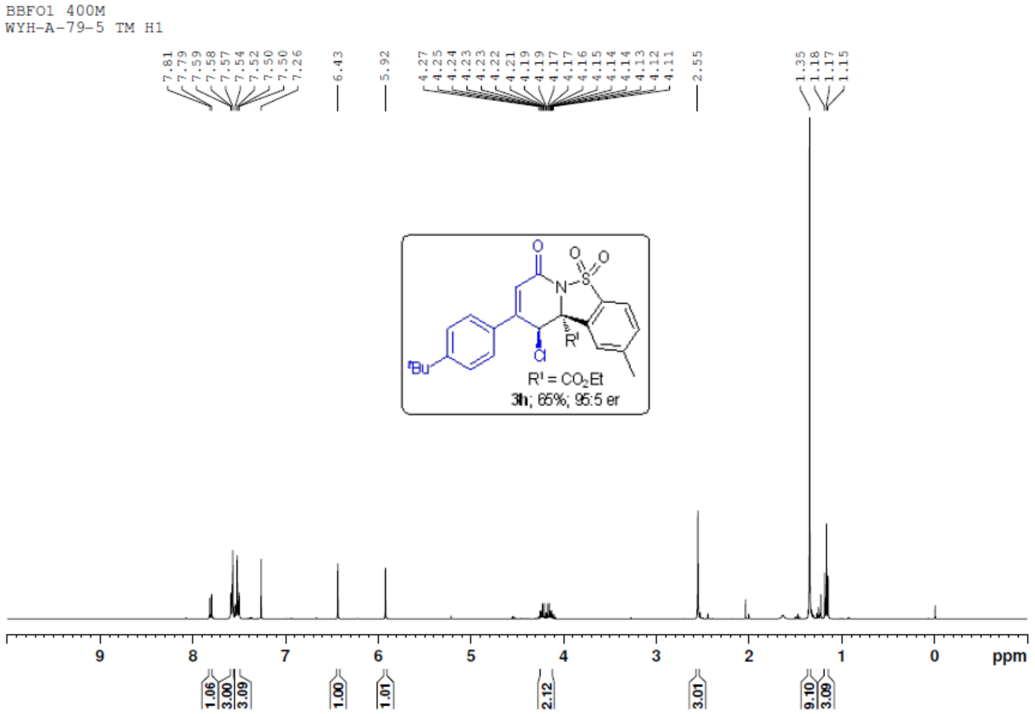

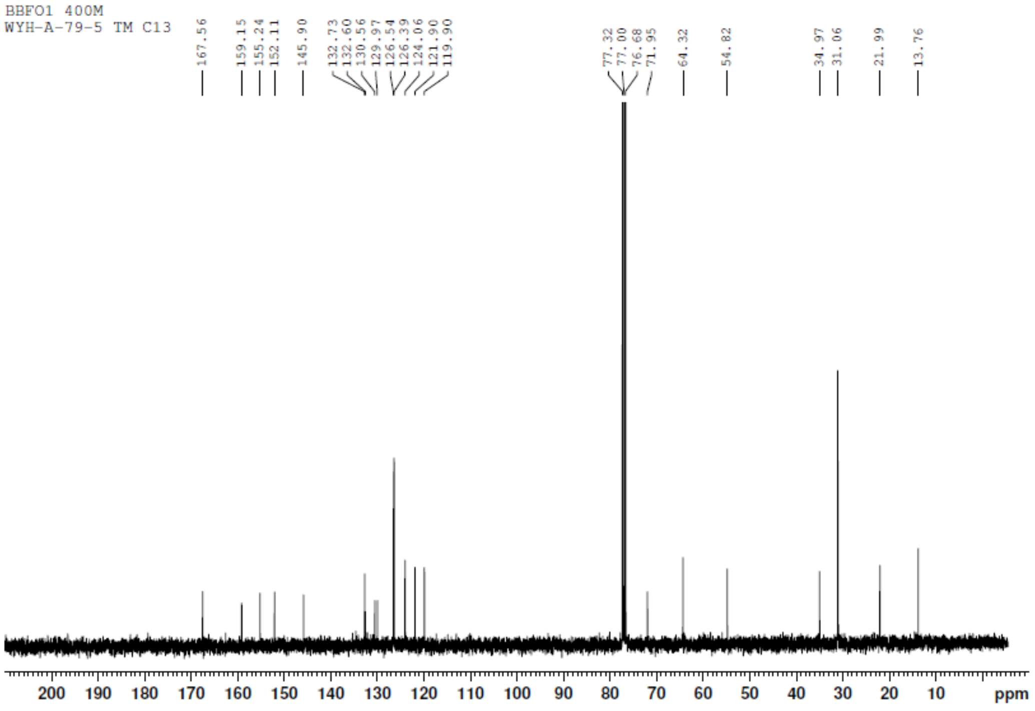

Supplementary figure 20. ^{1}H and ^{13}C NMR spectra for product **3h**

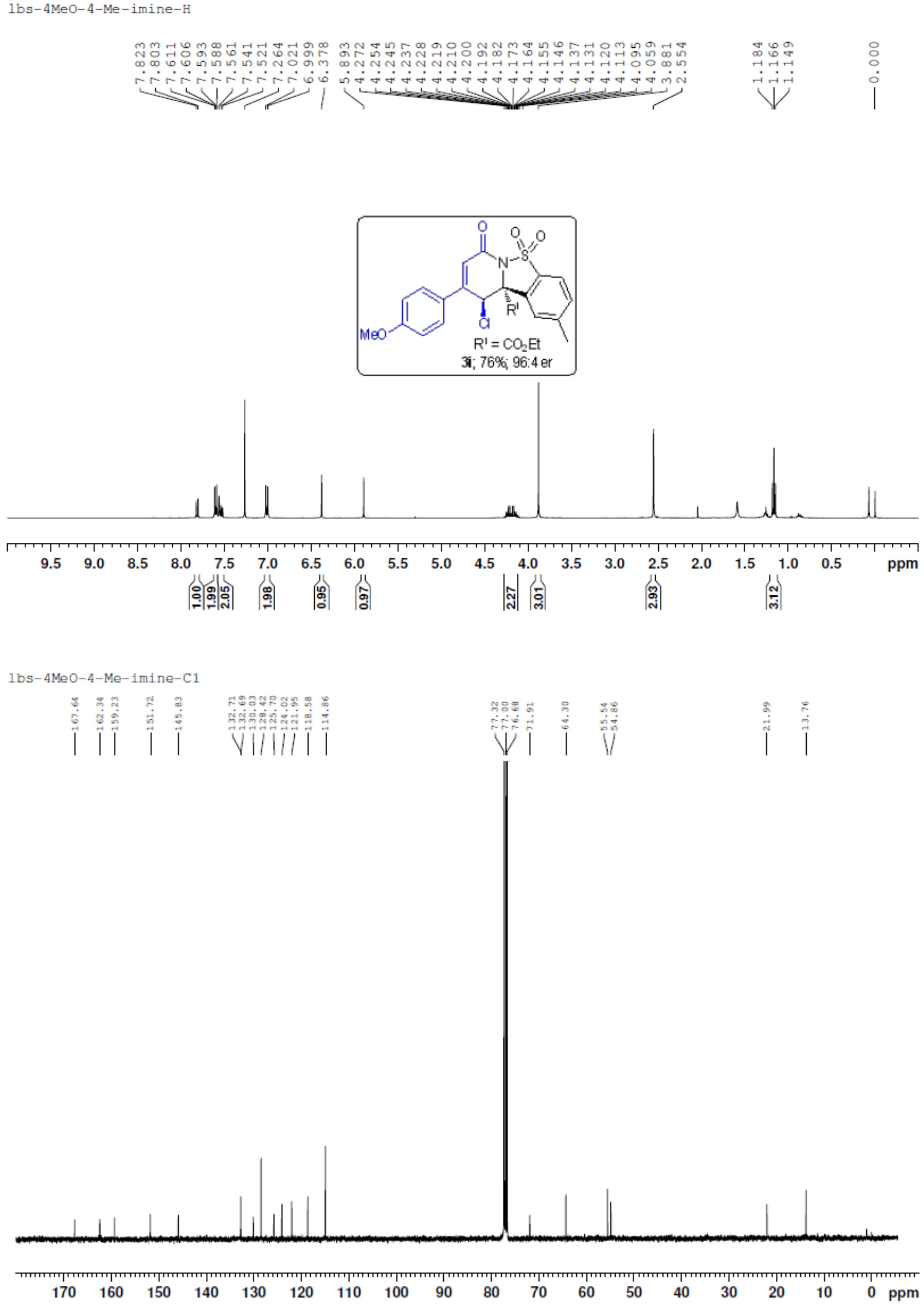

Supplementary figure 21. ^{1}H and ^{13}C NMR spectra for product **3i**

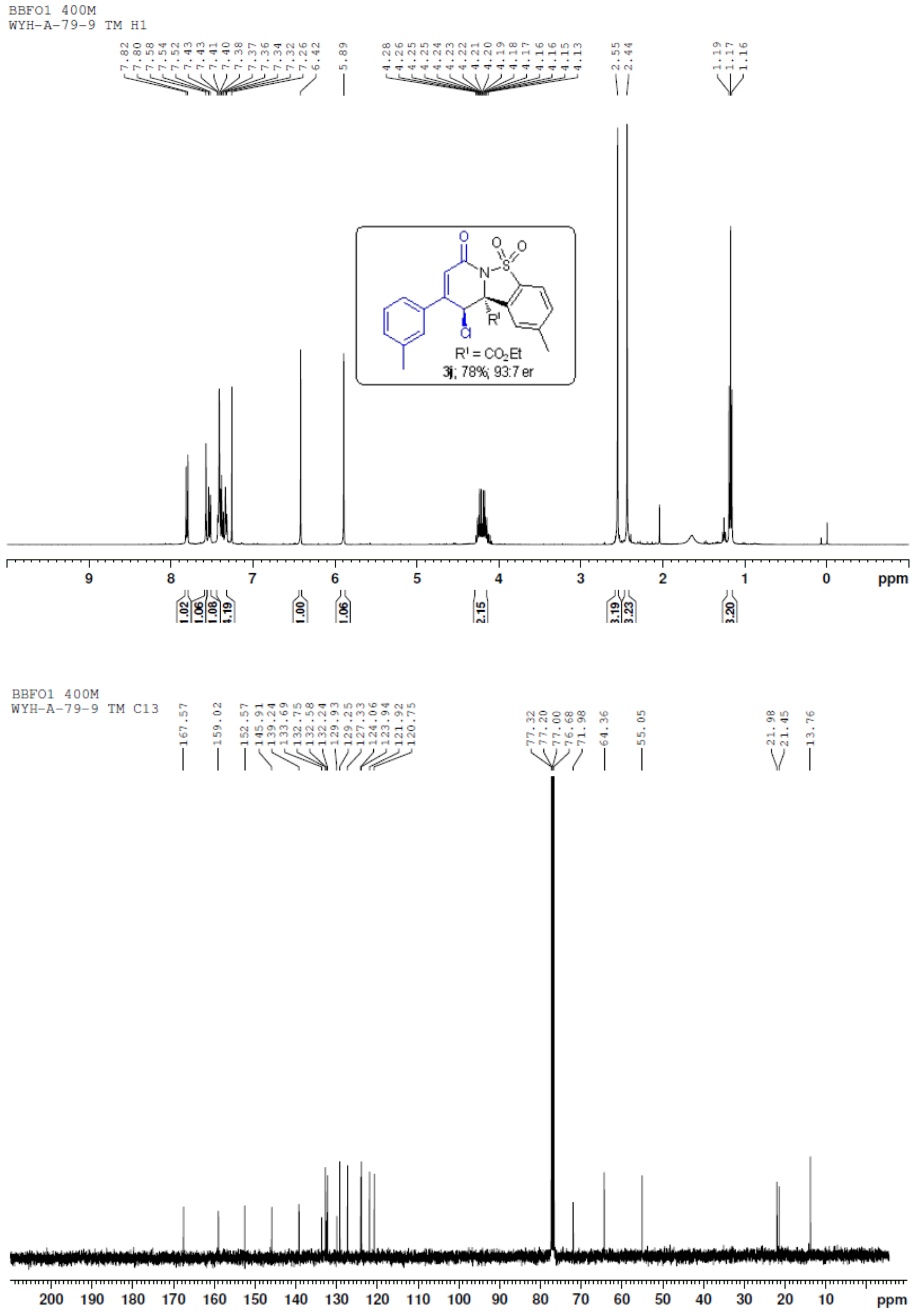

Supplementary figure 22. ^{1}H and ^{13}C NMR spectra for product **3j**

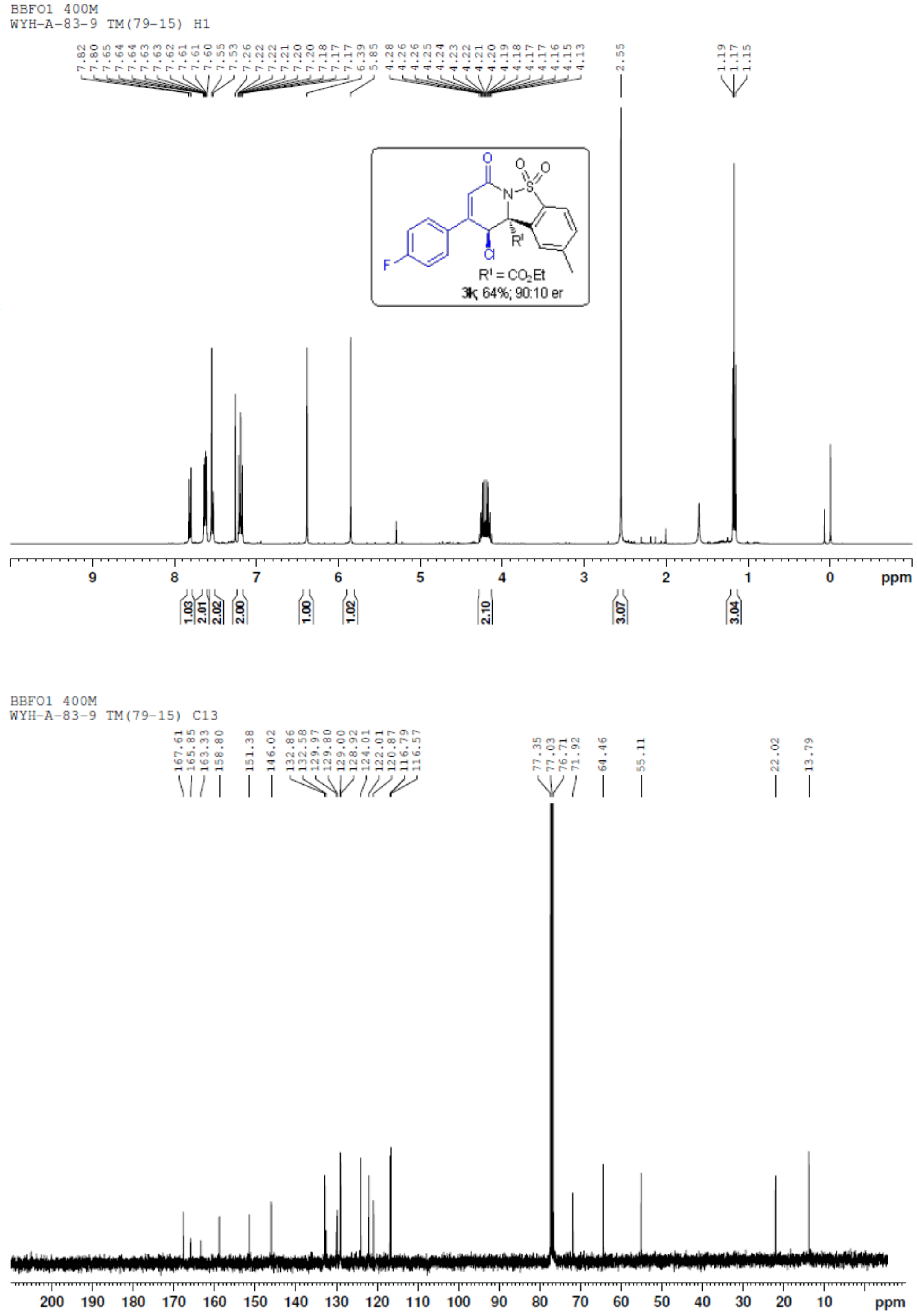

Supplementary figure 23. ^{1}H and ^{13}C NMR spectra for product **3k**

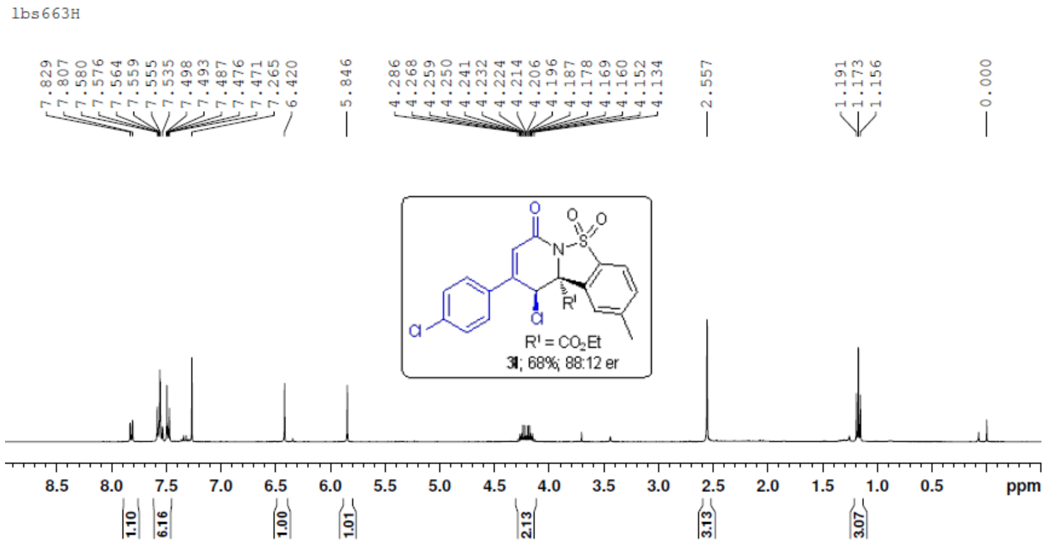

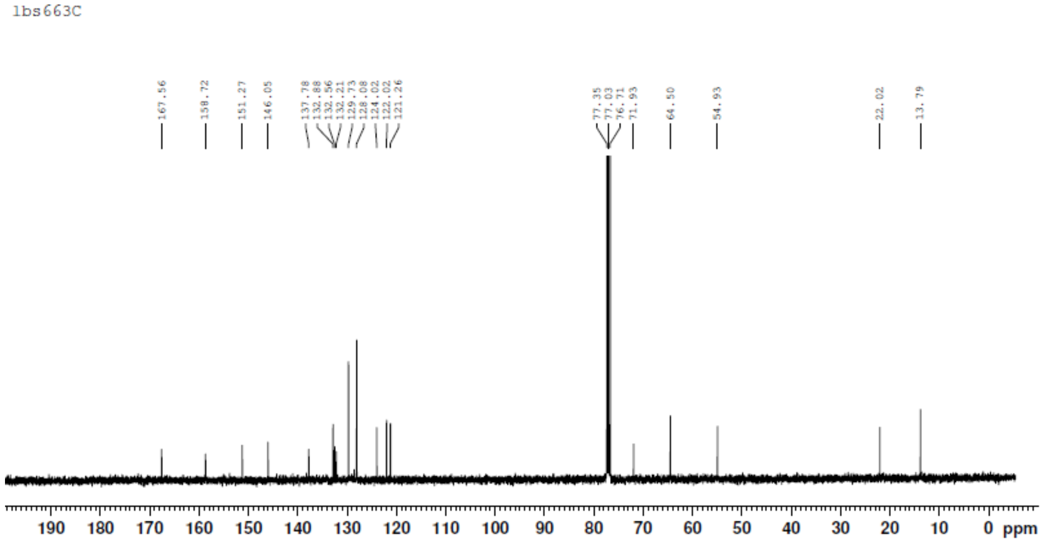

Supplementary figure 24. ^{1}H and ^{13}C NMR spectra for product **3l**

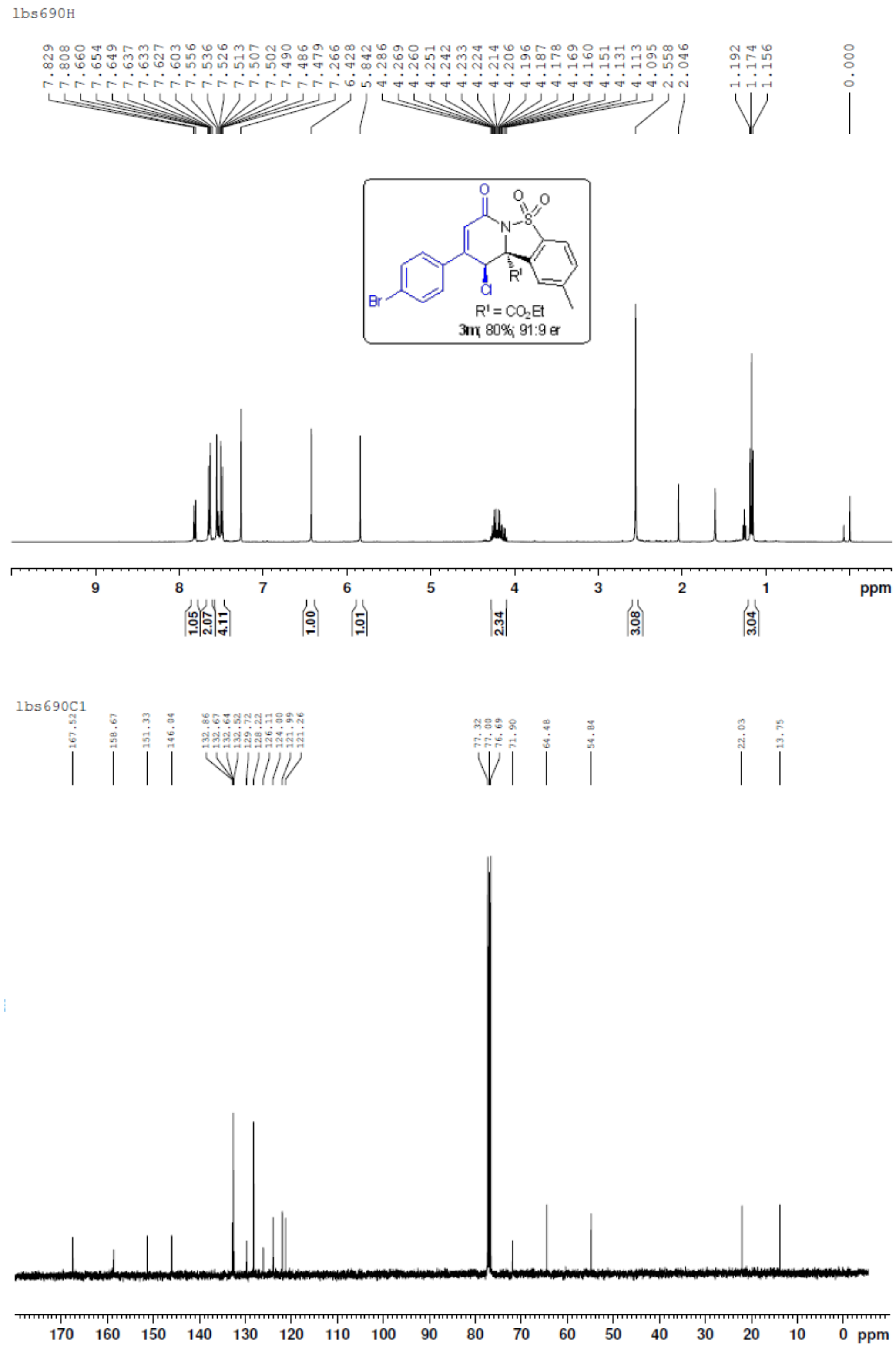

Supplementary figure 25. ^{1}H and ^{13}C NMR spectra for product **3m**

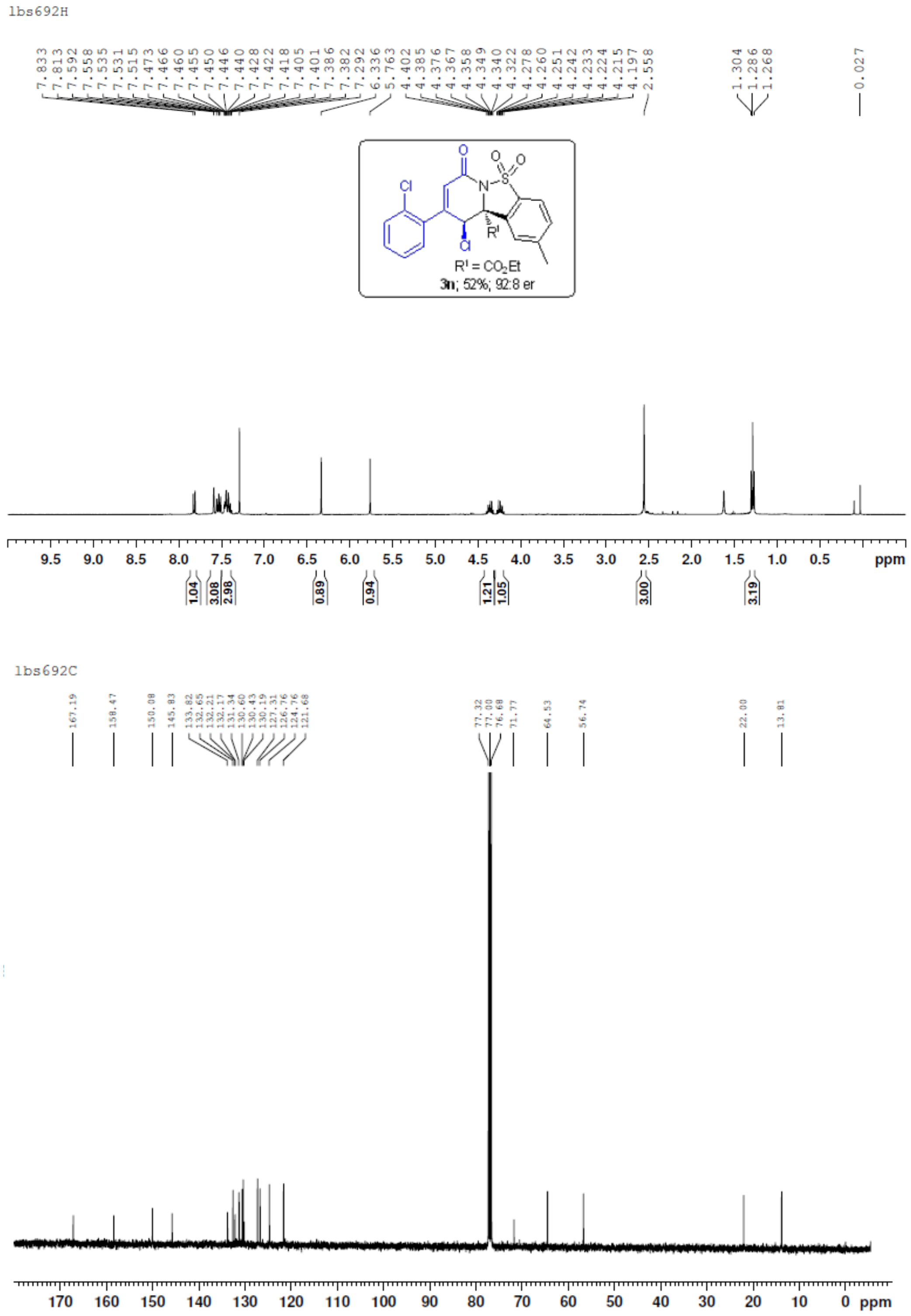

Supplementary figure 26. ^{1}H and ^{13}C NMR spectra for product **3n**

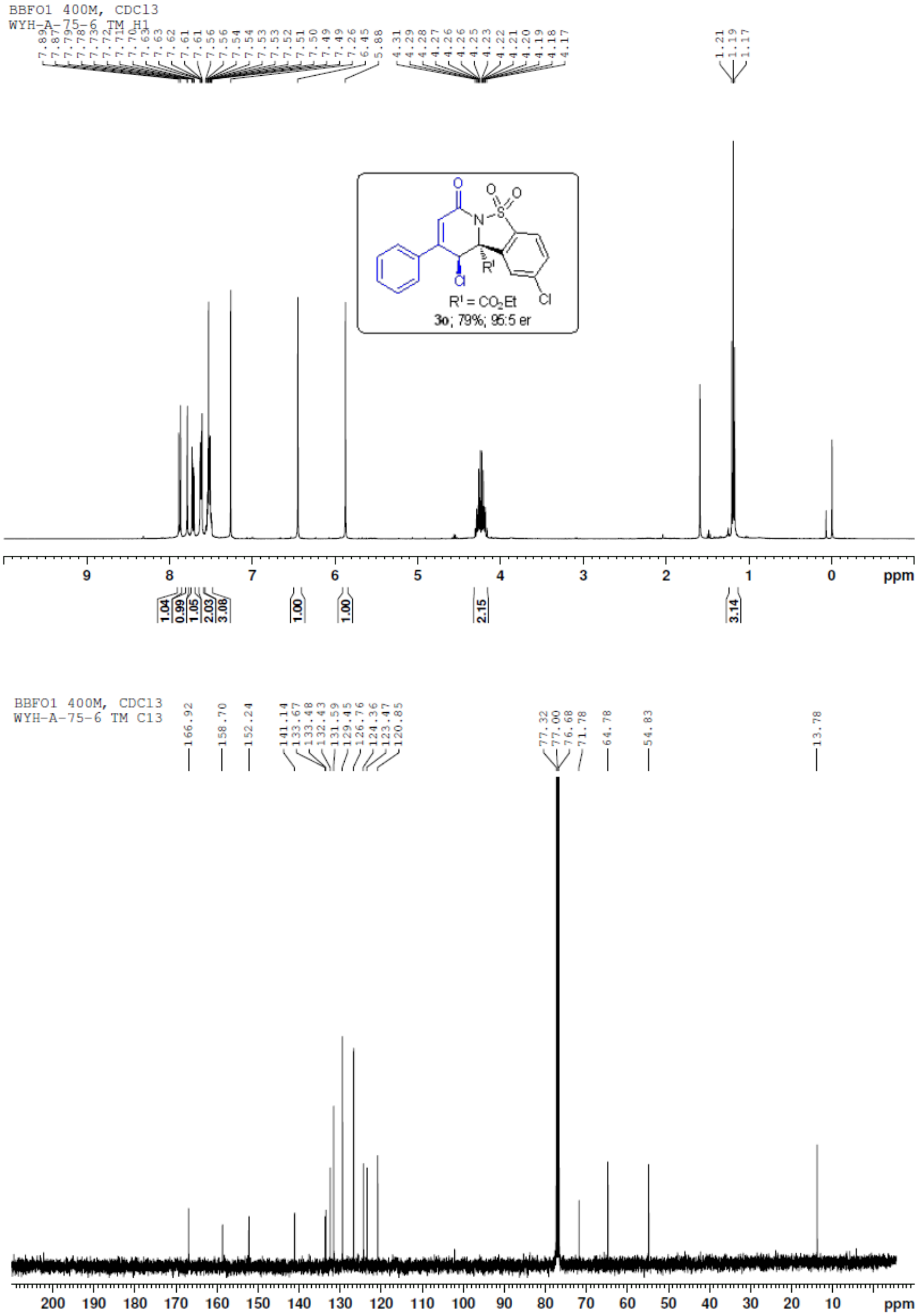

Supplementary figure 27. ^{1}H and ^{13}C NMR spectra for product **3o**

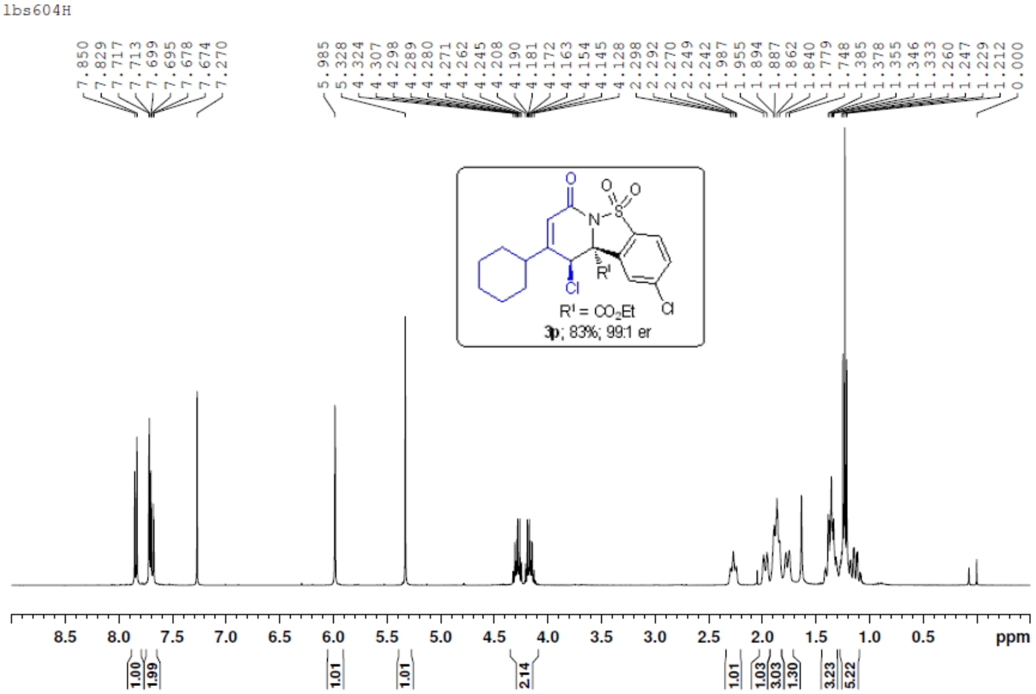

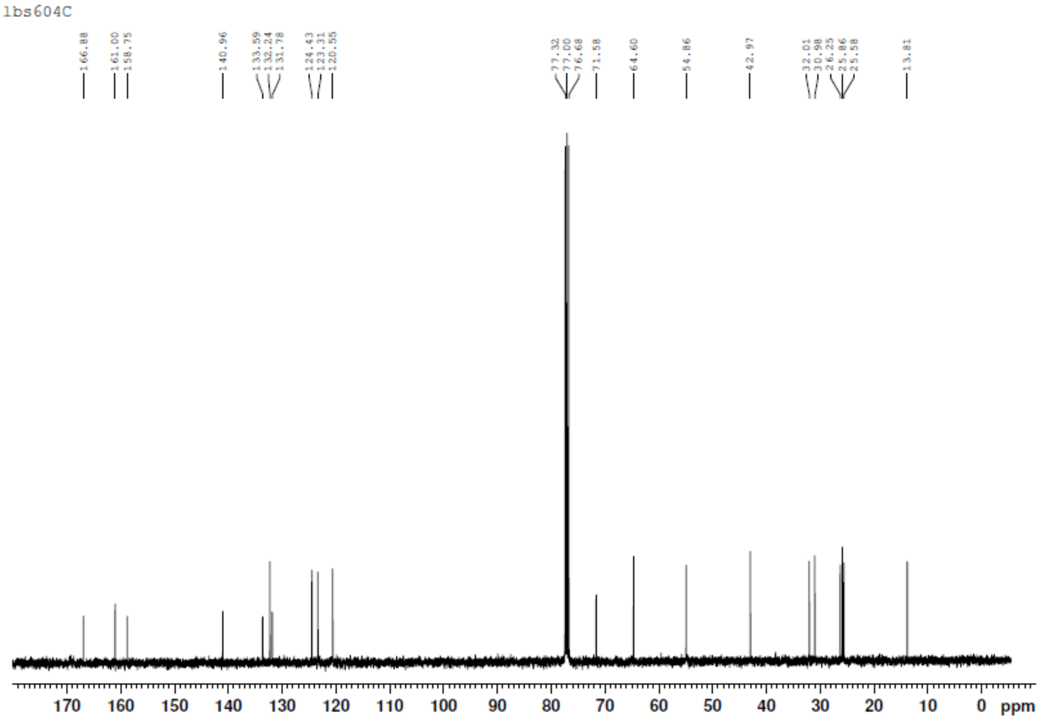

Supplementary figure 28. ^{1}H and ^{13}C NMR spectra for product **3p**

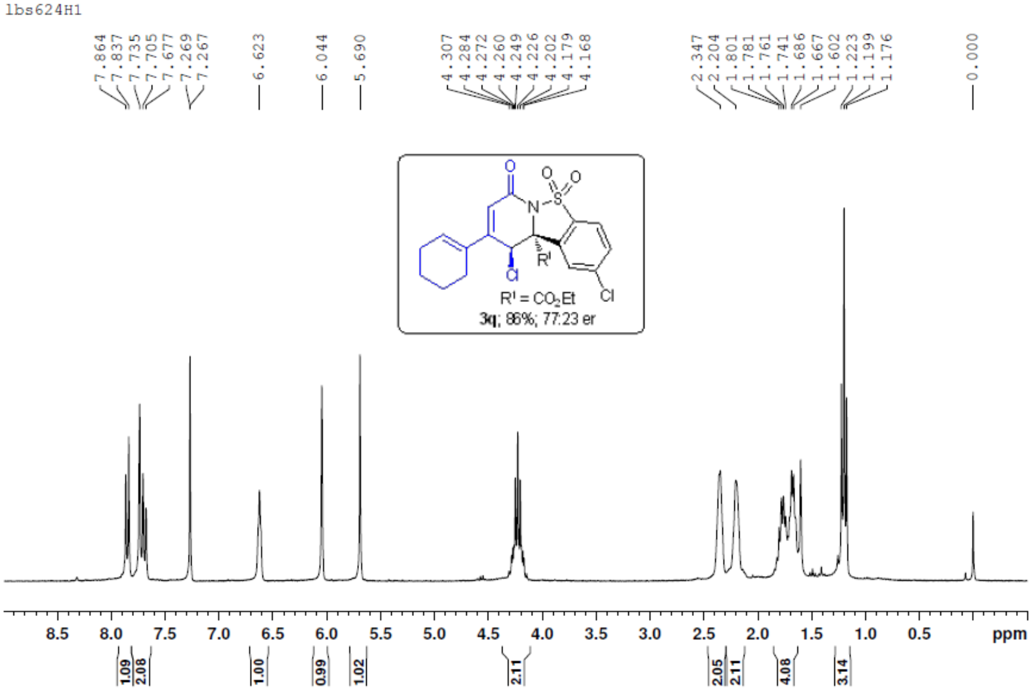

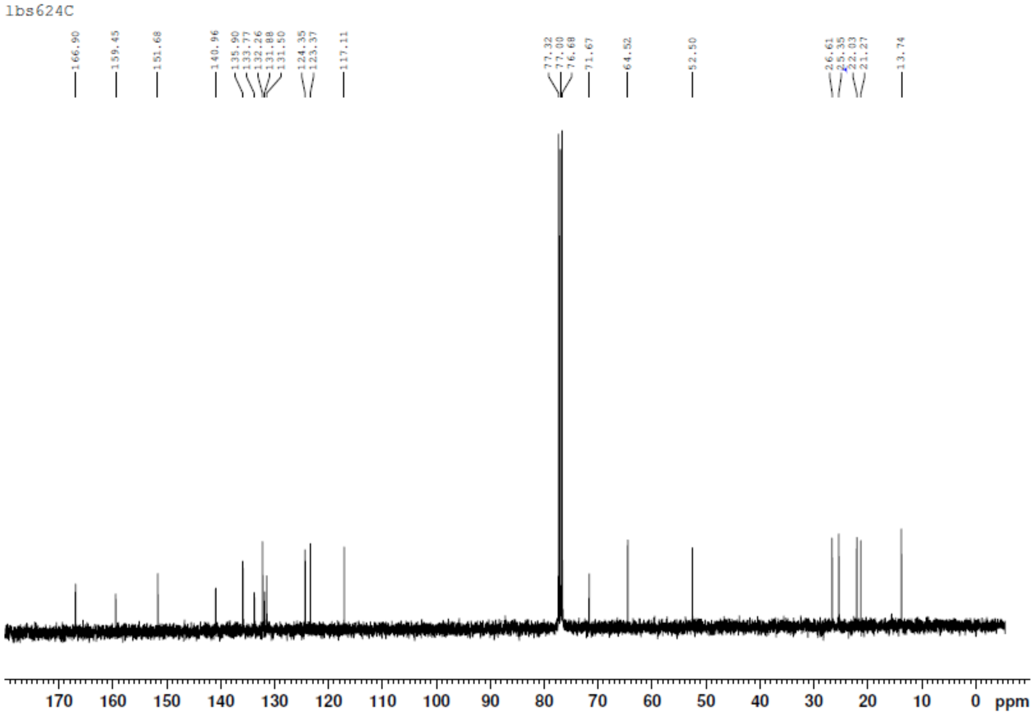

Supplementary figure 29. ^{1}H and ^{13}C NMR spectra for product **3q**

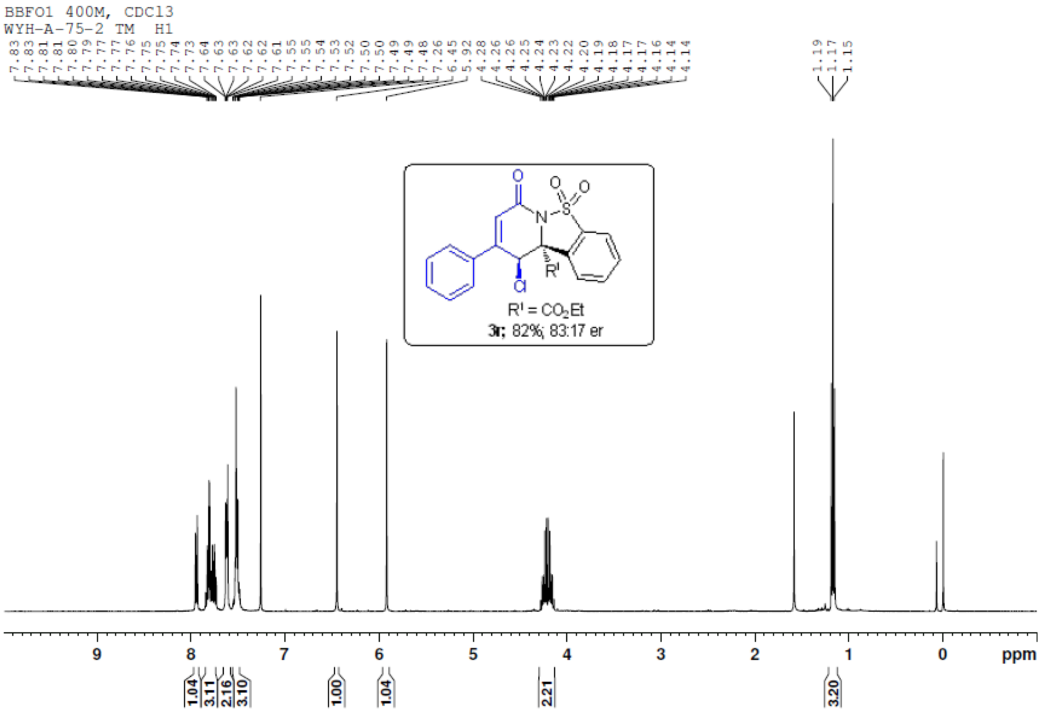

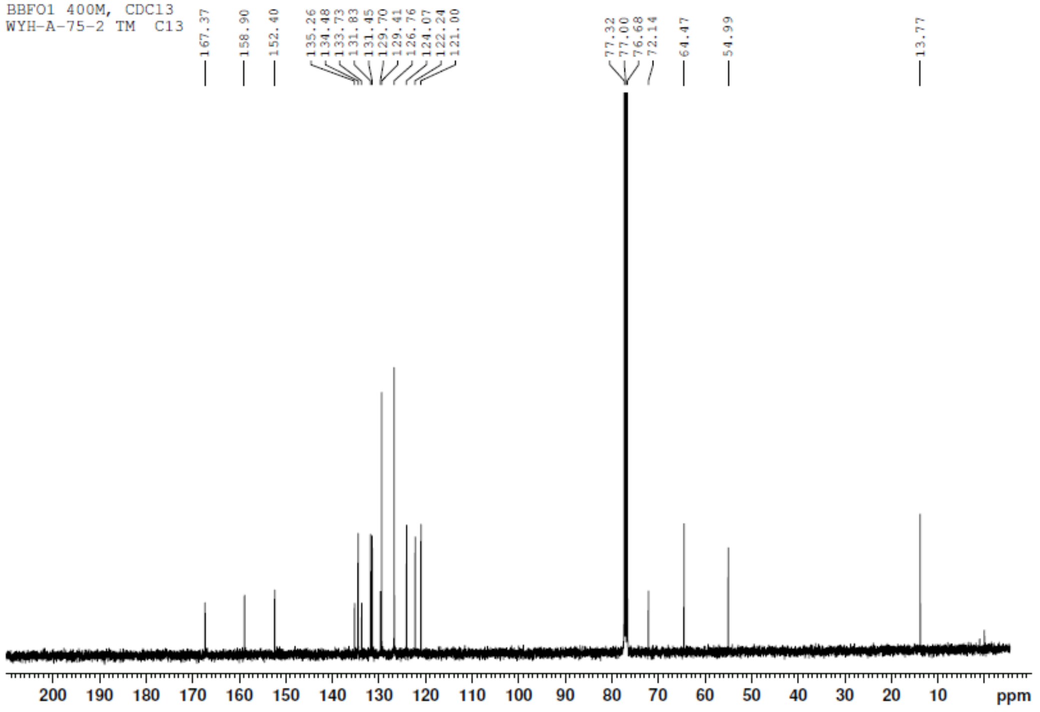

Supplementary figure 30. ^{1}H and ^{13}C NMR spectra for product **3r**

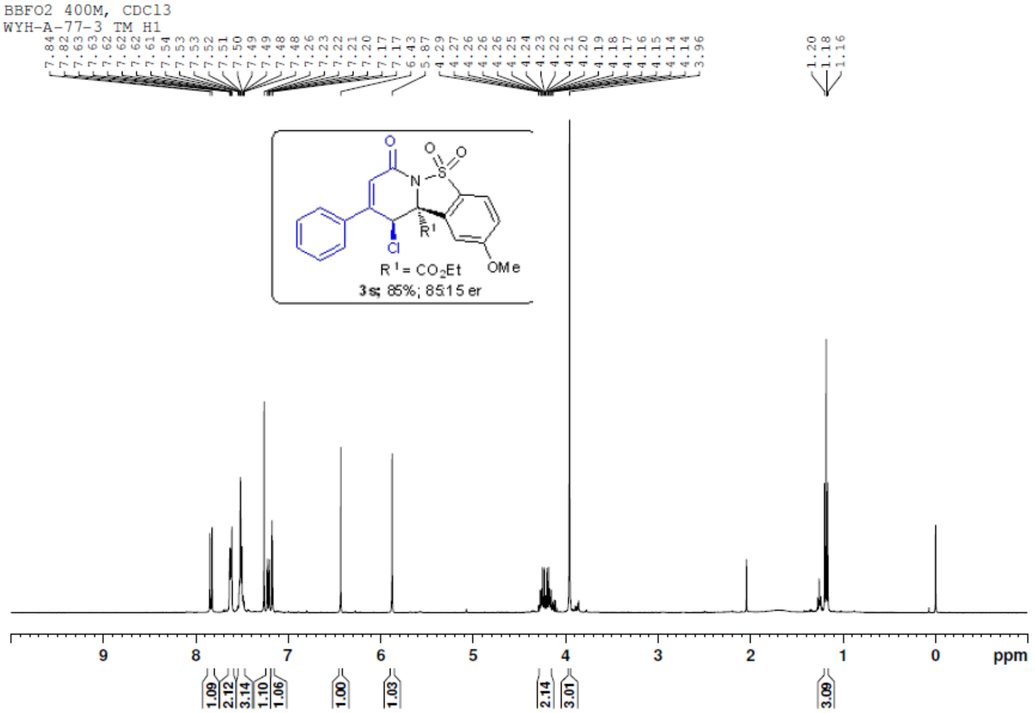

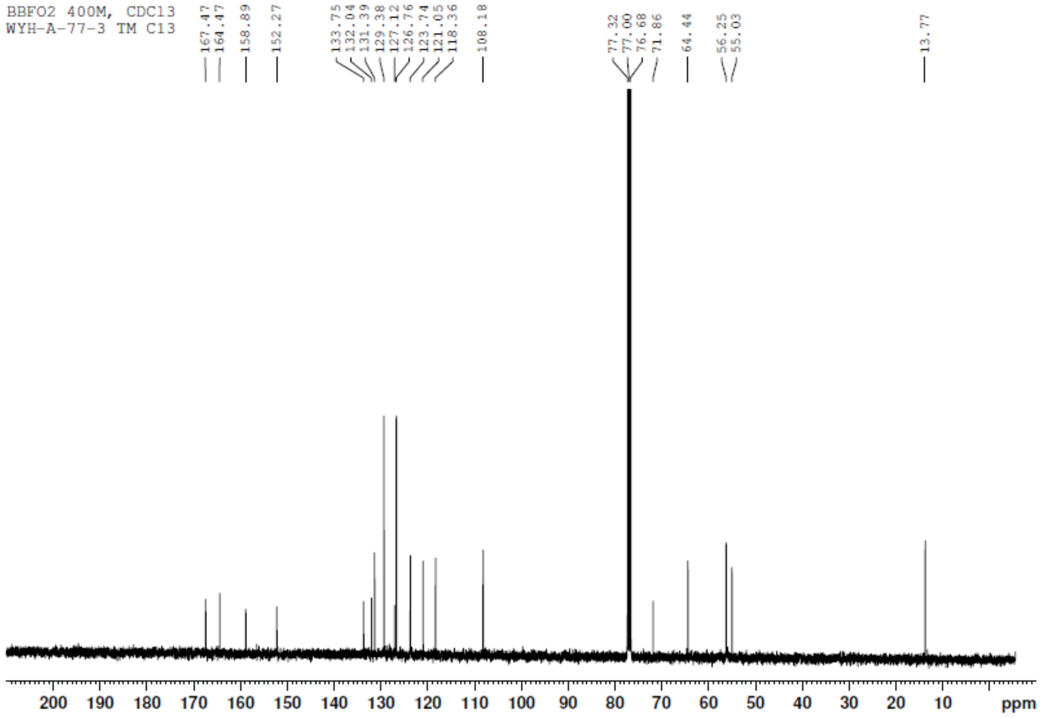

Supplementary figure 31. ^{1}H and ^{13}C NMR spectra for product **3s**

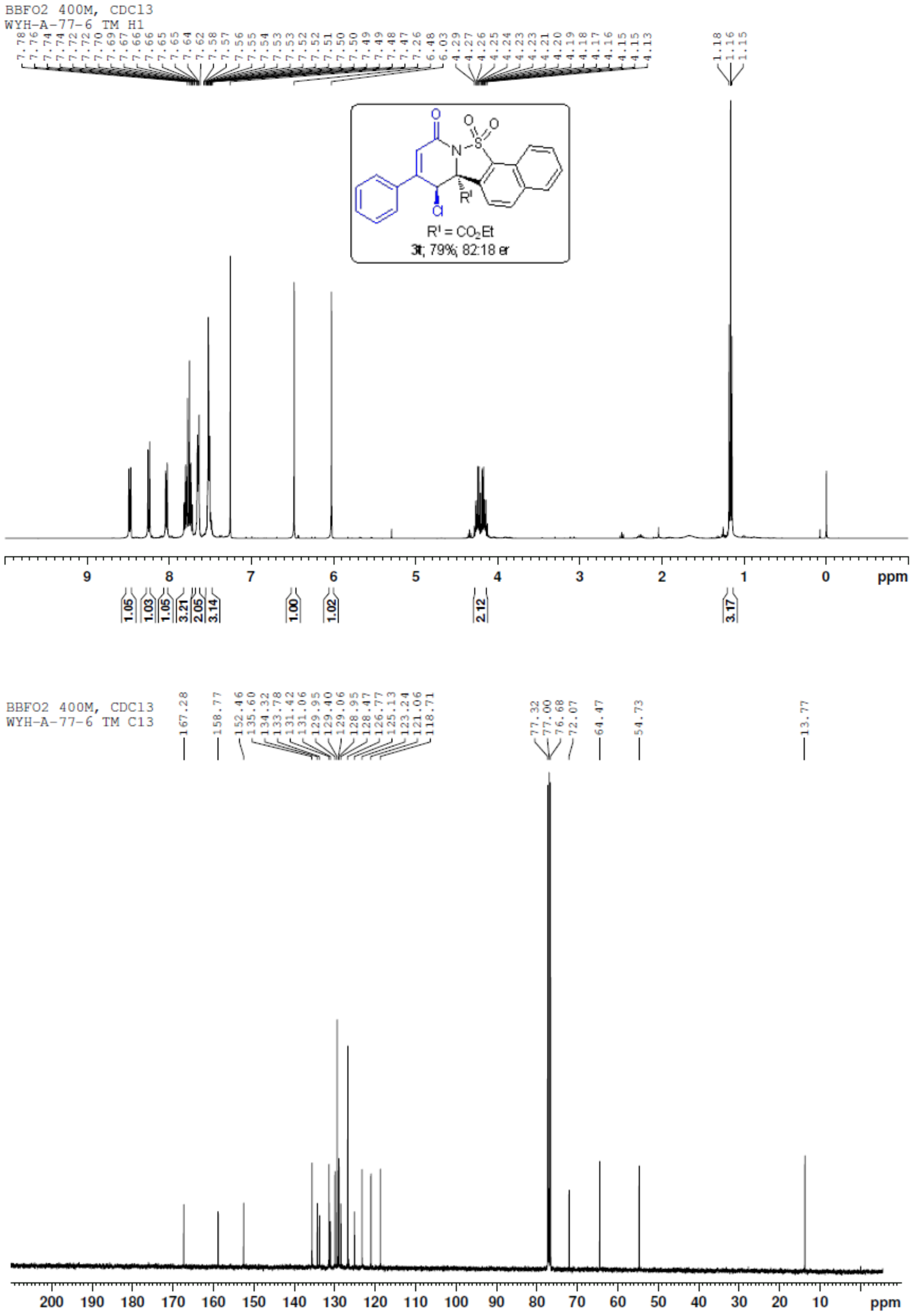

Supplementary figure 32. ^{1}H and ^{13}C NMR spectra for product **3t**

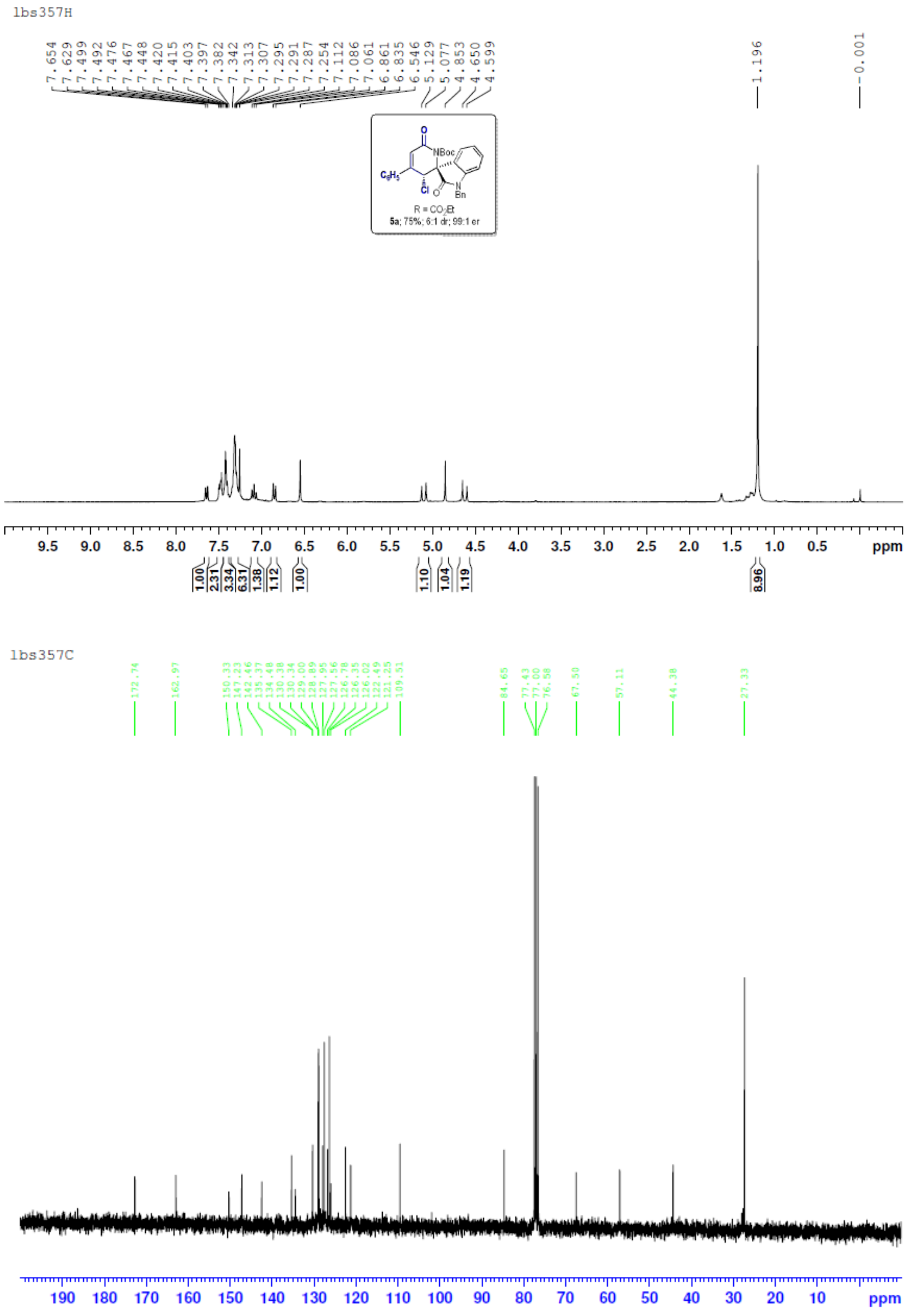

Supplementary figure 33. ^{1}H and ^{13}C NMR spectra for product **5a**

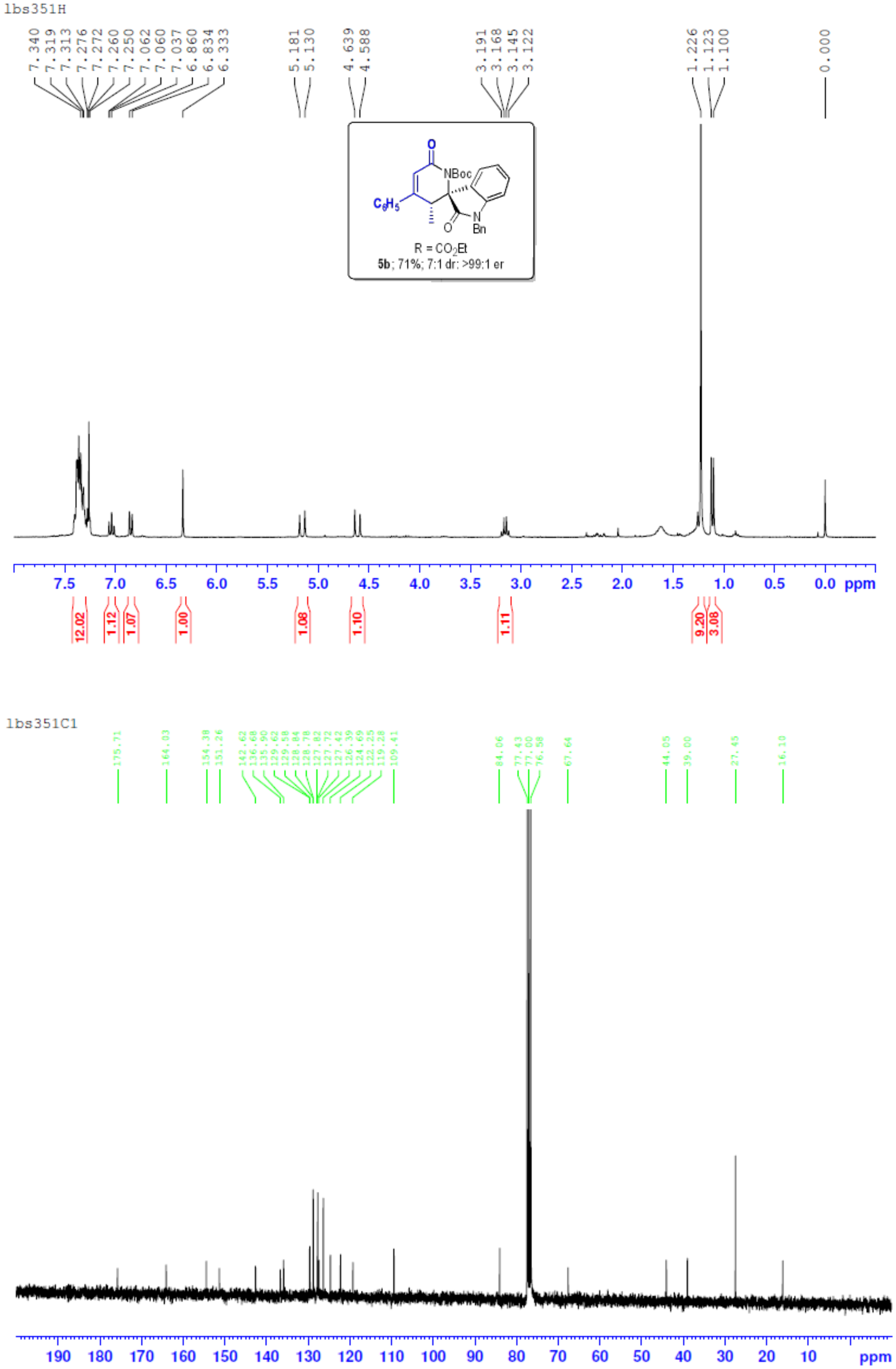

Supplementary figure 34. ^{1}H and ^{13}C NMR spectra for product **5b**

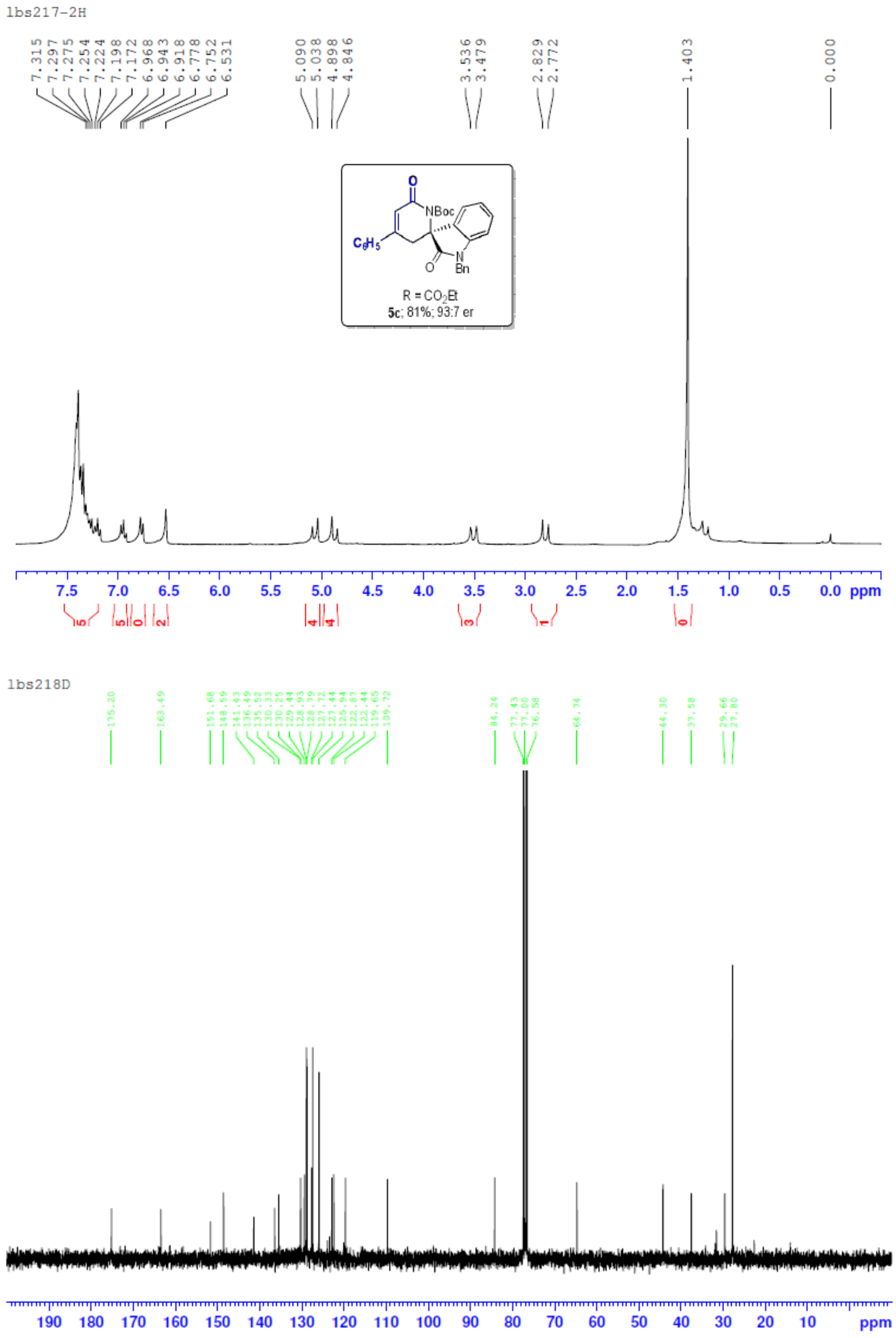

Supplementary figure 35. ^{1}H and ^{13}C NMR spectra for product **5c**

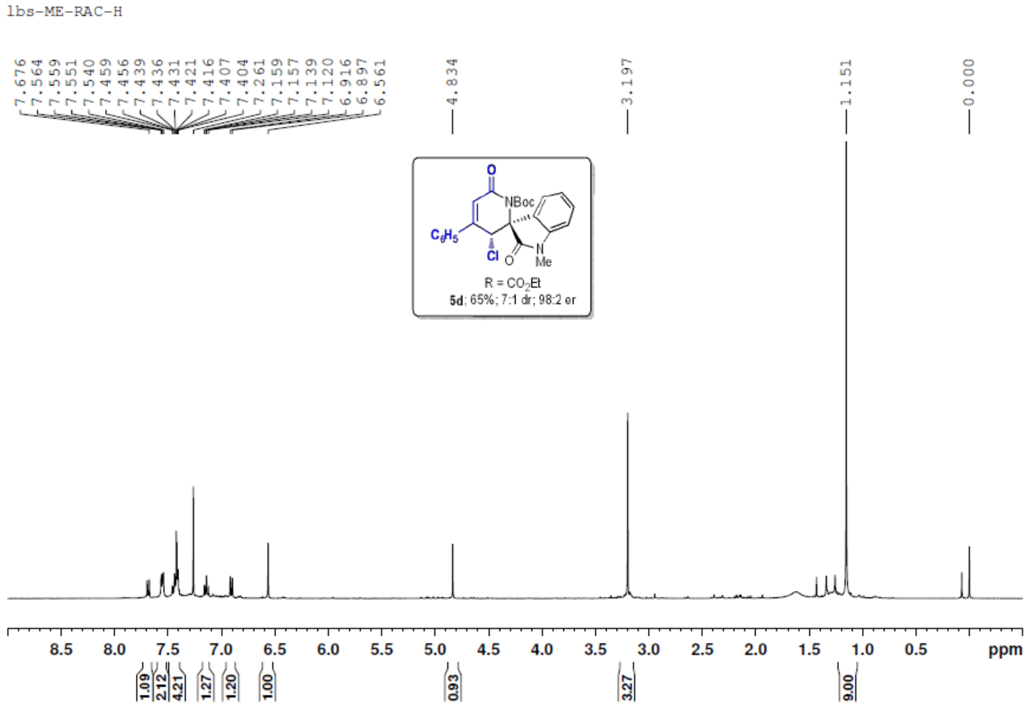

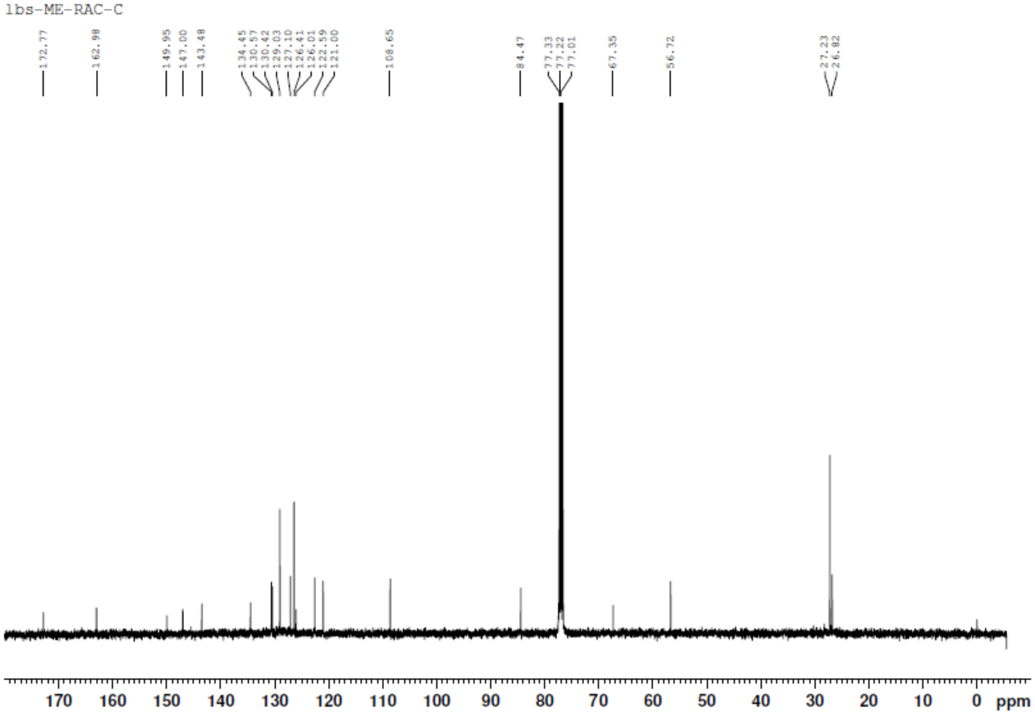

Supplementary figure 36. ^{1}H and ^{13}C NMR spectra for product **5d**

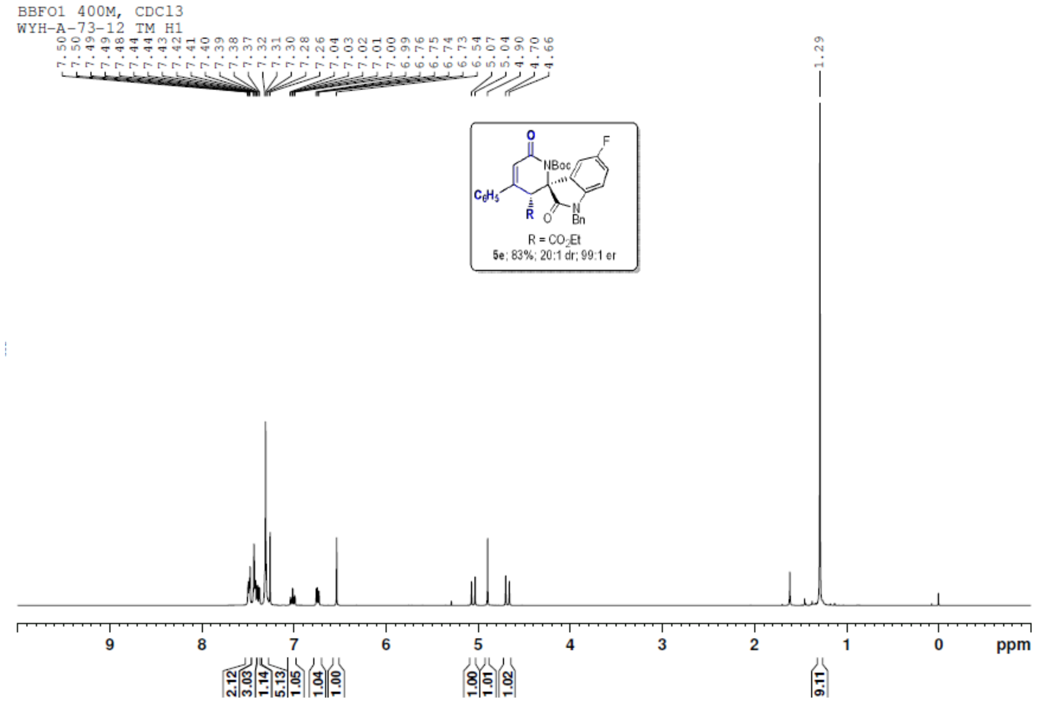

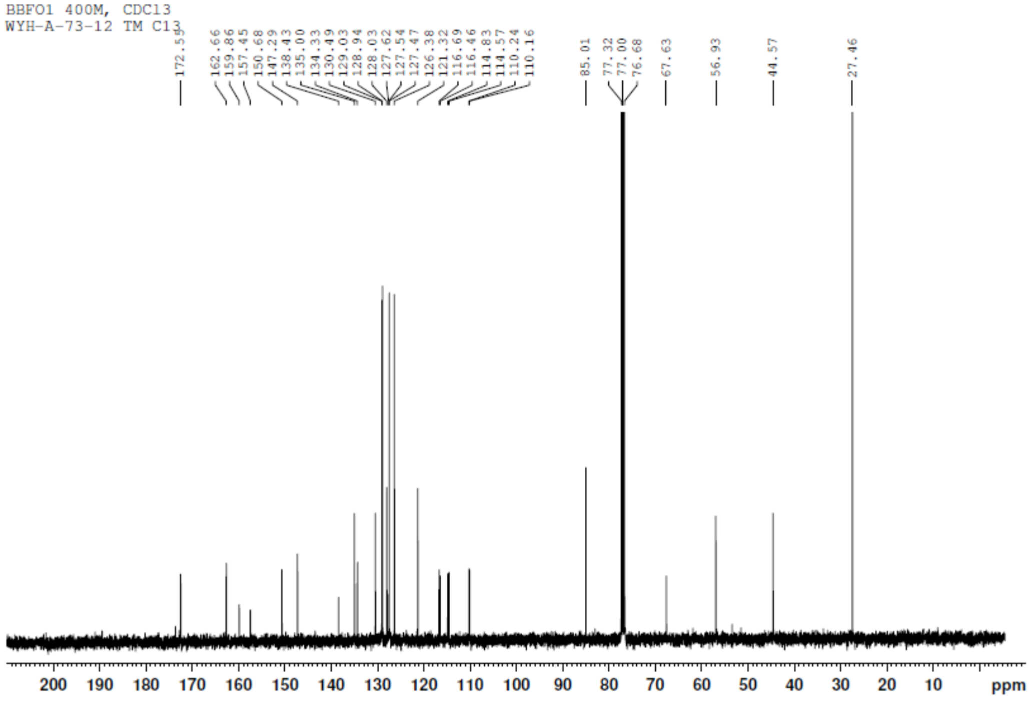

Supplementary figure 37. ^{1}H and ^{13}C NMR spectra for product **5e**

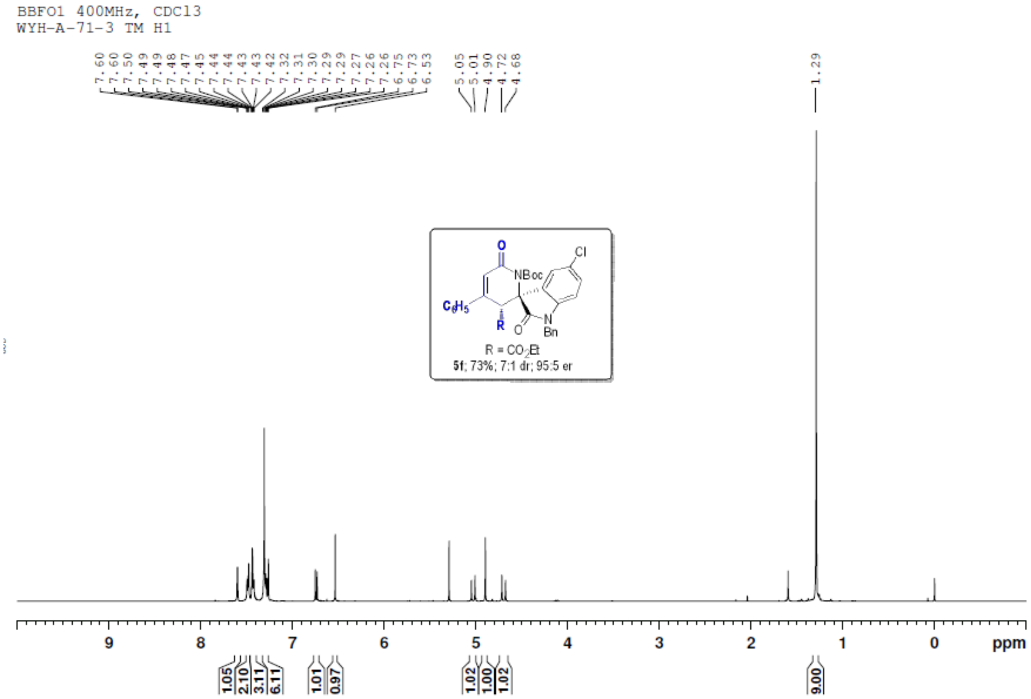

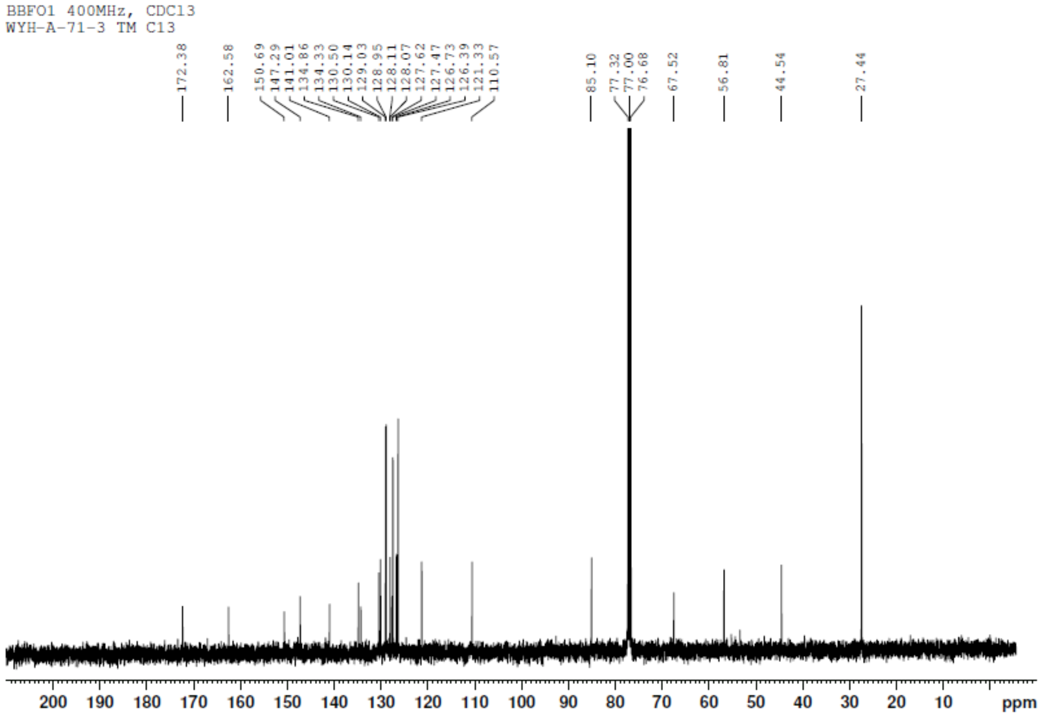

Supplementary figure 38. ^{1}H and ^{13}C NMR spectra for product **5f**

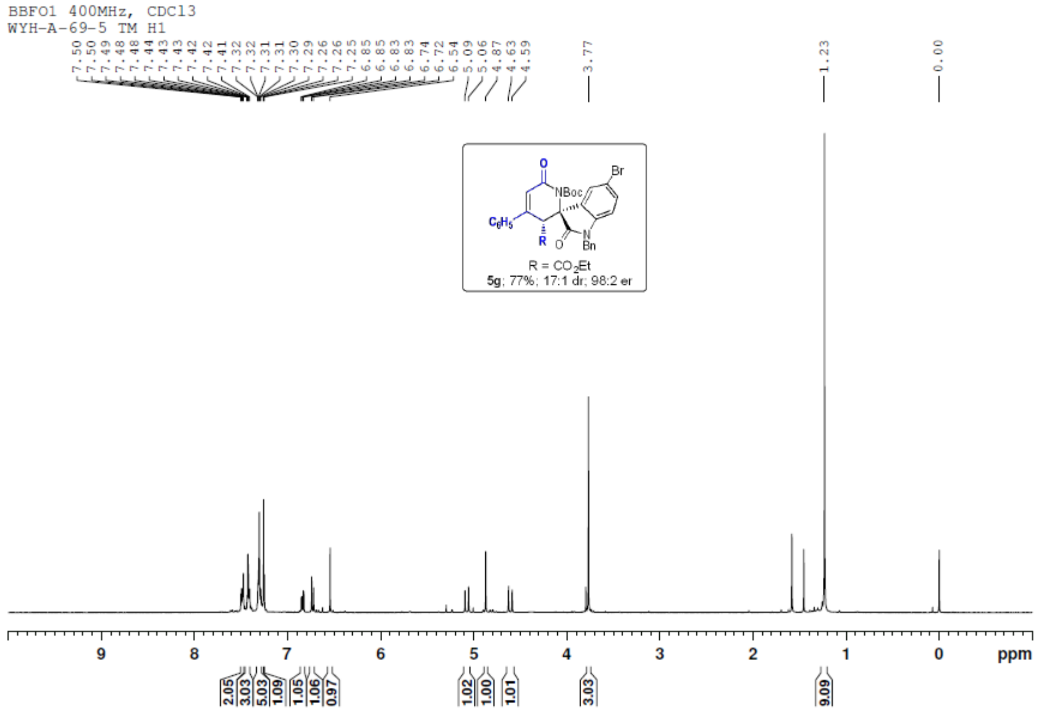

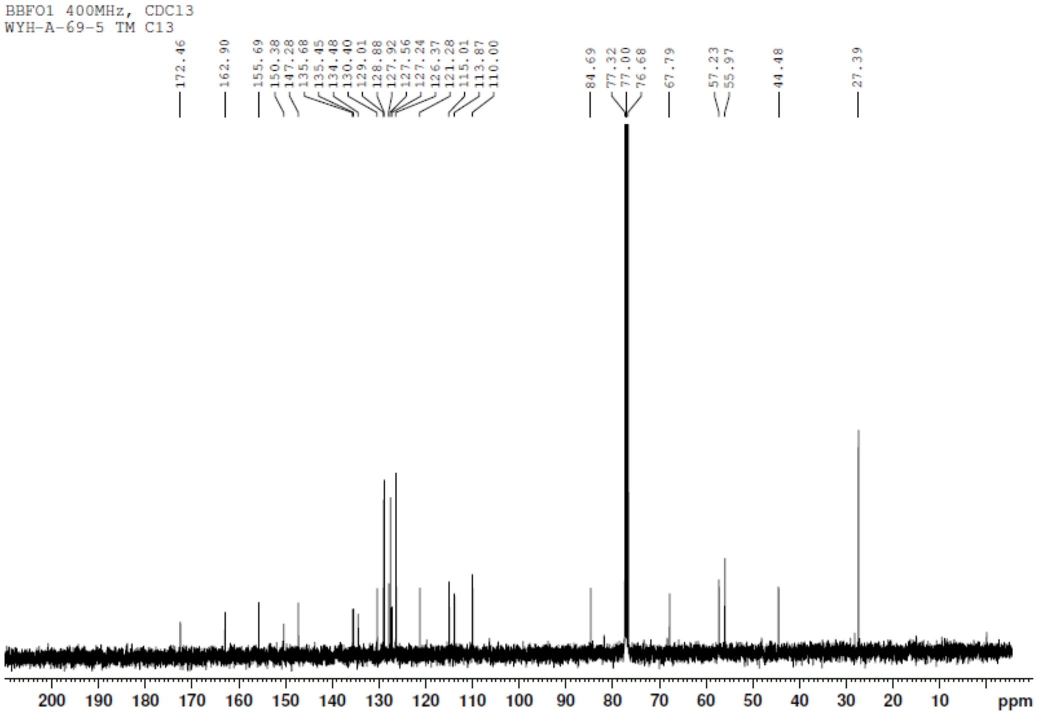

Supplementary figure 39. ^{1}H and ^{13}C NMR spectra for product **5g**

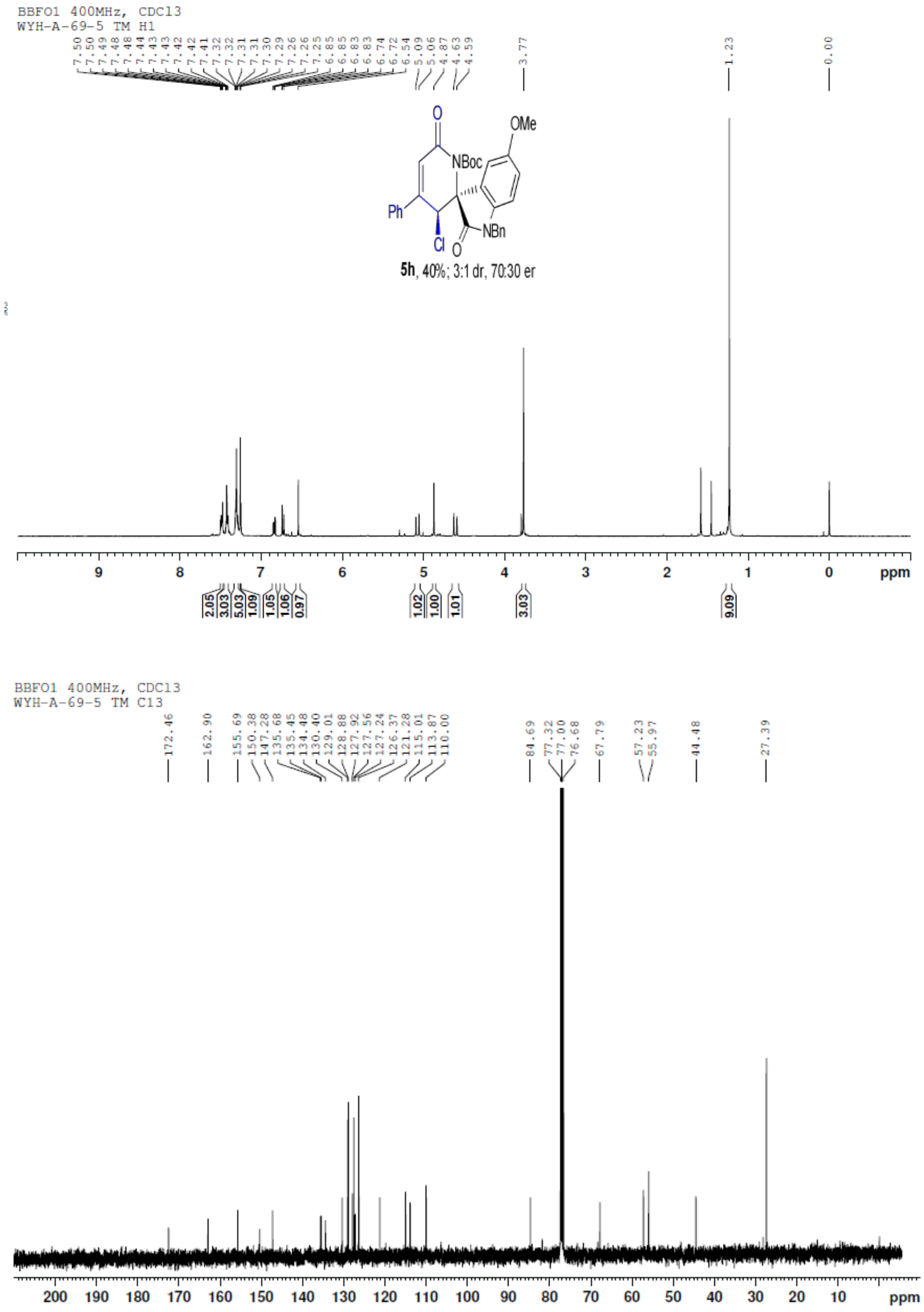

Supplementary figure 40. ^{1}H and ^{13}C NMR spectra for product **5h**

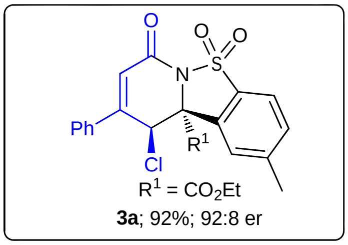

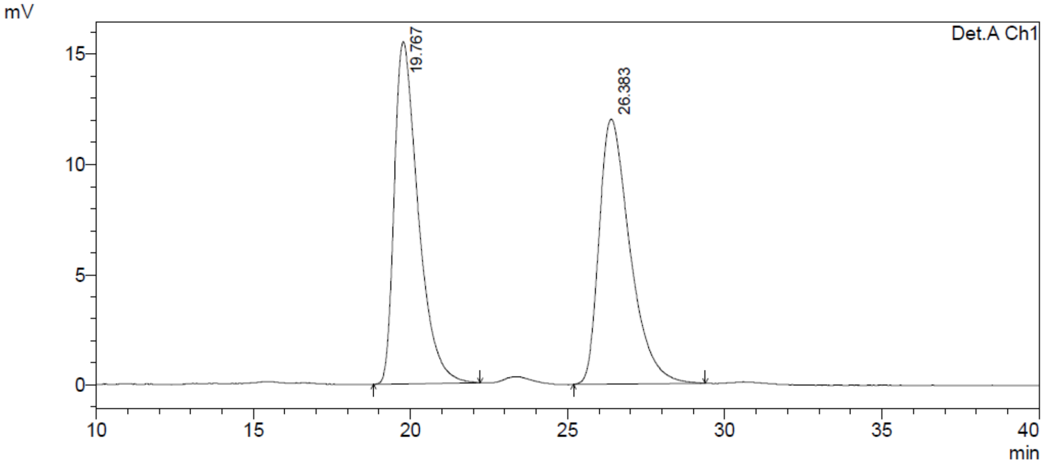

UV Detector Ch1 254nm

Peak#	Ret. Time	Area	Height	Area %	Height %
1	19.767	823196	15513	50.006	56.343
2	26.383	822987	12020	49.994	43.657
Total		1646182	27533	100.000	100.000

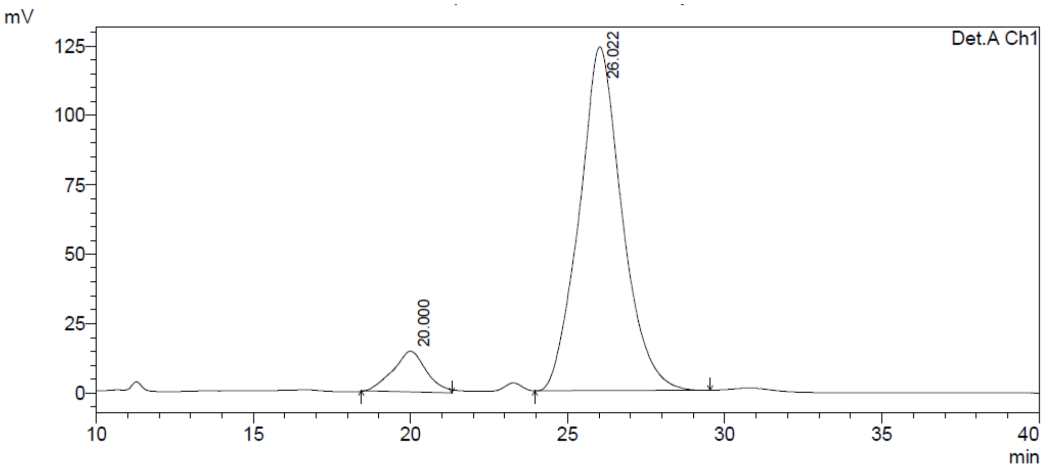

Peak#	Ret. Time	Area	Height	Area %	Height %
1	20.000	1088217	14673	8.494	10.594
2	26.022	11722900	123826	91.506	89.406
Total		12811117	138499	100.000	100.000

Supplementary figure 41. HPLC spectra for product **3a**

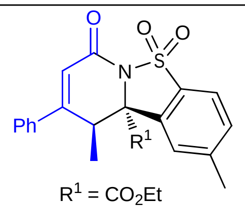

R^1 = CO_2Et
3b; 90%; 89:11 er

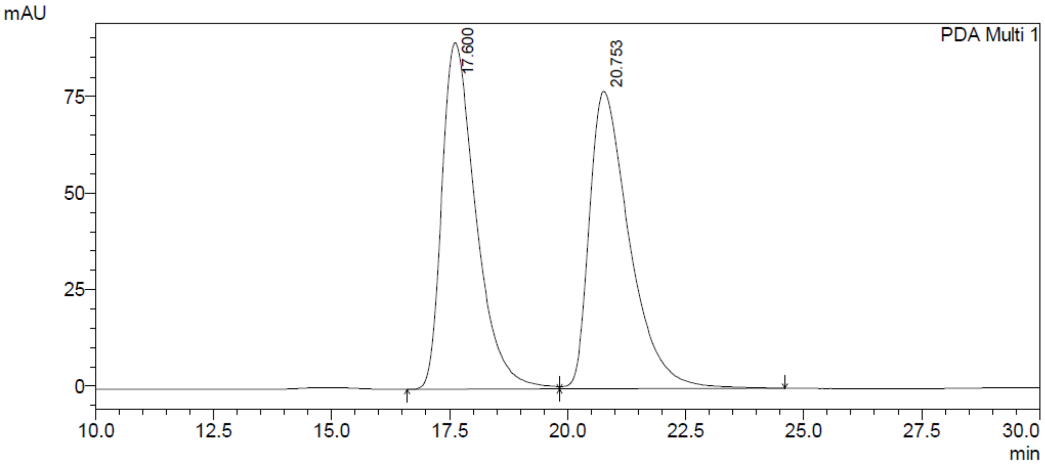

PDA Ch1 254nm 4nm

Peak#	Ret. Time	Area	Height	Area %	Height %
1	17.600	4505872	89569	49.734	53.750
2	20.753	4554129	77071	50.266	46.250
Total		9060000	166640	100.000	100.000

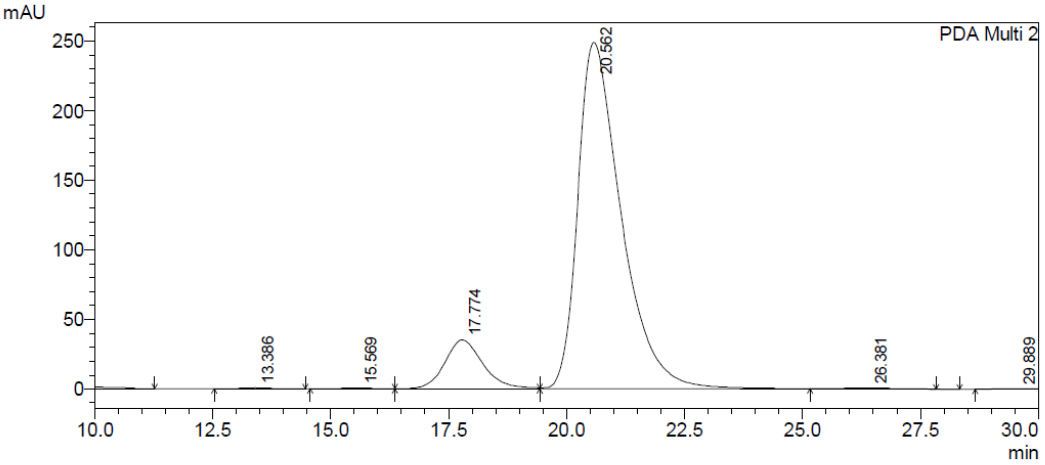

Peak#	Ret. Time	Area	Height	Area %	Height %
1	17.772	1260534	22377	11.069	12.349
2	20.562	10127899	158822	88.931	87.651
Total		11388433	181199	100.000	100.000

Supplementary figure 42. HPLC spectra for product **3b**

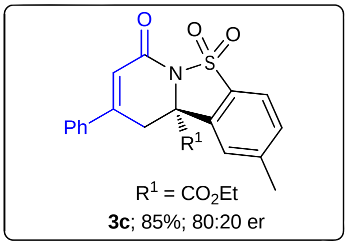

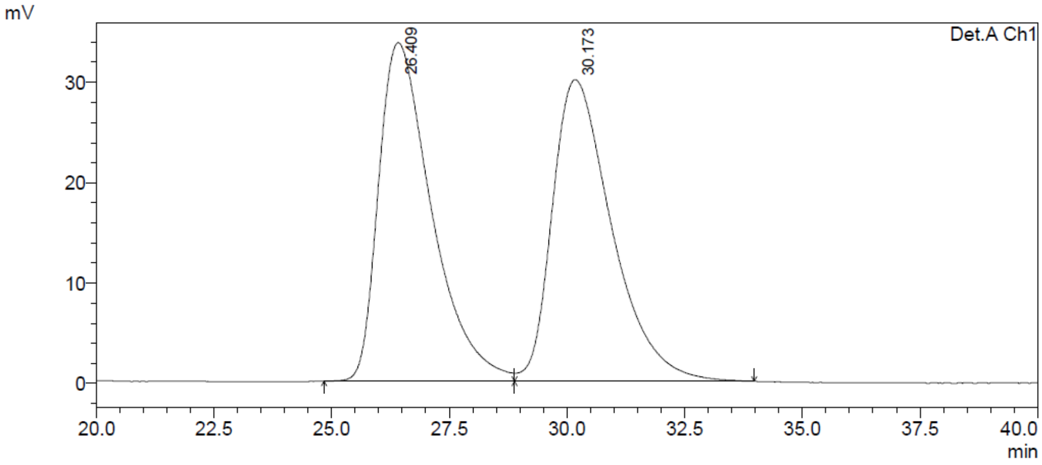

Peak#	Ret. Time	Area	Height	Area %	Height %
1	26.409	2651771	33744	49.988	52.887
2	30.173	2653059	30061	50.012	47.113
Total		5304830	63805	100.000	100.000

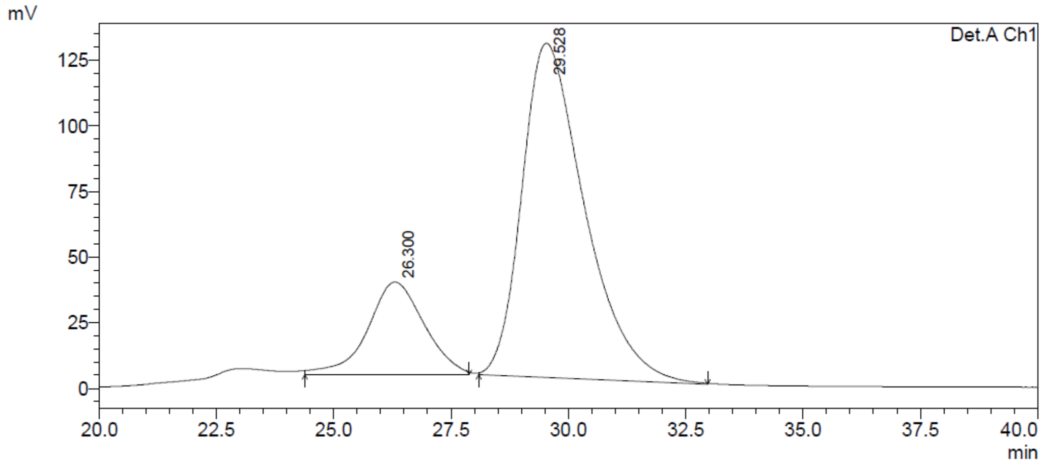

UV Detector Ch1 254nm

Peak#	Ret. Time	Area	Height	Area %	Height %
1	26.300	2937928	35164	19.816	21.643
2	29.528	11888171	127309	80.184	78.357
Total		14826099	162473	100.000	100.000

Supplementary figure 43. HPLC spectra for product **3c**

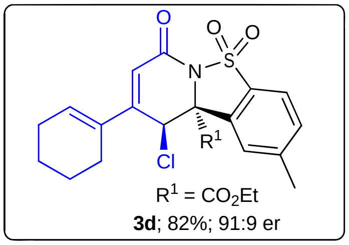

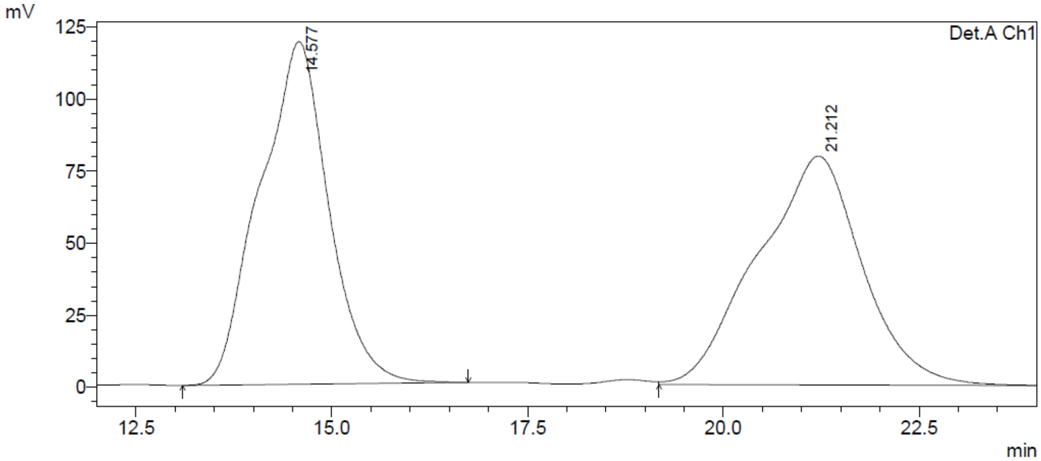

UV Detector Ch1 254nm

Peak#	Ret. Time	Area	Height	Area %	Height %
1	14.577	7272835	118892	49.660	59.961
2	21.212	7372286	79390	50.340	40.039
Total		14645121	198282	100.000	100.000

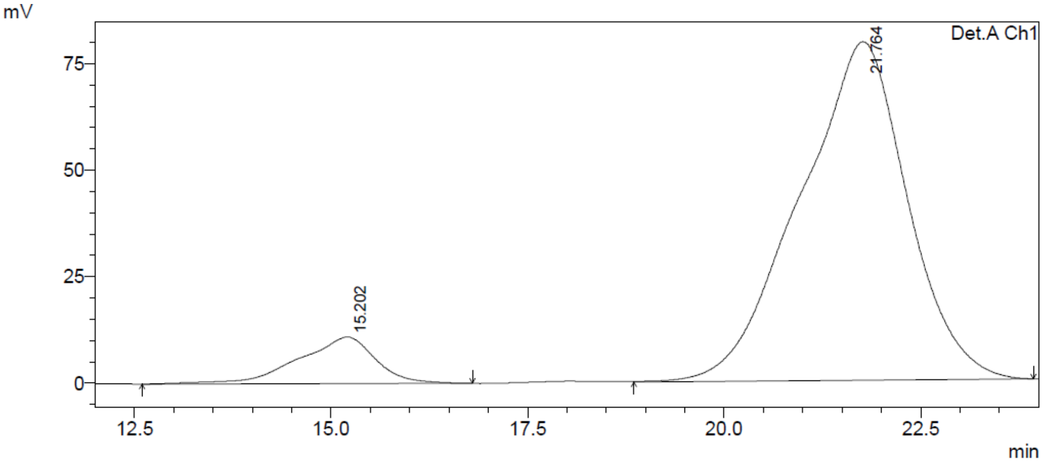

UV Detector Ch1 254nm

Peak#	Ret. Time	Area	Height	Area %	Height %
1	15.202	748215	10925	9.047	12.108
2	21.764	7522338	79306	90.953	87.892
Total		8270554	90232	100.000	100.000

Supplementary figure 44. HPLC spectra for product **3d**

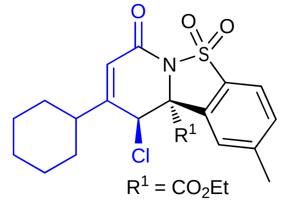

$R^1 = CO_2Et$

3e; 84%; 96:4 er

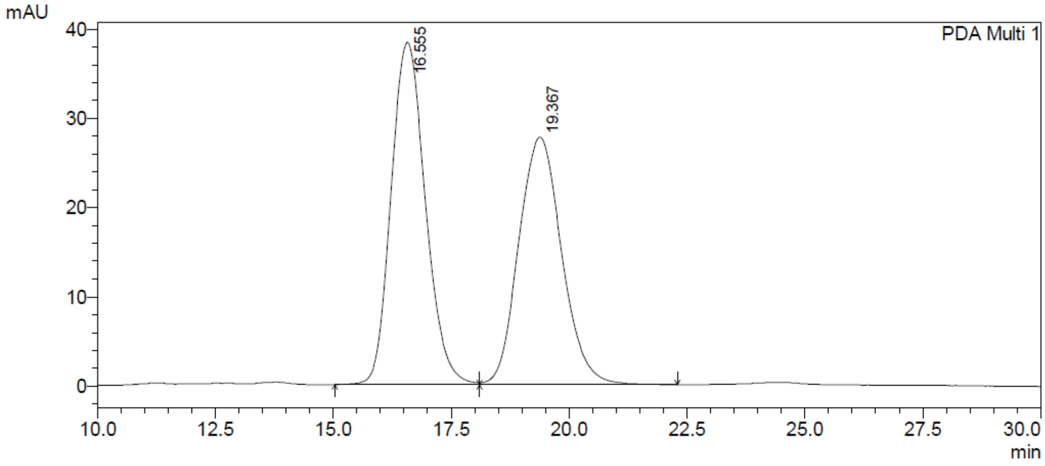

Peak#	Ret. Time	Area	Height	Area %	Height %
1	16.555	1944543	38384	52.462	58.060
2	19.367	1762037	27726	47.538	41.940
Total		3706580	66110	100.000	100.000

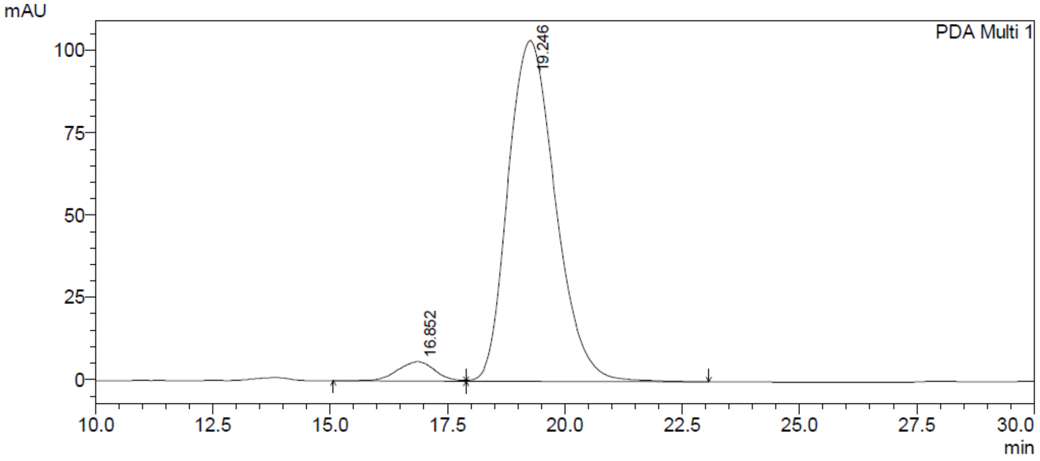

Peak#	Ret. Time	Area	Height	Area %	Height %
1	16.852	334319	5912	4.394	5.402
2	19.246	7273394	103539	95.606	94.598
Total		7607713	109451	100.000	100.000

Supplementary figure 45. HPLC spectra for product **3e**

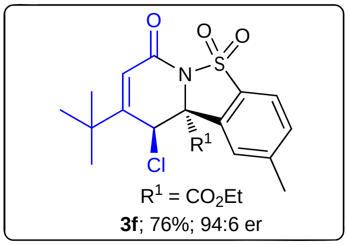

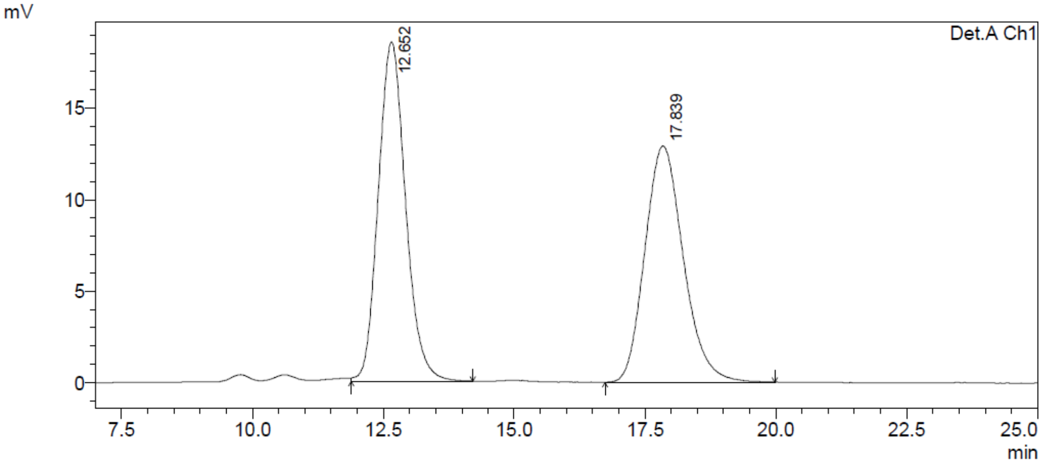

UV Detector Ch1 254nm

Peak#	Ret. Time	Area	Height	Area %	Height %
1	12.652	674001	18569	50.477	58.960
2	17.839	661269	12925	49.523	41.040
Total		1335270	31494	100.000	100.000

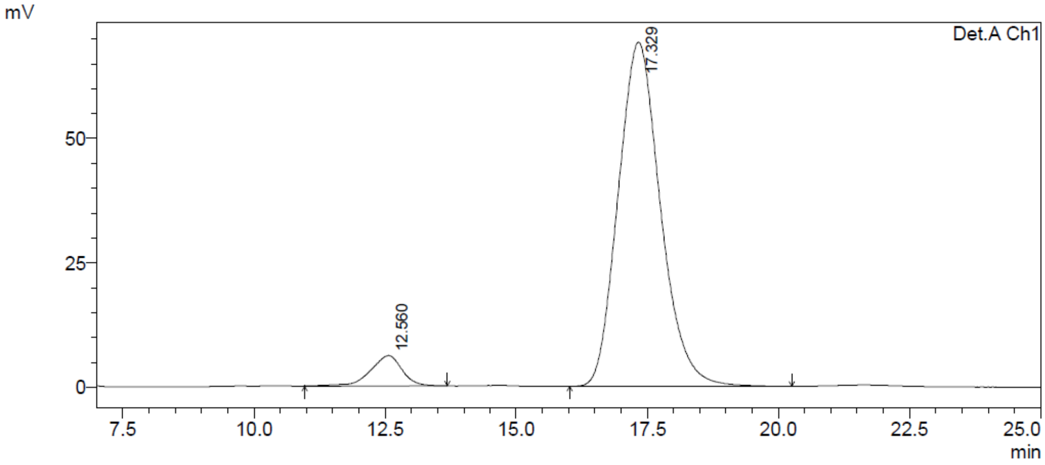

UV Detector Ch1 254nm

Peak#	Ret. Time	Area	Height	Area %	Height %
1	12.560	260198	6139	6.285	8.156
2	17.329	3879475	69129	93.715	91.844
Total		4139673	75268	100.000	100.000

Supplementary figure 46. HPLC spectra for product **3f**

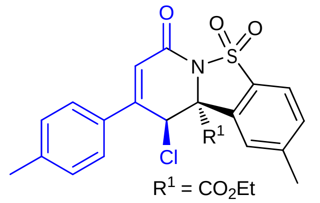

$R^1 = CO_2Et$
3g; 90%; 91:9 er

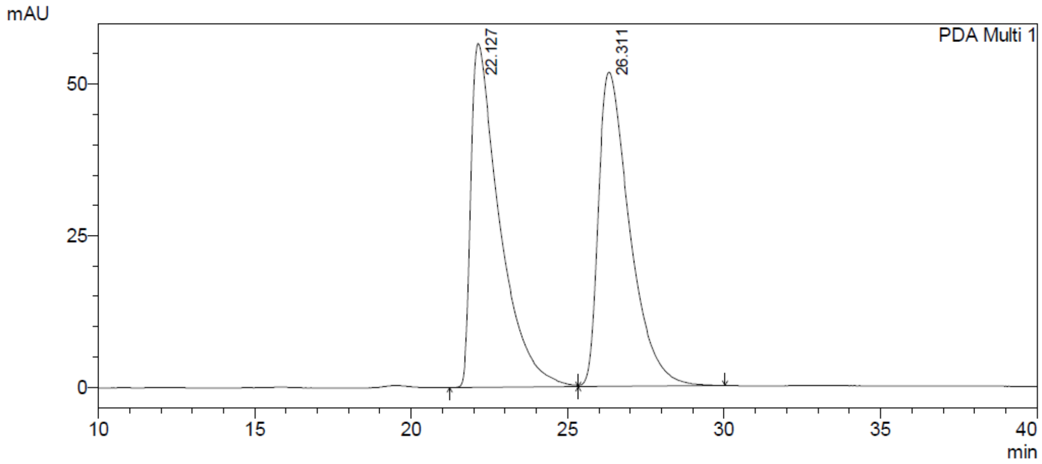

PDA Ch1 254nm 4nm

Peak#	Ret. Time	Area	Height	Area %	Height %
1	22.127	3616673	56680	50.094	52.247
2	26.311	3603121	51804	49.906	47.753
Total		7219794	108484	100.000	100.000

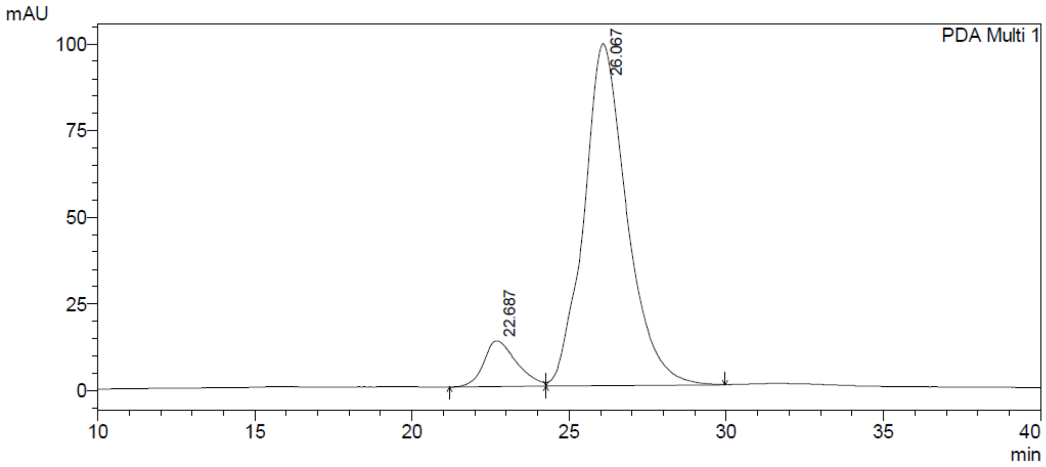

Peak#	Ret. Time	Area	Height	Area %	Height %
1	22.687	963634	13215	9.320	11.808
2	26.067	9375384	98699	90.680	88.192
Total		10339018	111913	100.000	100.000

Supplementary figure 47. HPLC spectra for product **3g**

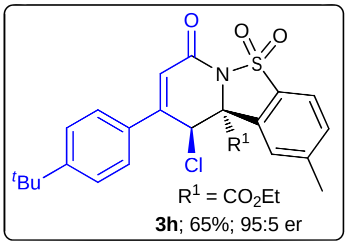

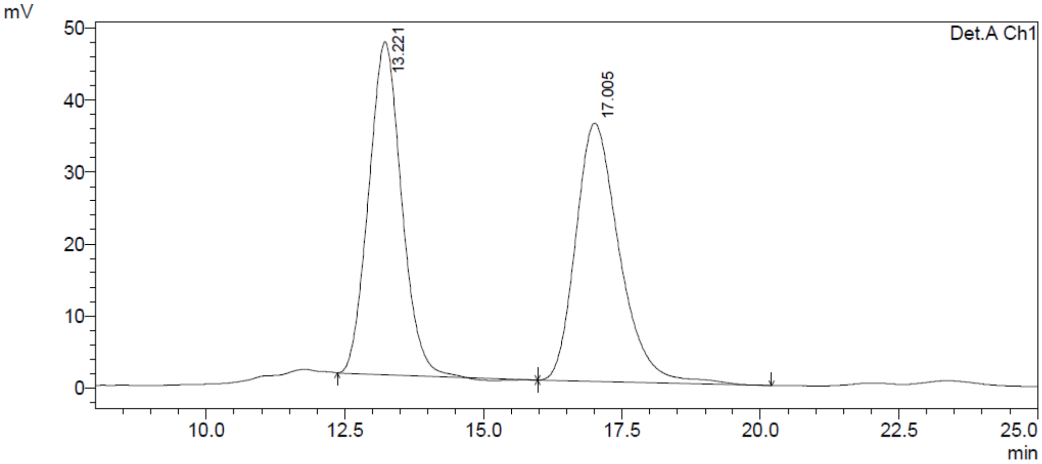

UV Detector Ch1 254nm

Peak#	Ret. Time	Area	Height	Area %	Height %
1	13.221	1896296	46195	49.117	56.315
2	17.005	1964512	35835	50.883	43.685
Total		3860808	82031	100.000	100.000

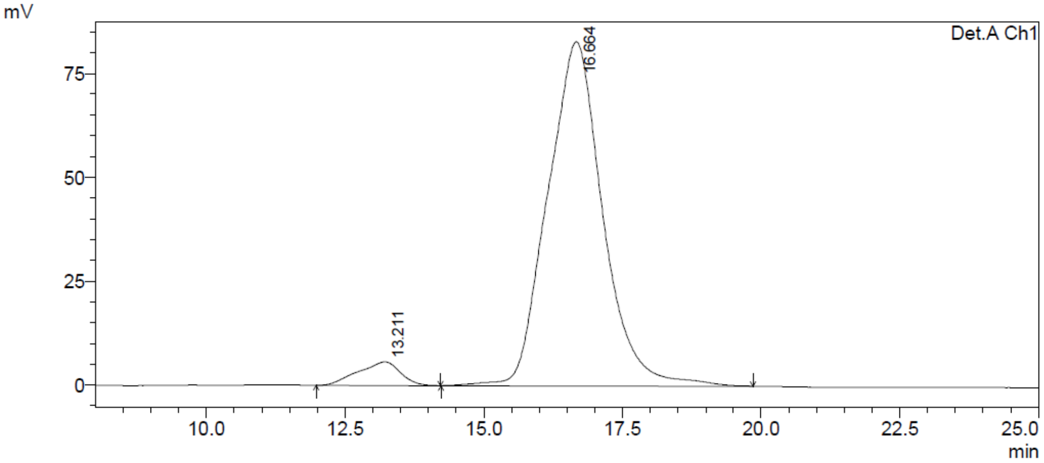

Peak#	Ret. Time	Area	Height	Area %	Height %
1	13.211	302867	5696	5.022	6.437
2	16.664	5727366	82799	94.978	93.563
Total		6030233	88495	100.000	100.000

Supplementary figure 48. HPLC spectra for product **3h**

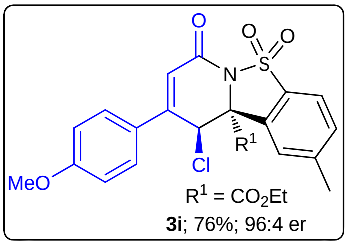

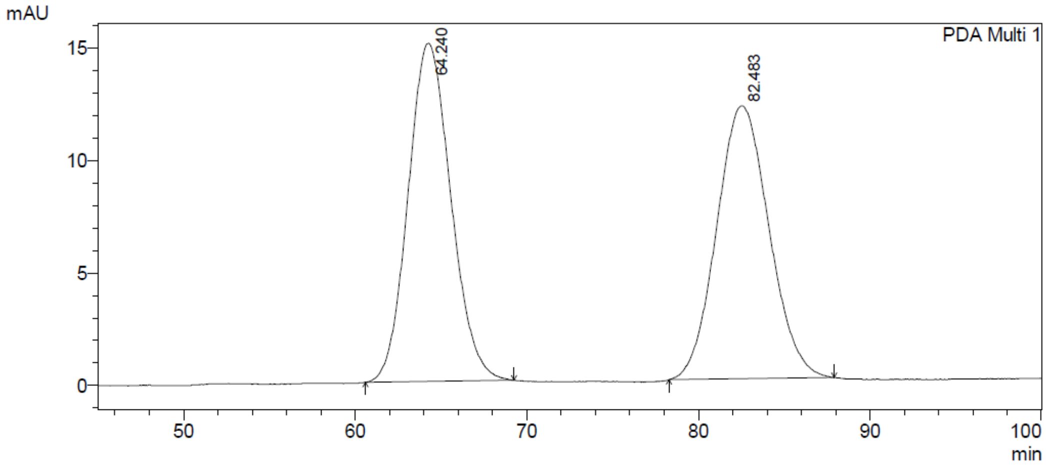

PDA Ch1 254nm 4nm

Peak#	Ret. Time	Area	Height	Area %	Height %
1	64.240	2612343	15029	50.366	55.325
2	82.483	2574406	12136	49.634	44.675
Total		5186749	27165	100.000	100.000

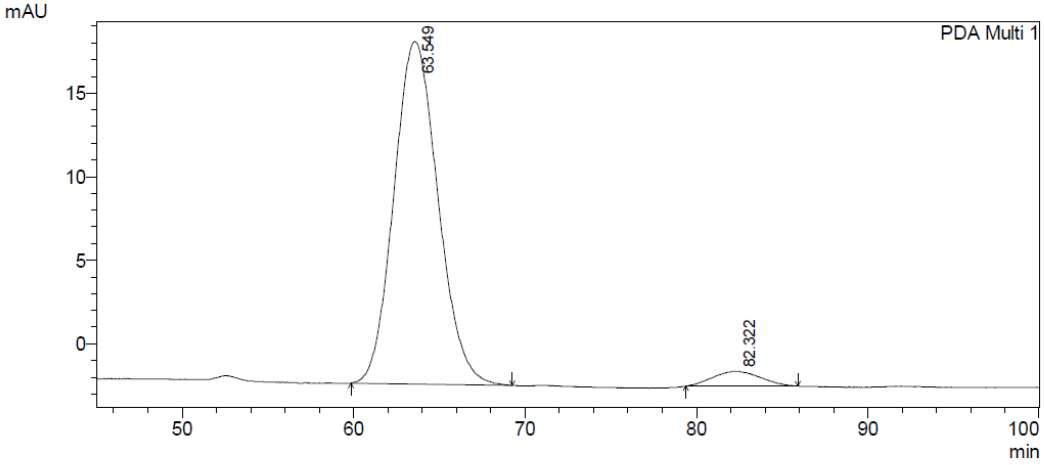

PDA Ch1 254nm 4nm

Peak#	Ret. Time	Area	Height	Area %	Height %
1	63.549	3678904	20501	95.529	95.858
2	82.322	172190	886	4.471	4.142
Total		3851094	21387	100.000	100.000

Supplementary figure 49. HPLC spectra for product **3i**

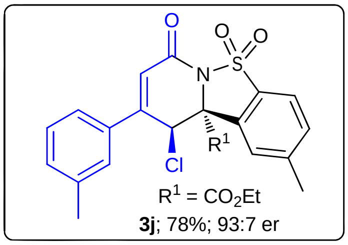

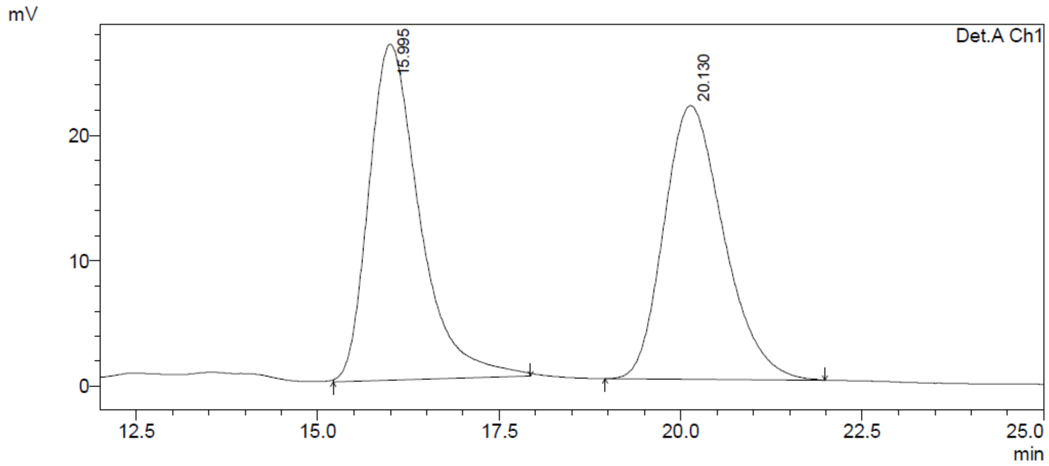

UV Detector Ch1 254nm

Peak#	Ret. Time	Area	Height	Area %	Height %
1	15.995	1307045	26778	51.085	55.107
2	20.130	1251537	21815	48.915	44.893
Total		2558582	48593	100.000	100.000

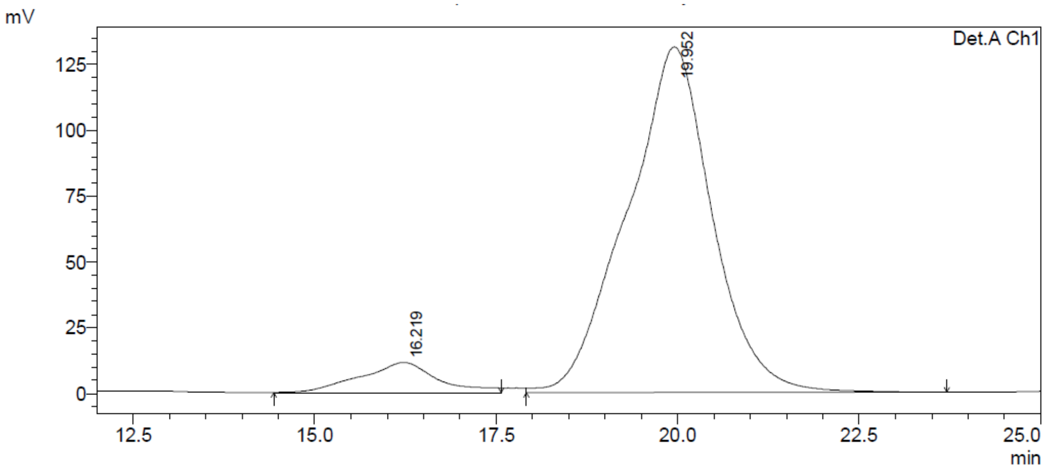

Peak#	Ret. Time	Area	Height	Area %	Height %
1	16.219	831221	11394	7.214	7.989
2	19.952	10691435	131226	92.786	92.011
Total		11522656	142620	100.000	100.000

Supplementary figure 50. HPLC spectra for product **3j**

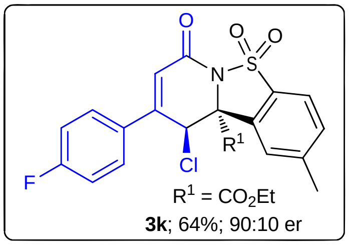

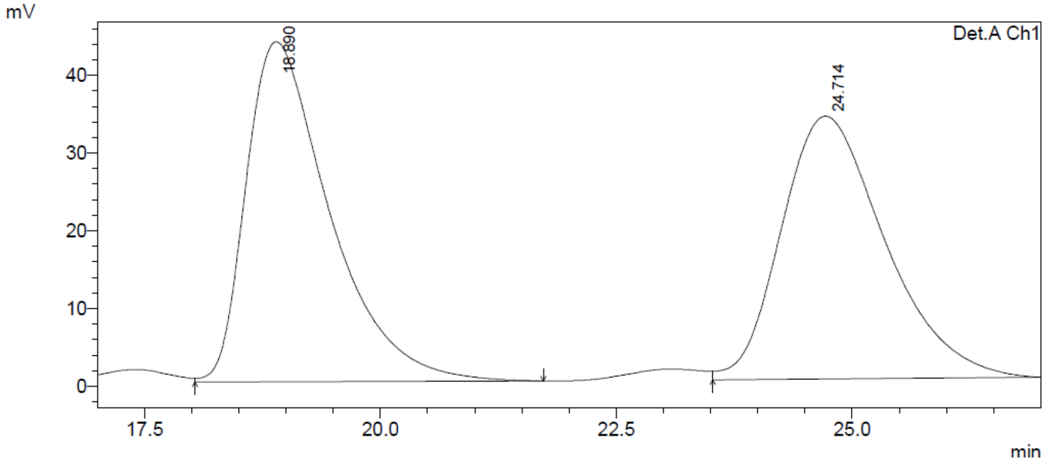

UV Detector Ch1 254nm

Peak#	Ret. Time	Area	Height	Area %	Height %
1	18.890	2697800	43741	51.046	56.395
2	24.714	2587216	33821	48.954	43.605
Total		5285016	77561	100.000	100.000

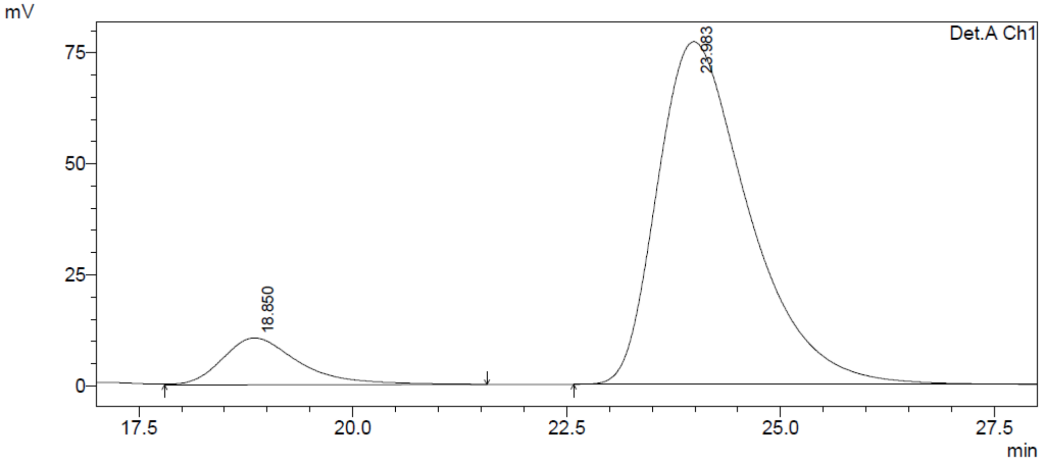

UV Detector Ch1 254nm

Peak#	Ret. Time	Area	Height	Area %	Height %
1	18.850	654916	10516	10.192	11.988
2	23.983	5771048	77207	89.808	88.012
Total		6425964	87723	100.000	100.000

Supplementary figure 51. HPLC spectra for product **3k**

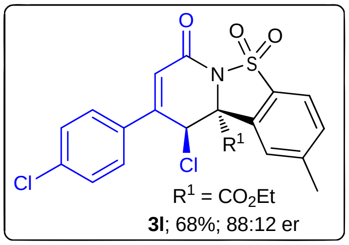

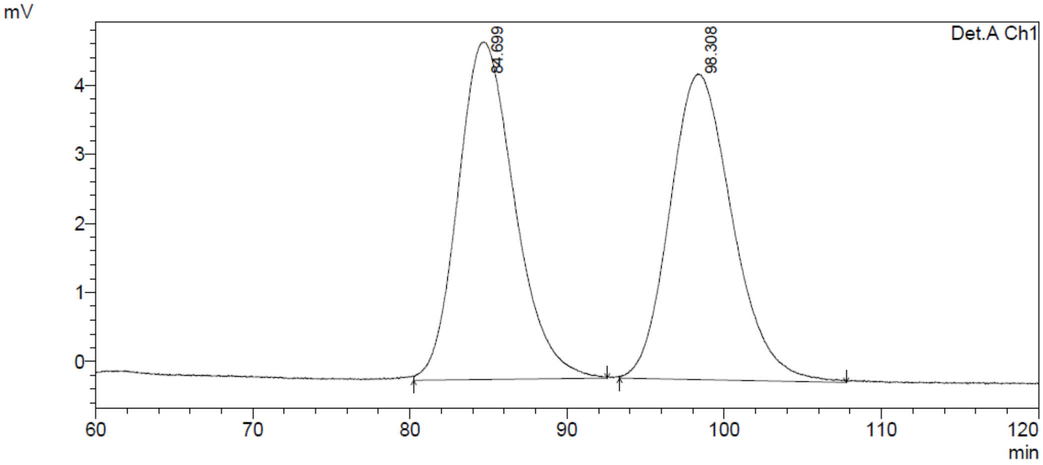

Peak#	Ret. Time	Area	Height	Area %	Height %
1	84.699	1225453	4889	49.992	52.483
2	98.308	1225842	4426	50.008	47.517
Total		2451296	9315	100.000	100.000

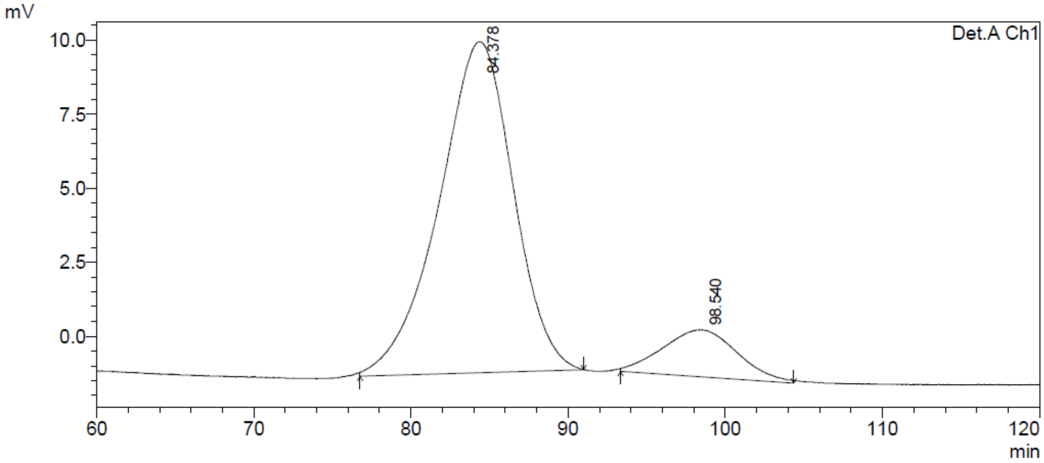

UV Detector Ch1 254nm

Peak#	Ret. Time	Area	Height	Area %	Height %
1	84.378	3676692	11178	87.503	87.524
2	98.540	525109	1593	12.497	12.476
Total		4201801	12771	100.000	100.000

Supplementary figure 52. HPLC spectra for product **3l**

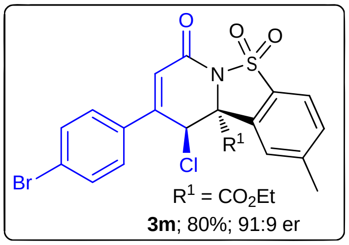

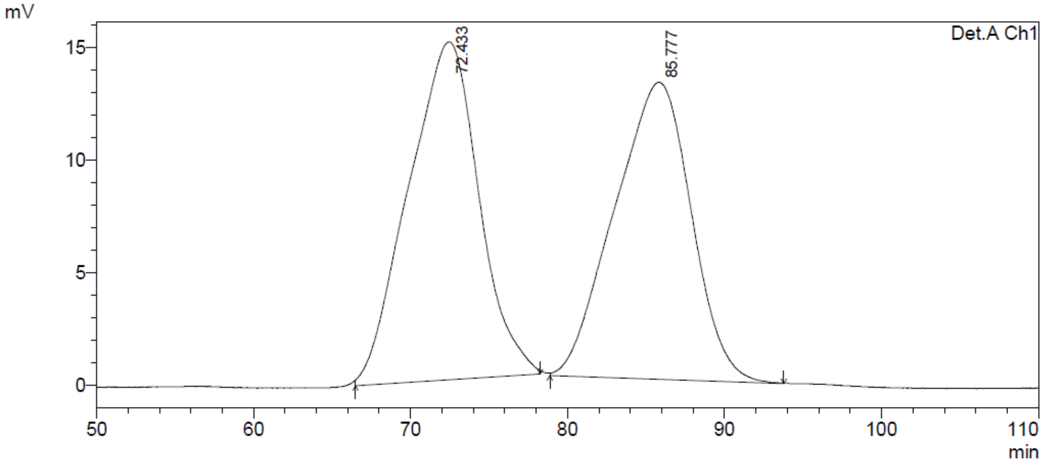

UV Detector Ch1 254nm

Peak#	Ret. Time	Area	Height	Area %	Height %
1	72.433	4695556	15027	51.060	53.207
2	85.777	4500637	13215	48.940	46.793
Total		9196192	28242	100.000	100.000

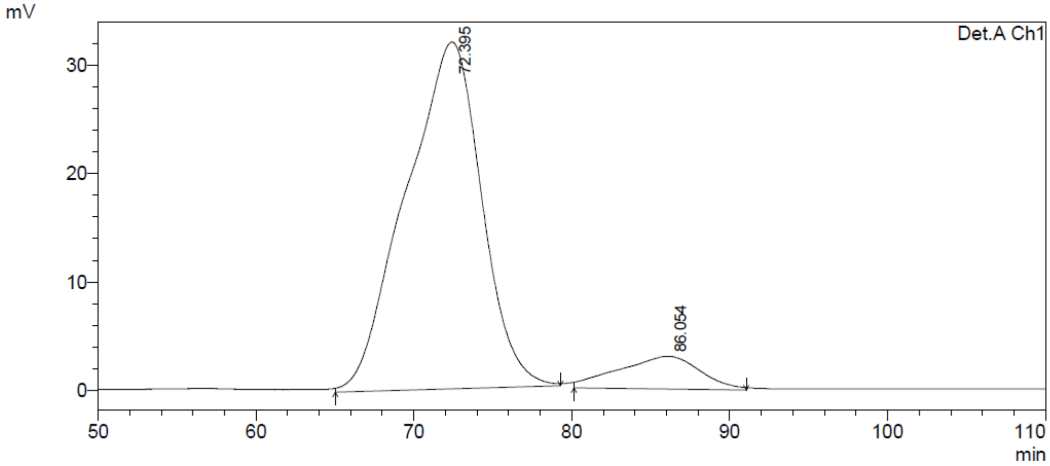

UV Detector Ch1 254nm

Peak#	Ret. Time	Area	Height	Area %	Height %
1	72.395	10646264	31942	90.744	91.374
2	86.054	1085938	3015	9.256	8.626
Total		11732202	34957	100.000	100.000

Supplementary figure 53. HPLC spectra for product **3m**

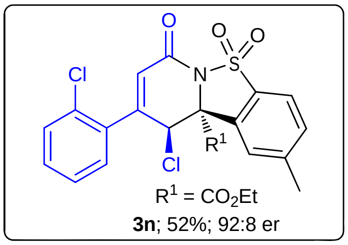

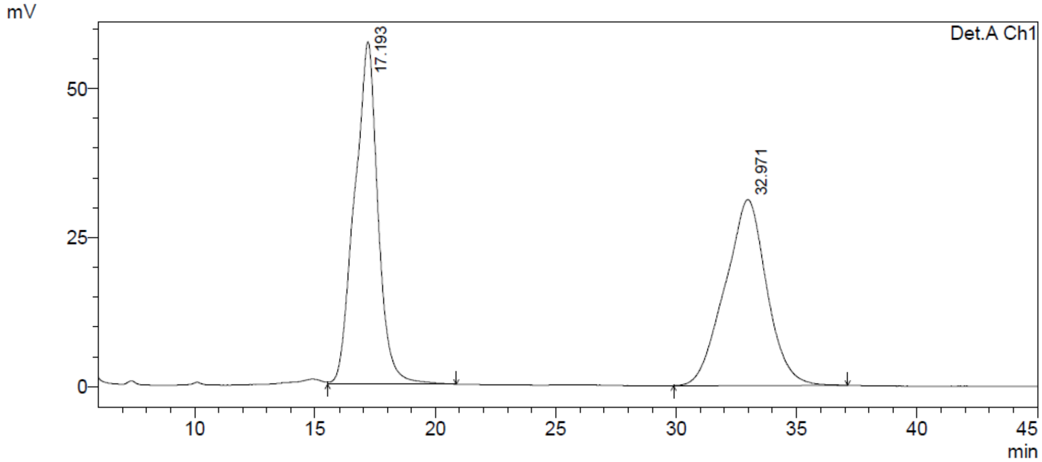

UV Detector Ch1 254nm

Peak#	Ret. Time	Area	Height	Area %	Height %
1	17.193	3899902	57444	50.879	64.814
2	32.971	3765121	31186	49.121	35.186
Total		7665023	88630	100.000	100.000

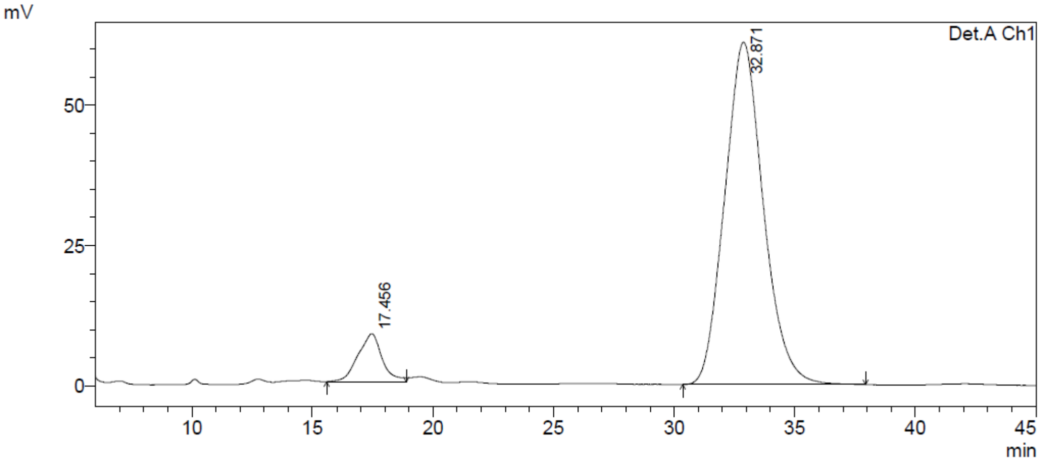

UV Detector Ch1 254nm

Peak#	Ret. Time	Area	Height	Area %	Height %
1	17.456	602373	8600	8.120	12.353
2	32.871	6815898	61020	91.880	87.647
Total		7418271	69620	100.000	100.000

Supplementary figure 54. HPLC spectra for product **3n**

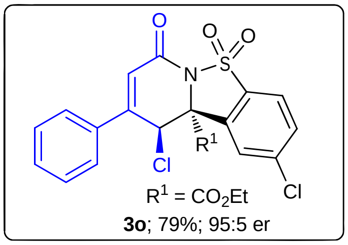

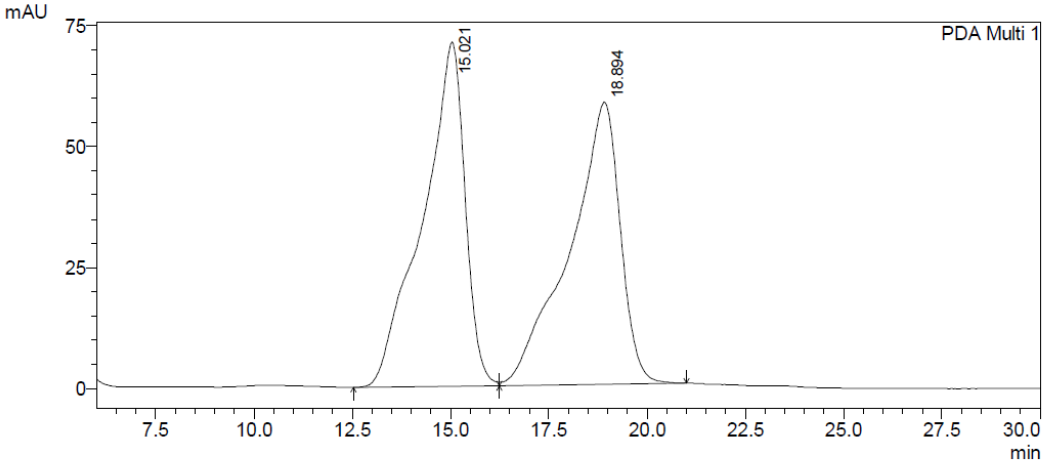

PDA Ch1 254nm 4nm

Peak#	Ret. Time	Area	Height	Area %	Height %
1	15.021	5327657	71173	50.418	54.948
2	18.894	5239416	58354	49.582	45.052
Total		10567073	129527	100.000	100.000

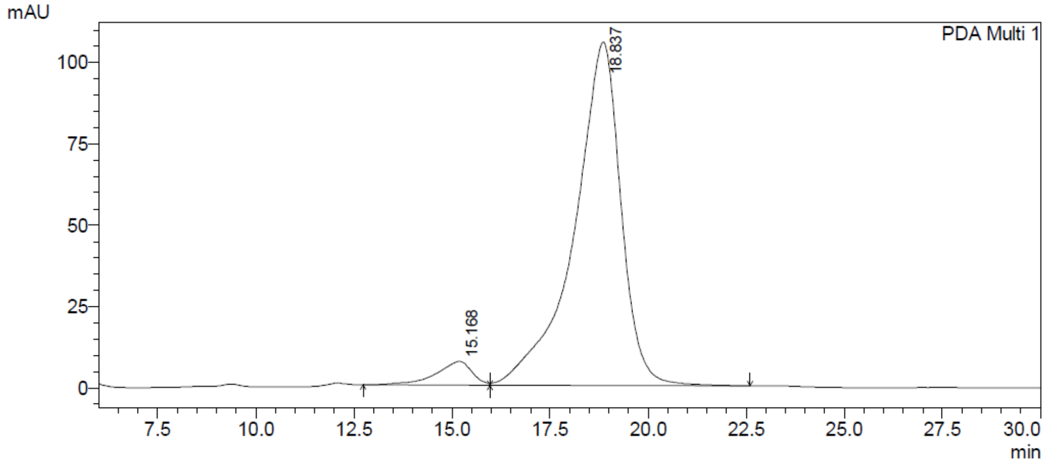

PDA Ch1 254nm 4nm

Peak#	Ret. Time	Area	Height	Area %	Height %
1	15.168	465017	7295	5.042	6.467
2	18.837	8758583	105511	94.958	93.533
Total		9223600	112806	100.000	100.000

Supplementary figure 55. HPLC spectra for product **3o**

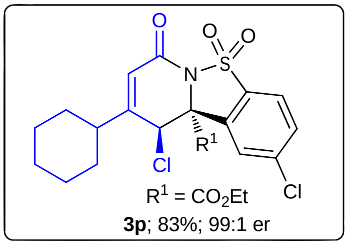

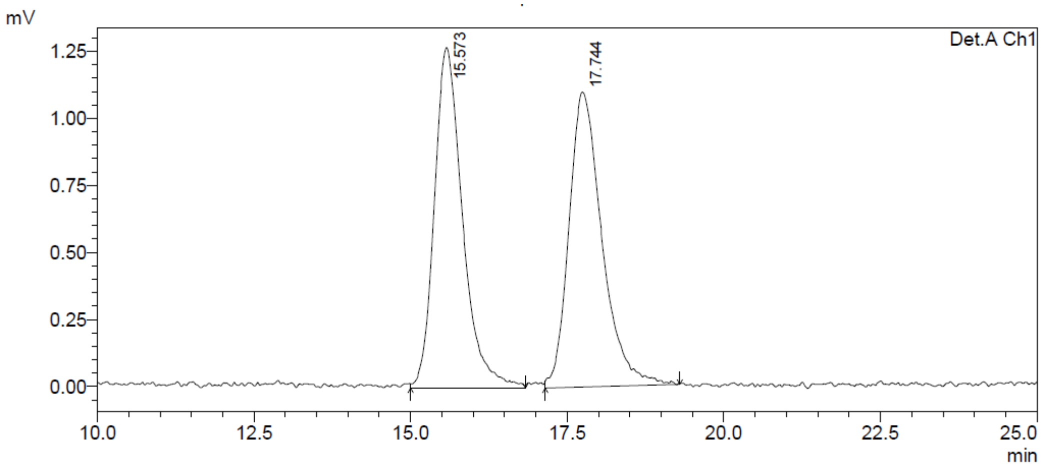

Peak#	Ret. Time	Area	Height	Area %	Height %
1	15.573	39663	1267	49.585	53.578
2	17.744	40327	1098	50.415	46.422
Total		79990	2366	100.000	100.000

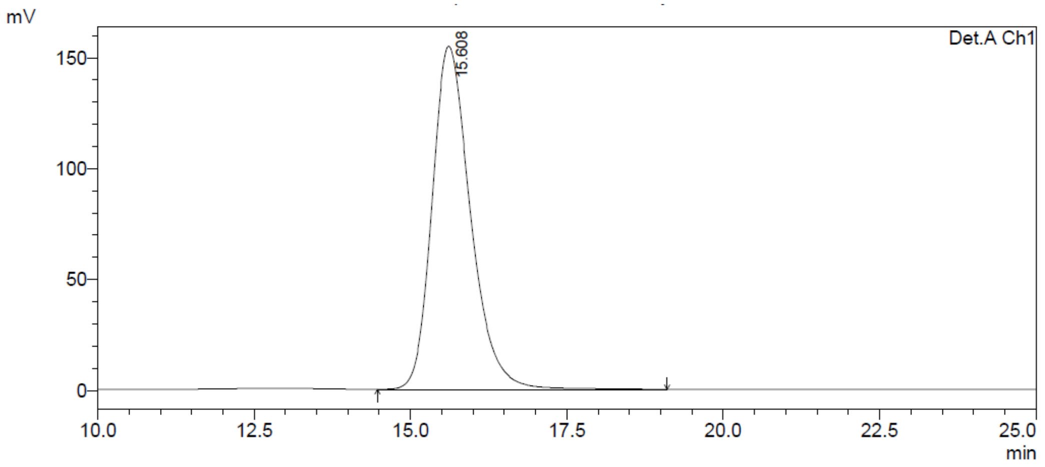

UV Detector Ch1 254nm

Peak#	Ret. Time	Area	Height	Area %	Height %
1	15.608	6629816	154642	100.000	100.000
Total		6629816	154642	100.000	100.000

Supplementary figure 56. HPLC spectra for product **3p**

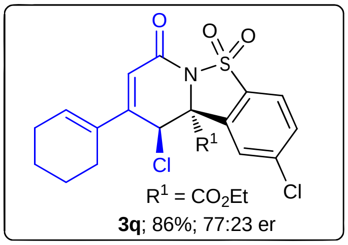

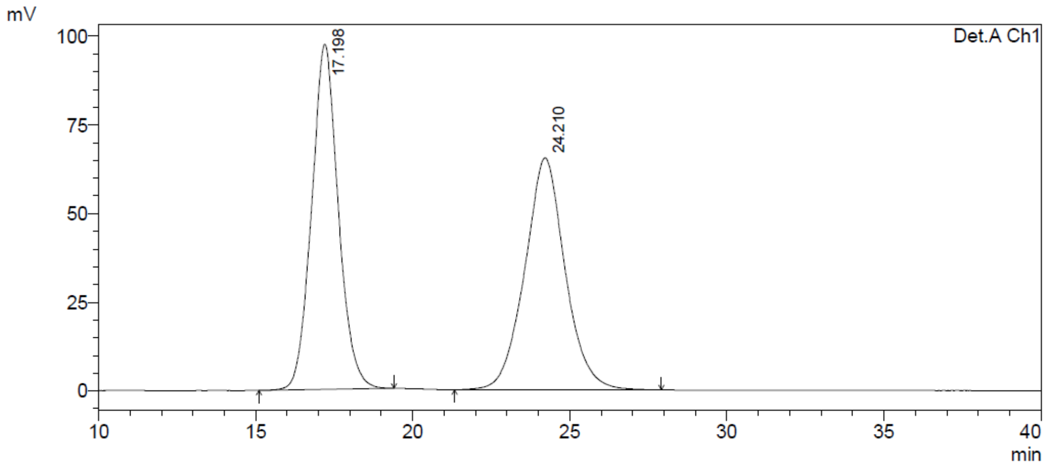

UV Detector Ch1 254nm

Peak#	Ret. Time	Area	Height	Area %	Height %
1	17.198	5747032	97273	49.530	59.788
2	24.210	5856136	65424	50.470	40.212
Total		11603168	162696	100.000	100.000

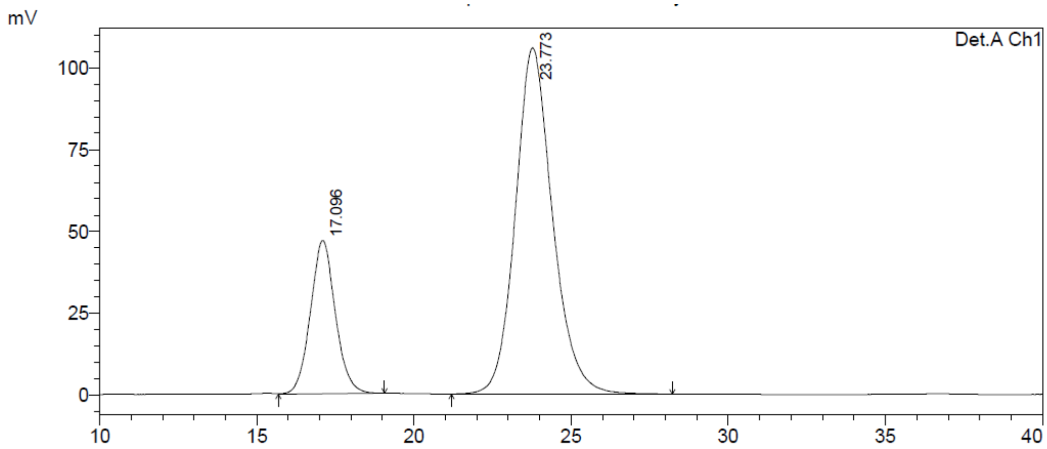

Peak#	Ret. Time	Area	Height	Area %	Height %
1	17.096	2586779	46932	22.851	30.707
2	23.773	8733651	105908	77.149	69.293
Total		11320430	152840	100.000	100.000

Supplementary figure 57. HPLC spectra for product **3q**

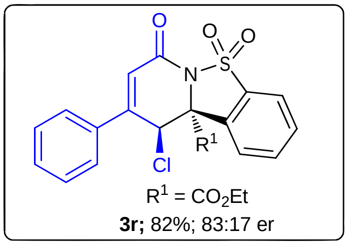

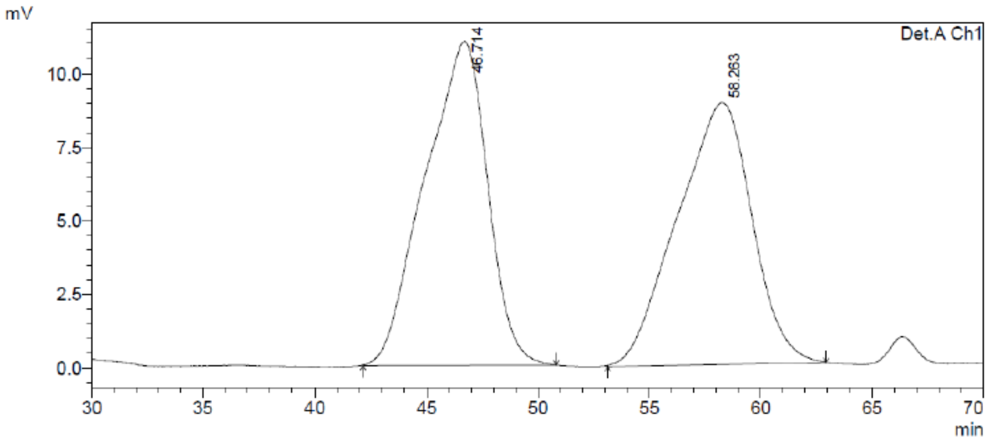

UV Detector Ch1 254nm

Peak#	Ret. Time	Area	Height	Area %	Height %
1	46.714	2156274	10968	50.920	55.176
2	58.263	2078344	8910	49.080	44.824
Total		4234618	19878	100.000	100.000

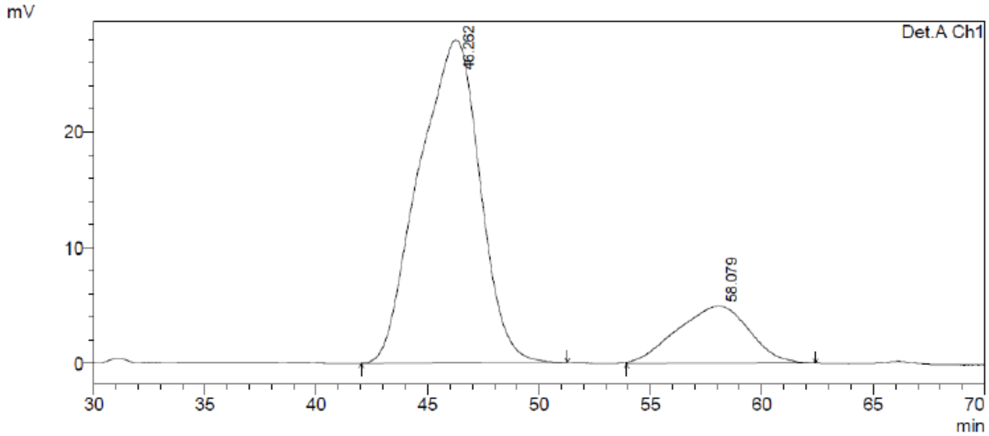

UV Detector Ch1 254nm

Peak#	Ret. Time	Area	Height	Area %	Height %
1	46.262	5331062	27933	82.667	84.941
2	58.079	1117812	4952	17.333	15.059
Total		6448875	32885	100.000	100.000

Supplementary figure 58. HPLC spectra for product **3r**

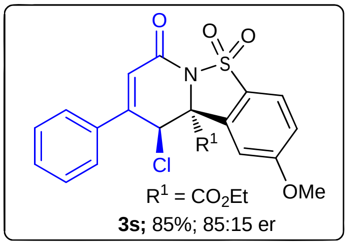

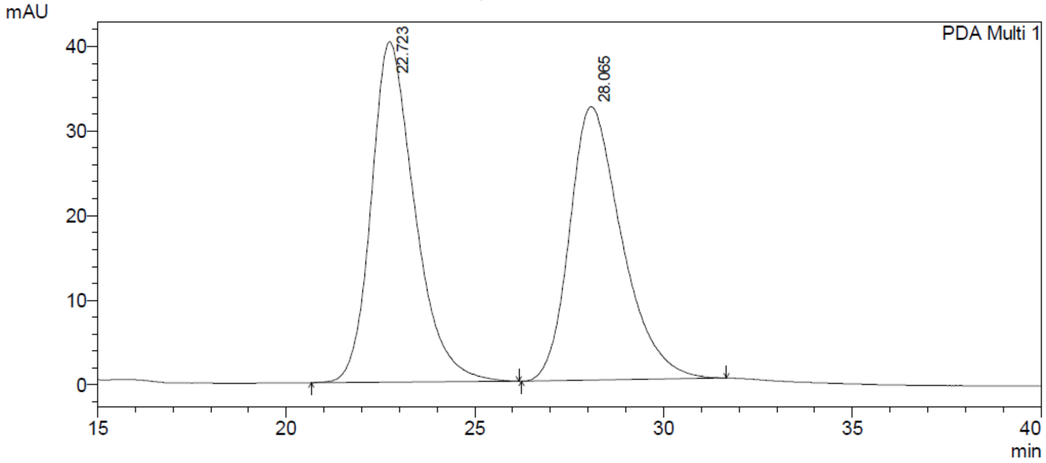

Peak#	Ret. Time	Area	Height	Area %	Height %
1	22.723	3228913	40194	50.906	55.451
2	28.065	3114030	32292	49.094	44.549
Total		6342942	72486	100.000	100.000

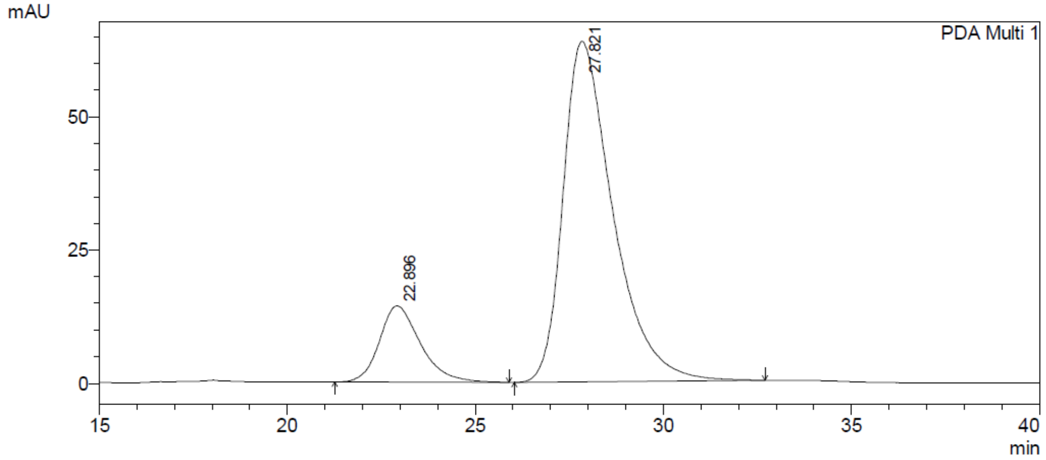

PDA Ch1 254nm 4nm

Peak#	Ret. Time	Area	Height	Area %	Height %
1	22.896	1084532	14297	15.511	18.283
2	27.821	5907422	63900	84.489	81.717
Total		6991954	78196	100.000	100.000

Supplementary figure 59. HPLC spectra for product **3s**

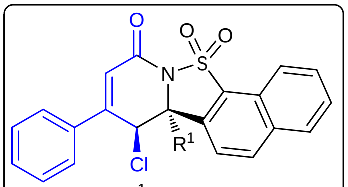

$R^1 = CO_2Et$

3t; 79%; 82:18 er

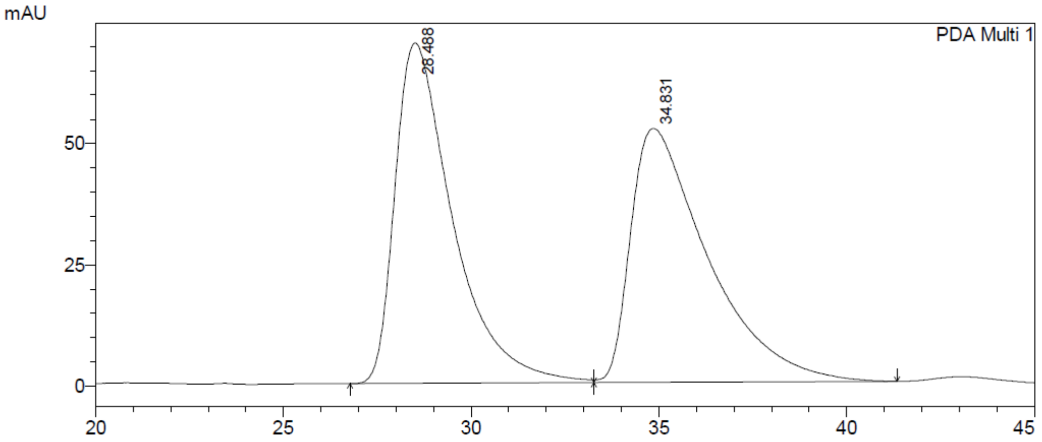

PDA Ch1 254nm 4nm

Peak#	Ret. Time	Area	Height	Area %	Height %
1	28.488	7514151	70208	50.057	57.264
2	34.831	7496903	52396	49.943	42.736
Total		15011054	122604	100.000	100.000

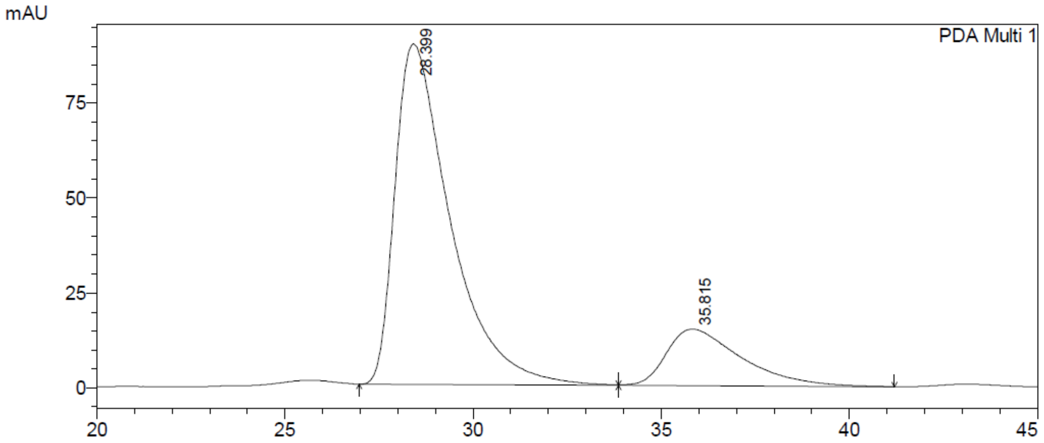

Peak#	Ret. Time	Area	Height	Area %	Height %
1	28.399	9199816	89698	81.593	85.749
2	35.815	2075383	14907	18.407	14.251
Total		11275198	104605	100.000	100.000

Supplementary figure 60. HPLC spectra for product **3t**

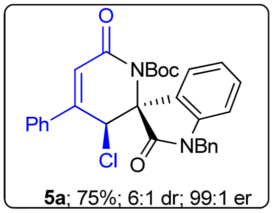

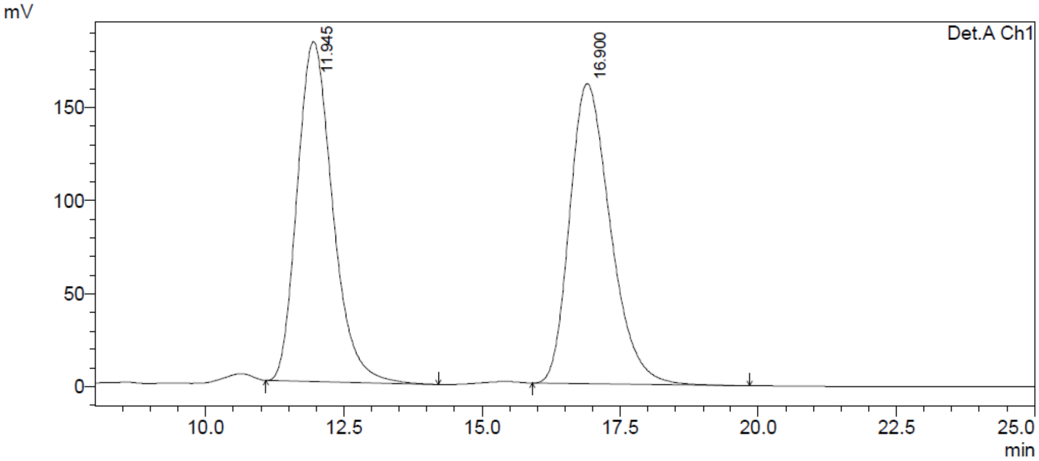

Peak#	Ret. Time	Area	Height	Area %	Height %
1	11.945	8165539	182671	49.651	53.117
2	16.900	8280185	161233	50.349	46.883
Total		16445724	343904	100.000	100.000

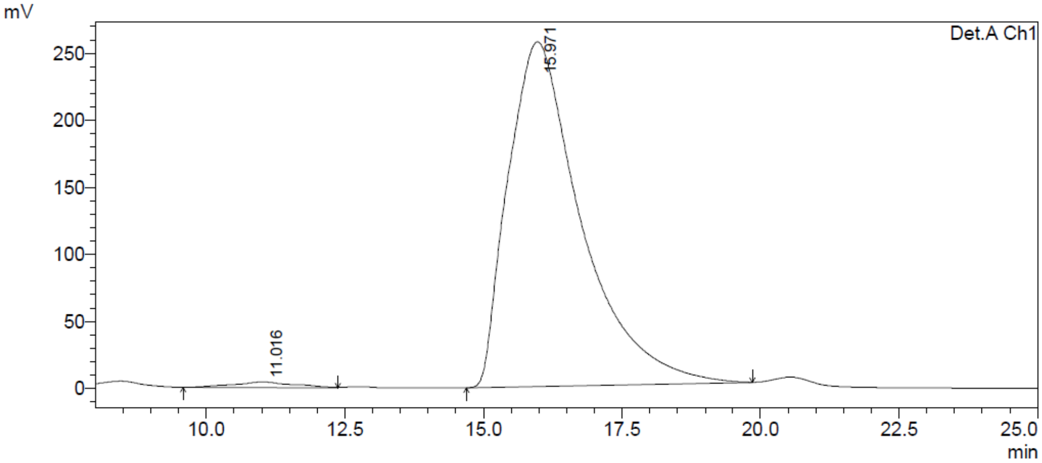

UV Detector Ch1 254nm

Peak#	Ret. Time	Area	Height	Area %	Height %
1	11.016	328039	4180	1.370	1.600
2	15.971	23622657	257016	98.630	98.400
Total		23950697	261196	100.000	100.000

Supplementary figure 61. HPLC spectra for product **5a**

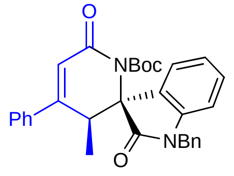

5b; 71%; 7:1 dr: >99:1 er

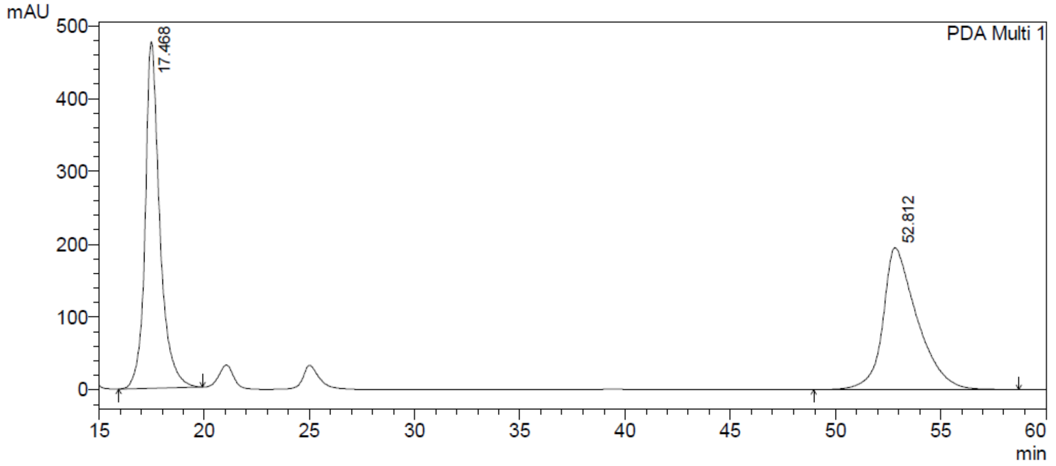

PDA Ch1 254nm 4nm

Peak#	Ret. Time	Area	Height	Area %	Height %
1	17.468	23054859	475660	51.076	70.934
2	52.812	22083562	194904	48.924	29.066
Total		45138421	670565	100.000	100.000

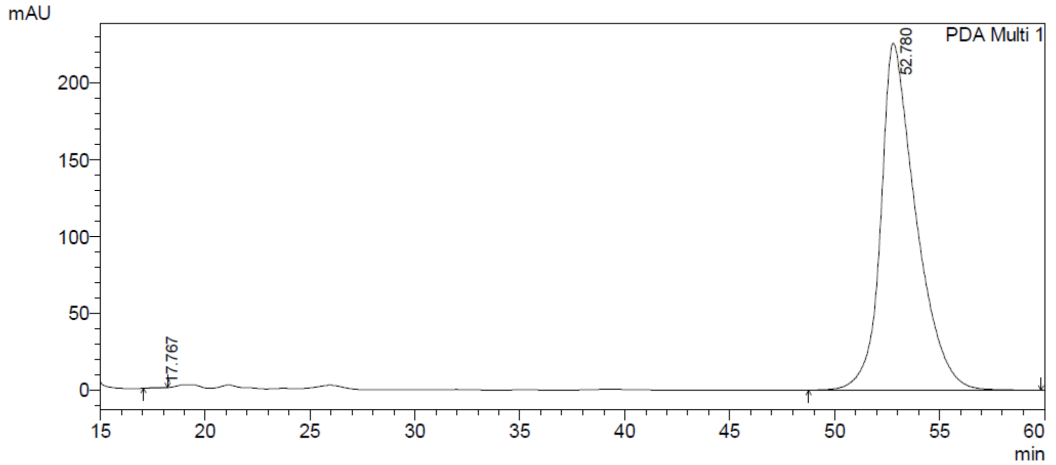

PDA Ch1 254nm 4nm

Peak#	Ret. Time	Area	Height	Area %	Height %
1	17.767	10793	246	0.041	0.109
2	52.780	26453795	225707	99.959	99.891
Total		26464588	225953	100.000	100.000

Supplementary figure 62. HPLC spectra for product **5b**

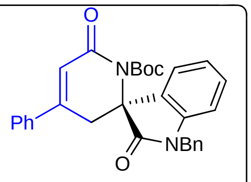

5c; 81%; 93:7 er

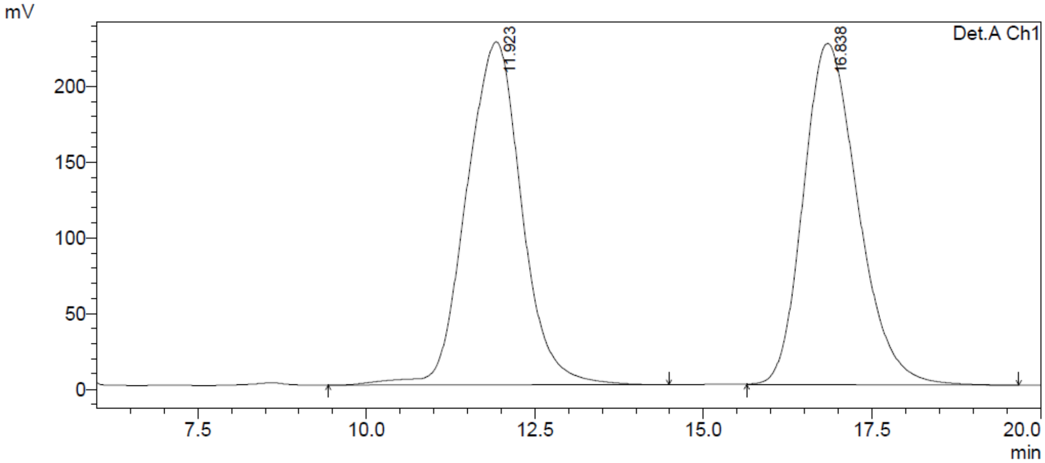

UV Detector Ch1 254nm

Peak#	Ret. Time	Area	Height	Area %	Height %
1	11.923	12948895	226898	50.328	50.123
2	16.838	12780108	225781	49.672	49.877
Total		25729002	452679	100.000	100.000

C:\Users\cbcuser05\Desktop\UV\Data\lbs\lbs235.lcd

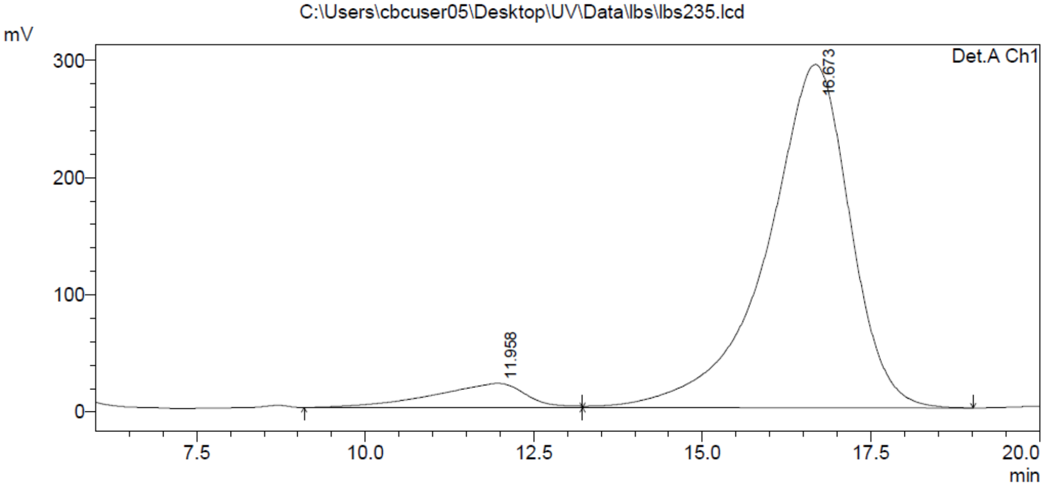

UV Detector Ch1 254nm

Peak#	Ret. Time	Area	Height	Area %	Height %
1	11.958	1942408	20583	7.153	6.566
2	16.673	25213866	292910	92.847	93.434
Total		27156274	313493	100.000	100.000

Supplementary figure 63. HPLC spectra for product **5c**

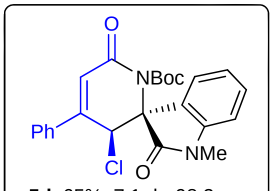

5d; 65%; 7:1 dr; 98:2 er

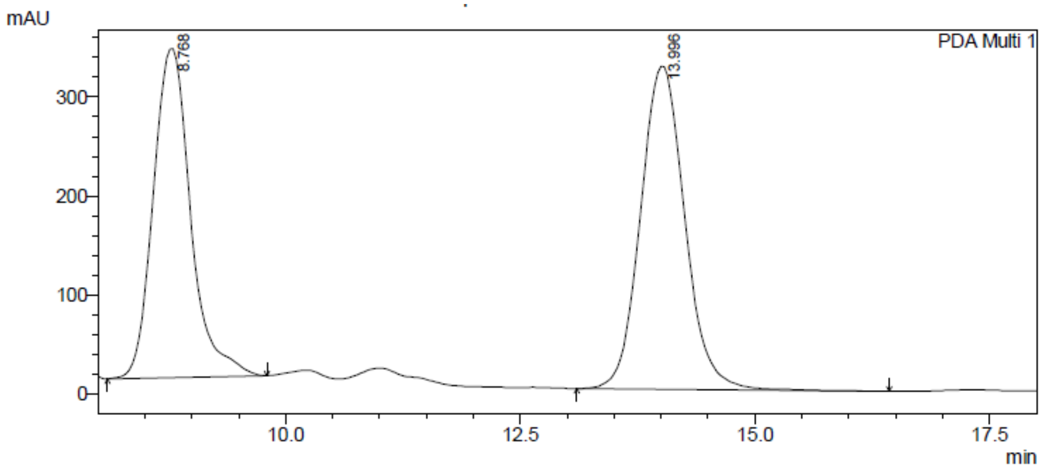

PDA Ch1 254nm 4nm

Peak#	Ret. Time	Area	Height	Area %	Height %
1	8.768	9204879	331640	46.222	50.416
2	13.996	10709432	326167	53.778	49.584
Total		19914311	657807	100.000	100.000

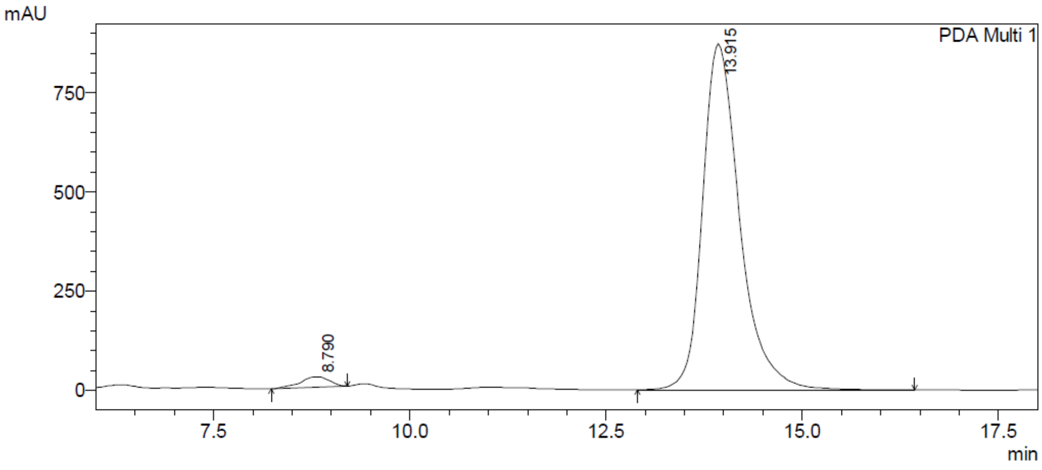

Peak#	Ret. Time	Area	Height	Area %	Height %
1	8.790	677666	26195	2.303	2.919
2	13.915	28747192	871247	97.697	97.081
Total		29424858	897442	100.000	100.000

Supplementary figure 64. HPLC spectra for product **5d**

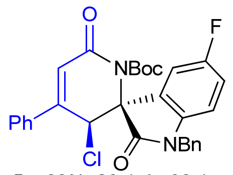

5e; 83%; 20:1 dr; 99:1 er

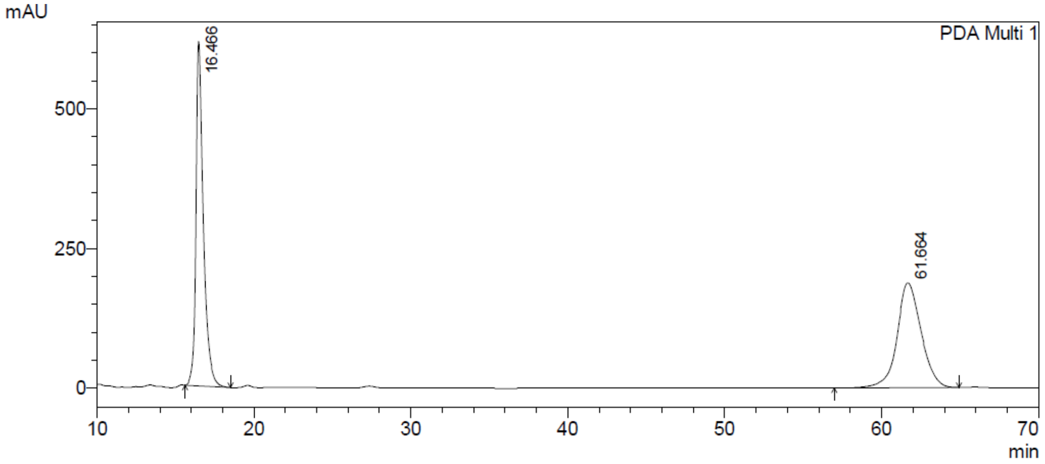

PDA Ch1 254nm 4nm

Peak#	Ret. Time	Area	Height	Area %	Height %
1	16.466	19826908	616103	49.732	76.629
2	61.664	20040644	187901	50.268	23.371
Total		39867552	804004	100.000	100.000

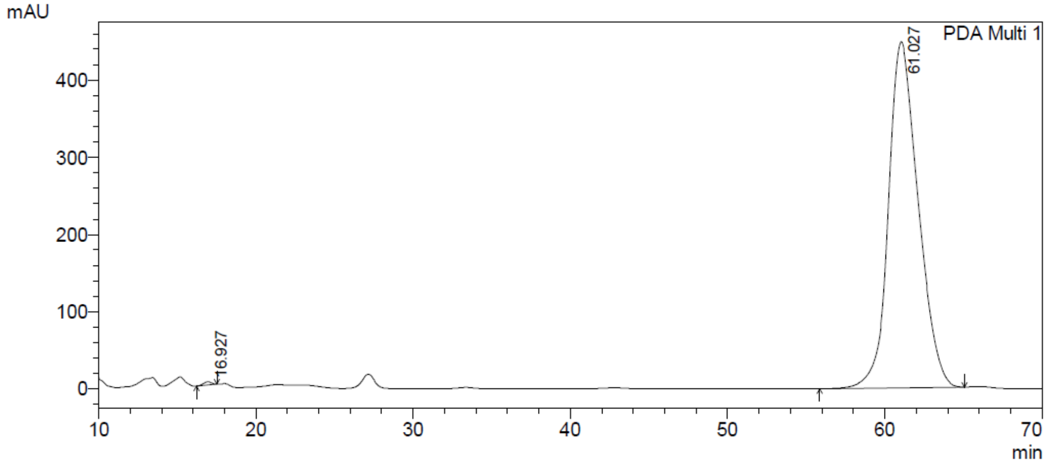

PDA Ch1 254nm 4nm

Peak#	Ret. Time	Area	Height	Area %	Height %
1	16.927	158215	4297	0.268	0.950
2	61.027	58930264	448190	99.732	99.050
Total		59088479	452487	100.000	100.000

Supplementary figure 65. HPLC spectra for product **5e**

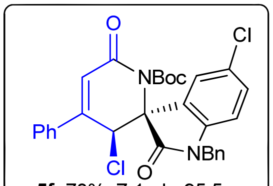

5f; 73%; 7:1; dr; 95:5 er

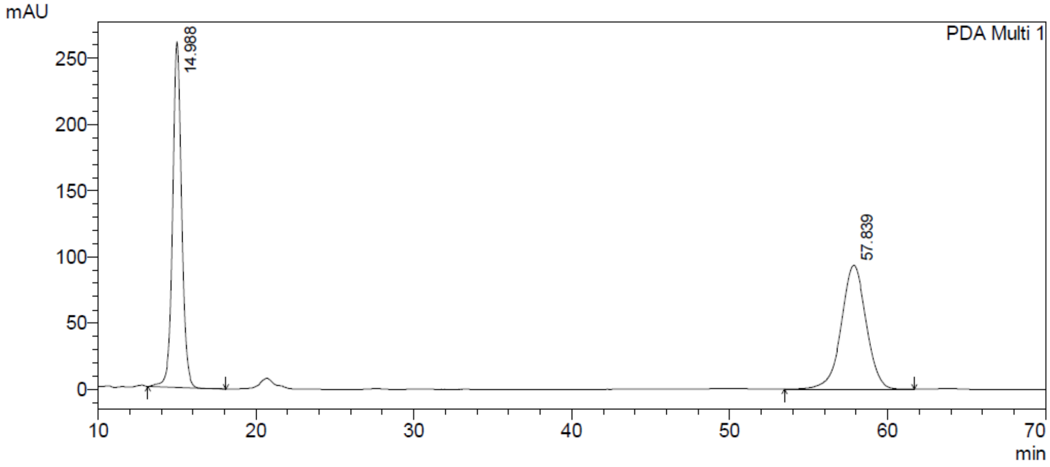

PDA Ch1 254nm 4nm

Peak#	Ret. Time	Area	Height	Area %	Height %
1	14.988	10177759	260969	49.733	73.586
2	57.839	10286908	93674	50.267	26.414
Total		20464667	354643	100.000	100.000

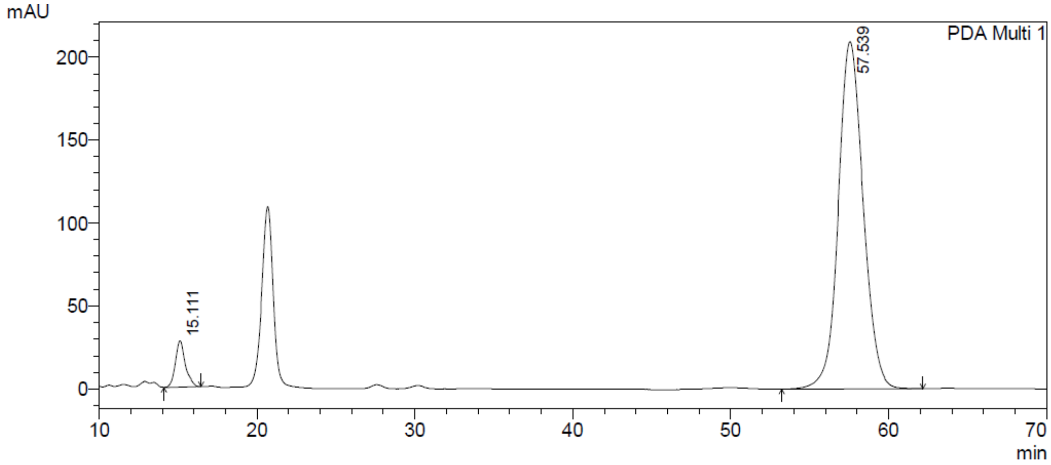

PDA Ch1 254nm 4nm

Peak#	Ret. Time	Area	Height	Area %	Height %
1	15.111	1211432	27929	5.017	11.778
2	57.539	22936295	209195	94.983	88.222
Total		24147727	237124	100.000	100.000

Supplementary figure 66. HPLC spectra for product **5f**

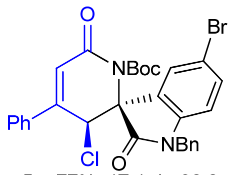

5g; 77%; 17:1 dr; 98:2 er

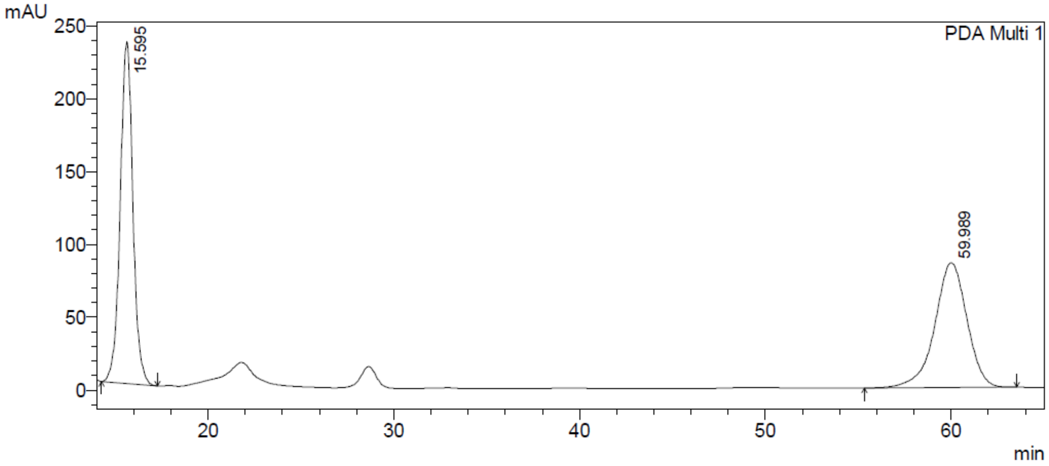

PDA Ch1 254nm 4nm

Peak#	Ret. Time	Area	Height	Area %	Height %
1	15.595	11181727	234764	51.902	73.223
2	59.989	10362378	85853	48.098	26.777
Total		21544105	320617	100.000	100.000

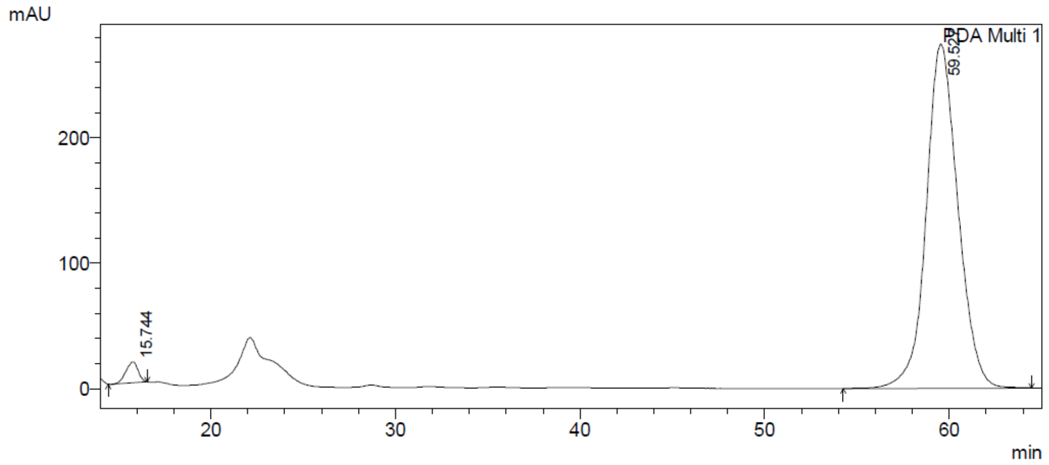

Peak#	Ret. Time	Area	Height	Area %	Height %
1	15.744	757633	16599	2.257	5.709
2	59.527	32805891	274165	97.743	94.291
Total		33563524	290764	100.000	100.000

Supplementary figure 67. HPLC spectra for product **5g**

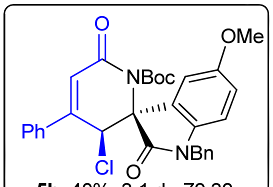

5h, 40%; 3:1 dr, 70:30 er

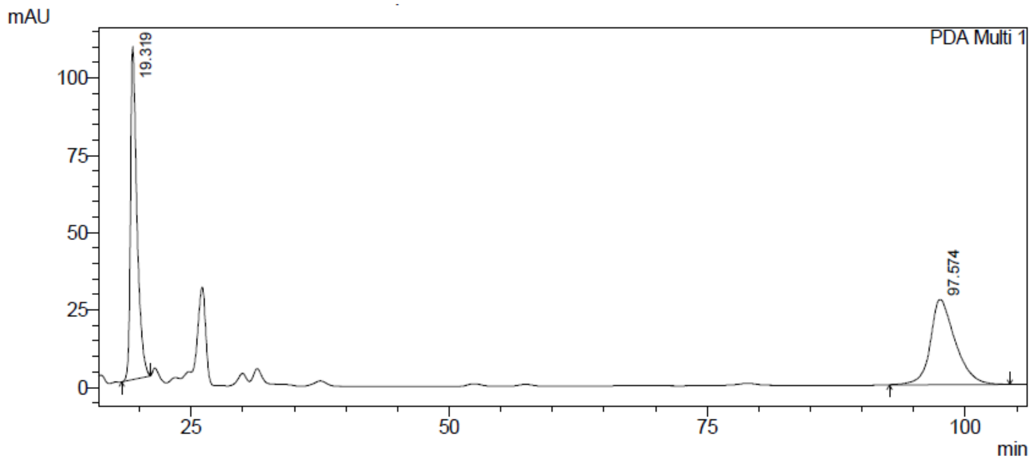

Peak#	Ret. Time	Area	Height	Area %	Height %
1	19.319	4675443	107621	50.264	79.629
2	97.574	4626282	27532	49.736	20.371
Total		9301725	135153	100.000	100.000

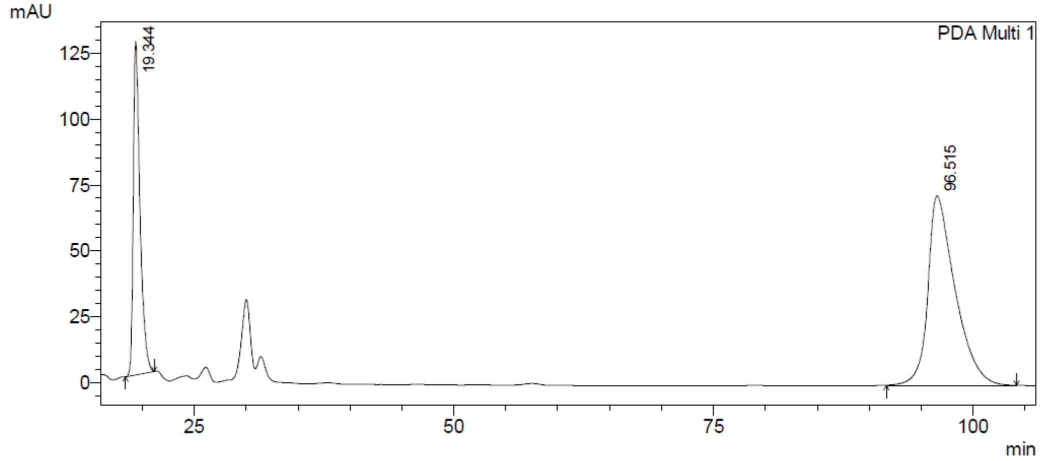

PDA Ch1 254nm 4nm

Peak#	Ret. Time	Area	Height	Area %	Height %
1	19.344	5685427	126500	30.221	63.760
2	96.515	13127635	71901	69.779	36.240
Total		18813062	198400	100.000	100.000

Supplementary figure 68. HPLC spectra for product **5h**

Supplementary Methods

General Information

All reactions were carried out under standard conditions using N_2 as shielding gas with magnetic stirring. Analytical thin layer chromatography (TLC) was performed with TLC plates. All reactions and column chromatography were monitored by thin layer chromatography with UV light under a 254 nm and colorized with ethanol solution of phosphomolybdic acid, followed by heating using a heat gun. All products could be purified by column chromatography using ethyl acetate and hexane as eluent. Organic solutions were concentrated by rotary evaporation. All solvents were freshly distilled before use. ^{1}H and ^{13}C NMR chemical shifts are reported in $CDCl_3$ solution of compound by Bruker AV-300 MHz or Bruker AV-400 MHz instruments and marked in ppm relative to tetramethylsilane (TMS) (0) and $CDCl_3$ (77.0 ppm) as standard. The following abbreviations are used to describe peak patterns where appropriate: s = singlet, d = doublet, t = triplet, q = quartet, m = multiplet. Coupling constants (*J*) are reported in Hertz (Hz). High resolution mass spectral analysis (HRMS) was performed on Waters Q-TOF Premier mass spectrometer. The determination of *ee* was performed *via* chiral phase HPLC analysis using Shimadzu LC-20AD HPLC workstation. Optical rotations were measured using a 1 mL cell with a 1 cm path length on a Jasco P1030 digital polarimeter and are reported as follows: $[\alpha]^D_{20}$. The dr values of the products were determined by the corresponding ^{1}H NMR spectra. The absolute and relative configuration of products could be assigned by the X-ray structures of **3b**, **3m**, **5e**, and **5f**.

General procedure for preparation of substrates

All imine substrates **2**[1] and **4**[2] were prepared in accordance with the literatures. Cyclobutenones **1b** and **1c** were synthesized by the known literature process.[3] The other cyclobutenones **1** were prepared according to the following methods:

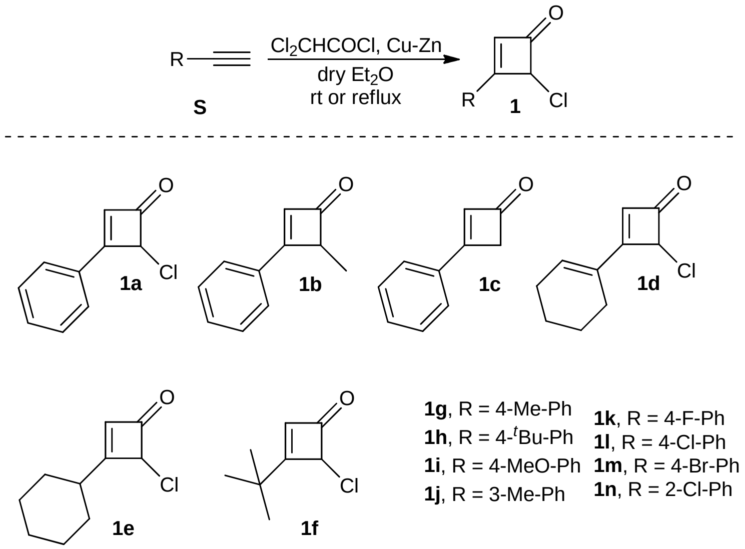

Under argon atmosphere, to a solution of alkyne **S1** (10.0 mmol) and zinc-copper couple

(4.0 equiv, 40.0 mmol, 2.6 g) in dry Et_2O (20.0 mL) was dissolved in 100 mL round-bottom flask equipped with condenser pipe at 0 °C. Then the dichloroacetyl chloride (2.0 equive, 20 mmol, 1.90 mL) was added into the reaction system by syringe at current temperature. After stirring for 15 min, the reaction was allowed to warm to room temperature (or reflux for **1d-1f**, **1k-1n**) and stirred for further 5 h. The resulting mixture was filtered through celite. The filtrate was washed with each of ice-cold 0.5 M hydrochloric acid, ice-cold saturated sodium bicarbonate solution, and brine. The organic phase was dried over $MgSO_4$ and concentrated under vacuum and purified by column chromatography to give ketones **l**.

General procedure for syntheses of products 3 and 5

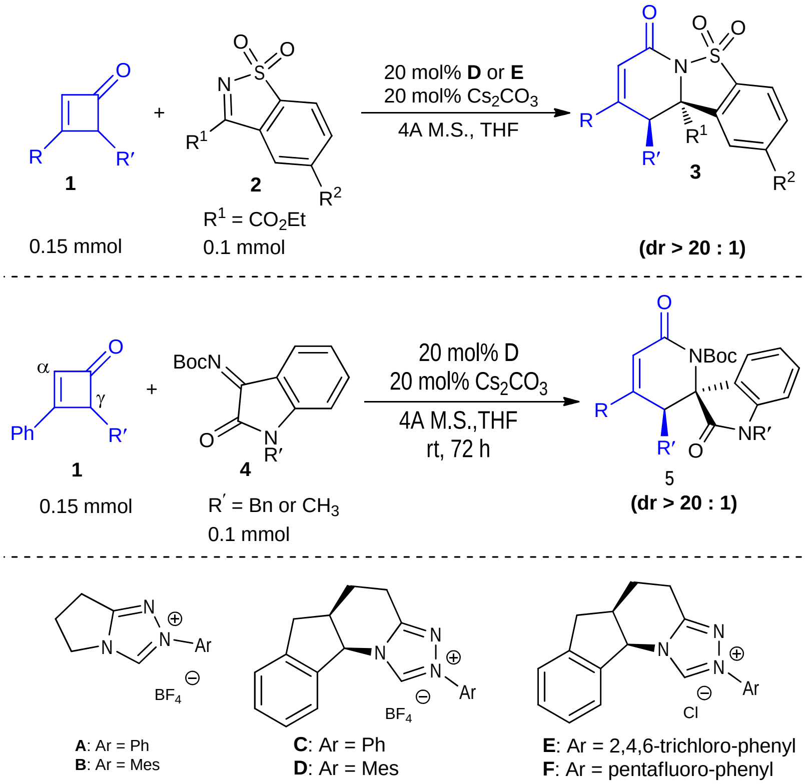

To a dry Schlenk tube equipped with a magnetic stir bar, were added cyclobuteneone **1** (0.15 mmol), imines **2** or **4** (0.1 mmol), triazolium salt **D** or **E** (0.02 mmol), Cs_2CO_3 (0.02 mmol, 6.5 mg), 4A molecular sieve (100 mg). The tube was closed with a septum, evacuated, and refilled with nitrogen. Then, the freshly distilled THF (1.0 mL) was added. The reaction mixture was stirred at the specified temperature as showed in Chart 1 and Chart 2 in the text. After completed consumption of imines by TLC monitoring, the mixture was concentrated under reduced pressure. The resulting crude residue was purified *via* column chromatography on silica gel to afford the desired product **3** or **5**.

Note: Racemic samples for the chiral phase HPLC analysis were prepared using **B** as the NHC pre-catalyst.

Characterization of substrates

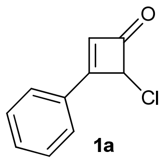

1a

4-chloro-3-phenylcyclobut-2-enone (1a): light yellow solid, 77% yield**.** ^{1}H NMR (400 MHz, $CDCl_3$): δ 7.78-7.75 (m, 2H), 7.61-7.53 (m, 3H), 6.59 (d, *J* = 0.4Hz, 1H), 5.64 (d, *J* = 0.8 Hz, 1H), ^{13}C NMR (100 MHz, $CDCl_3$): δ182.0, 172.5, 133.1, 132.5, 129.5, 129.3, 128.4, 66.9; IR ν (cm^{-1}) 1759, 1597, 1458, 1373, 1034, 802; HRMS (ESI) calcd. For $C_{10}H_7ClO$ $[M+H]^+$: 179.0264, Found: 179.0265.

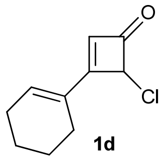

1d

4-chloro-3-(cyclohex-1-en-1-yl)cyclobut-2-enone (1d): light yellow oil, 78% yield. ^{1}H NMR (400 MHz, $CDCl_3$): δ 6.72 (s, 1H), 6.07 (s, 1H), 5.36 (s, 1H), 2.42-2.28 (m, 4H), 1.79-1.62 (m, 4H); ^{13}C NMR (100 MHz, $CDCl_3$): δ 182.7, 173.5, 143.6, 130.5, 129.9, 66.4, 26.5, 24.6, 21.5, 21.0; HRMS (ESI) calcd. For $C_{10}H_{11}ClO$ $[M+H]^+$: 183.0577, Found: 183.0567.

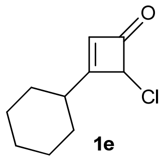

1e

4-chloro-3-cyclohexylcyclobut-2-enone (1e): light yellow oil, 67% yield. ^{1}H NMR (400 MHz, $CDCl_3$): δ 6.13 (t, *J* = 1.2 Hz, 1H), 5.23 (s, 1H), 2.67-2.64 (m, 1H), 2.05-2.00 (m, 2H), 1.84-1.80 (m, 2H), 1.79-1.71 (m, 1H), 1.48-1.26 (m, 5H); ^{13}C NMR (100 MHz, $CDCl_3$): δ 185.9, 182.3, 135.7, 67.7, 37.7, 29.3, 29.2, 25.6, 25.1; HRMS (ESI) calcd. For $C_{10}H_{13}ClO$ $[M+H]^+$: 185.0733, Found: 185.0742.

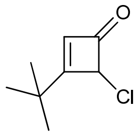

1f

3-(tert-butyl)-4-chlorocyclobut-2-enone (1f): light yellow oil, 61% yield. ^{1}H NMR (400 MHz, $CDCl_3$): δ 6.14 (s, 1H), 5.27 (s, 1H), 1.33 (s, 9H); ^{13}C NMR (100 MHz, $CDCl_3$): δ

189.8, 182.1, 135.6, 67.1, 35.1, 27.8; HRMS (ESI) calcd. For $C_8H_{11}ClO$ $[M+H]^+$: 159.0577, Found: 159.0579.

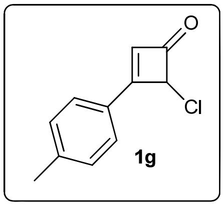

4-Chloro-3-(p-tolyl)cyclobut-2-en-1-one (1g): light yellow solid, 78% yield. ^1H NMR (400 MHz, $CDCl_3$): δ 7.66 (d, *J* = 8.0 Hz,, 2H), 7.35 (d, *J* = 8.0 Hz,, 2H), 6.52 (s, 1H), 5.62 (s, 1H), 2.46 (s, 3H). ^{13}C NMR (100 MHz, $CDCl_3$): δ 182.1, 172.4, 144.4, 131.4, 130.0, 129.6, 125.8, 66.9, 21.9; IR ν (cm^{-1}) 1751, 1605, 1458, 1373, 1034, 826, 725; HRMS (ESI) calcd. For $C_{11}H_9ClO$ $[M+H]^+$: 193.0420, Found: 193.0425.

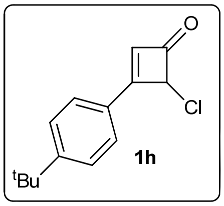

3-(4-(tert-butyl)phenyl)-4-chlorocyclobut-2-enone (1h): light yellow solid, 71% yield. ^1H NMR (400 MHz, $CDCl_3$): δ 7.70 (d, *J* = 8.4 Hz, 2H), 7.57 (d, *J* = 8.4 Hz, 2H), 6.53 (d, *J* = 0.8 Hz, 1H), 5.62 (d, *J* = 0.8 Hz, 1H), 1.36 (s, 9H); ^{13}C NMR (100 MHz, $CDCl_3$): δ 182.1, 172.4, 157.3, 131.5, 129.5, 126.3, 125.7, 66.9, 35.3, 31.0; HRMS (ESI) calcd. For $C_{14}H_{15}ClO$ $[M+H]^+$: 235.0890, Found: 235.0892.

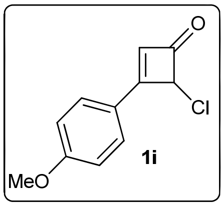

4-Chloro-3-(4-methoxyphenyl)cyclobut-2-en-1-one (1i): light yellow solid, 75% yield. ^1H NMR (400 MHz, $CDCl_3$): δ 7.74-7.69 (m, 2H), 7.06-7.01 (m, 2H), 6.43 (d, *J* = 1.0 Hz, 1H), 5.59 (d, *J* = 1.0 Hz, 1H), 3.90(s, 3H). ^{13}C NMR (100 MHz, $CDCl_3$): δ 181.9, 171.9, 163.6, 131.8, 129.7, 121.3, 114.8, 66.9, 55.6; IR ν (cm^{-1}) 1739, 1679, 1450, 1348, 1041, 873; HRMS (ESI) calcd. For $C_{11}H_9ClO_2$ $[M+H]^+$: 209.0293, Found: 209.0298.

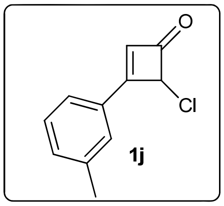

4-chloro-3-(m-tolyl)cyclobut-2-enone (1j): light yellow solid, 55% yield. ^{1}H NMR (400 MHz, $CDCl_3$): δ 7.57-7.56 (m, 2H), 7.45-7.38 (m, 2H), 6.56 (s, 1H), 5.62 (s, 1H), 2.44 (s, 3H); ^{13}C NMR (100 MHz, $CDCl_3$): δ 182.0, 172.7, 139.1, 134.0, 132.3, 129.9, 129.1, 128.3, 126.7, 66.9, 21.2; HRMS (ESI) calcd. For $C_{11}H_9ClO$ $[M+H]^+$: 193.0420, Found: 193.0429.

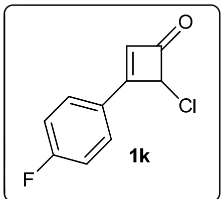

1k

4-chloro-3-(4-fluorophenyl)cyclobut-2-enone (1k): light yellow solid, 40% yield. ^{1}H NMR (400 MHz, $CDCl_3$): δ 7.81-7.77 (m, 2H); 7.27-7.22 (m, 2H); 6.55 (d, J = 0.4 Hz, 1H); 5.63 (d, J = 0.4 Hz, 1H); ^{13}C NMR (100 MHz, $CDCl_3$): δ 181.5, 171.1, 166.7, 164.1, 132.1, 132.0, 125.0, 124.9, 116.9, 116.7, 66.9; IR ν (cm^{-1}) 1763, 1600, 1497, 1240, 1037, 849; HRMS (ESI) calcd. For $C_{10}H_6ClFO$ $[M+H]^+$: 197.0158, Found: 197.0163.

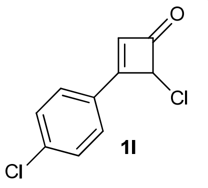

1l

4-chloro-3-(4-chlorophenyl)cyclobut-2-enone (1l): light yellow solid, 43% yield. ^{1}H NMR (400 MHz, $CDCl_3$): δ 7.55-7.52 (m, 2H)，7.72-7.67 (m, 2H), 5.623 (d, J = 1.2 Hz, 1H), 6.59 (d, J = 1.2 Hz, 1H); ^{13}C NMR (100 MHz, $CDCl_3$): 181.5, 171.0, 139.6, 132.9, 130.7, 129.8, 126.9, 66.9； HRMS (ESI) calcd. For $C_{10}H_6Cl_2O$ $[M+H]^+$: 212.9874, Found: 212.9870.

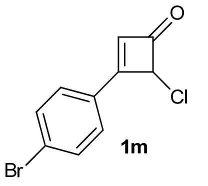

1m

3-(4-bromophenyl)-4-chlorocyclobut-2-enone (1m): light yellow solid, 48% yield. ^{1}H NMR (400 MHz, $CDCl_3$): δ 7.69 (d, J = 8.4 Hz, 2H), 7.62 (d, J = 8.4 Hz, 2H), 6.61 (s, 1H), 5.63 (s, 1H), ^{13}C NMR (100 MHz, $CDCl_3$): δ 181.5, 171.1, 133.1, 132.8, 130.8, 128.3, 127.3, 66.9; HRMS (ESI) calcd. For $C_{10}H_6BrClO$ $[M+H]^+$: 256.9369, Found: 256.9368.

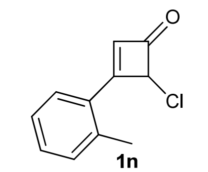

1n

4-chloro-3-(2-chlorophenyl)cyclobut-2-enone (1n): light yellow solid, 45% yield. ^{1}H NMR (400 MHz, $CDCl_3$): δ 7.78 (d, J = 8.4 Hz, 1H), 7.59-7.27 (m, 3H), 6.93 (d, J = 1.2 Hz, 1H), 5.71 (d, J = 1.2 Hz, 1H); ^{13}C NMR (100 MHz, $CDCl_3$): δ 182.6, 168.7, 137.7, 136.9, 132.6, 131.4, 127.4, 128.3, 126.9, 68.5; HRMS (ESI) calcd. For $C_{10}H_6Cl_2O$ $[M+H]^+$: 212.9798, Found: 212.9795.

Characterization of products

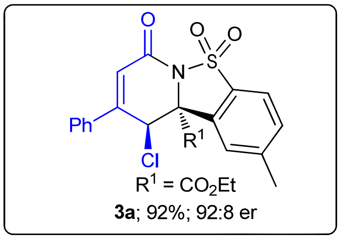

Ethyl 10-chloro-2-methyl-7-oxo-9-phenyl-7,10-dihydro-10aH-benzo[4,5]isothiazolo [2,3-a]pyridine-10a-carboxylate 5,5-dioxide (3a): Colorless oil. ^{1}H NMR (400 MHz, $CDCl_3$): δ 7.82 (d, J = 8.0 Hz, 1H), 7.65-7.60 (m, 2H), 7.57-7.46 (m, 5H), 6.44 (s, 1H), 5.90 (s, 1H), 4.29-4.13 (m, 2H), 2.55 (s, 3H), 1.17 (t, J = 7.1 Hz, 3H). ^{13}C NMR (100 MHz, $CDCl_3$): δ 167.6, 158.9, 152.4, 145.9, 133.8, 132.8, 132.6, 131.4, 129.9, 126.8, 124.0, 122.0, 121.0, 72.0, 64.6, 55.1, 22.0, 12.8; IR ν (cm^{-1}) 1763, 1600, 1497, 1240, 1037, 849; HRMS (ESI) calcd. For $C_{21}H_{18}ClNO_5S$ $[M+H]^+$: 432.0672, Found: 432.0672. $[\alpha]^{20}_{D}$ = 251.7 (c = 5.0 mg/mL, $CHCl_3$). The er value was determined by HPLC (Chiralcel ODH, hexane/isopropanol = 80:20, flow rate = 0.75 mL/min), retention time: t_1 = 20.0 min, t_2 = 26.0 min.

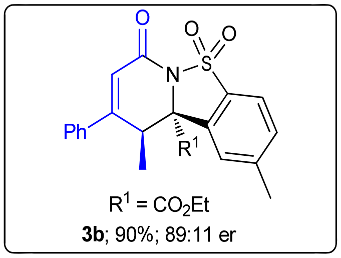

Ethyl 2,10-dimethyl-7-oxo-9-phenyl-7,10-dihydro-10aH-benzo[4,5]isothiazolo[2,3-a] pyridine-10a-carboxylate 5,5-dioxide (3b): Colorless solid, m.p. 226-228. ^{1}H NMR (400 MHz, $CDCl_3$): δ 7.79 (d, J = 8.0 Hz, 1H), 7.61-7.56 (m, 2H), 7.54-7.45 (m, 5H), 6.27 (s, 1H), 4.25-4.08 (m, 3H), 2.54 (s, 3H), 1.15 (t, J = 7.1 Hz, 3H), 0.88 (d, J = 7.0 Hz, 1H). ^{13}C NMR (100 MHz, $CDCl_3$): δ 169.7, 160.4, 159.7, 145.8, 135.6, 132.6, 132.2, 131.0, 130.8, 129.2, 127.0, 124.0, 122.0, 118.4, 72.0, 63.6, 39.5, 22.0, 13.9, 13.8; IR ν (cm^{-1}) 1739, 1679, 1450, 1348, 1191, 873; HRMS (ESI) calcd. For $C_{22}H_{21}NO_5S$ $[M+H]^+$: 412.1219, Found: 412.1214. $[\alpha]^{20}_{D}$ = 257.4 (c = 5.0 mg/mL, $CHCl_3$). The er value was determined by HPLC (Chiralcel ODH, hexane/isopropanol = 80:20, flow rate = 0.75 mL/min), retention time: t_1 = 17.8 min, t_2 = 20.6 min.

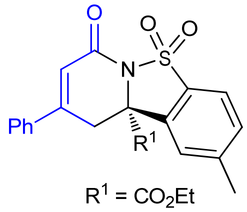

$R^1 = CO_2Et$
3c; 85%; 80:20 er

Ethyl 2-methyl-7-oxo-9-phenyl-10,10a-dihydro-7H-benzo[4,5]isothiazolo[2,3-a]pyridine -10a-carboxylate 5,5-dioxide (3c): Colorless oil. ^1H NMR (400 MHz, $CDCl_3$): δ 7.80 (d, *J* = 10.8 Hz, 1H), 7.58–7.55 (m, 3H), 7.51-7.46 (m, 4H), 6.39 (d, *J* = 3.6 Hz, 1H), 4.28-4.08 (m, 3H), 3.05-2.99 (dd, J_1 = 22.4 Hz, J_1 = 3.2 Hz, 1H), 2.54 (s, 3H), 1.18 (t, *J* = 9.6 Hz, 3H); ^{13}C NMR (100 MHz, $CDCl_3$): δ 160.5, 152.7, 145.8, 136.2, 132.6, 132.2, 130.9, 129.1, 126.5, 124.1, 122.0, 119.7, 68.0, 63.8, 36.9, 21.9, 13.8; IR ν (cm^{-1}) 1736, 1674, 1458, 1342, 1042, 771; HRMS (ESI) calcd. For $C_{21}H_{19}NO_5S$ $[M+H]^+$: 398.1062, Found: 398.1065. $[\alpha]^{20}_D$ = 142.2 (*c* = 5.0 mg/mL, $CHCl_3$). The er value was determined by HPLC (Chiralcel OD, hexane/isopropanol = 80:20, flow rate = 0.75 mL/min), retention time: t_1 = 26.3 min, t_2 = 29.5 min.

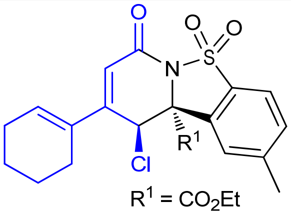

$R^1 = CO_2Et$
3d; 82%; 91:9 er

Ethyl 10-chloro-9-(cyclohex-1-en-1-yl)-2-methyl-7-oxo-10,10a-dihydro-7H-benzo[4,5] isothiazolo[2,3-a]pyridine-10a-carboxylate 5,5-dioxide (3d): Colorless oil. ^1H NMR (400 MHz, $CDCl_3$): δ 7.79 (d, *J* = 8.4 Hz, 1H), 7.50 (d, *J* = 6.4 Hz, 2H), 6.61 (t, *J* = 4.4 Hz, 1H), 6.04 (s, 1H), 5.71 (s, 1H), 4.27–4.11 (m, 2H), 2.54 (s, 3H), 2.35 (d, *J* =4.4 Hz, 2H), 2.21 (d, *J* = 4.8 Hz, 2H),1.79-1.65 (m, 4H),1.18 (t, *J* = 7.2 Hz, 3H); ^{13}C NMR (100 MHz, $CDCl_3$): δ167.7, 159.7, 151.9, 145.7, 135.5, 132.7, 132.6, 131.6, 130.2, 124.0, 121. 9, 117.3, 71.9; 64.2, 52.7, 26.6, 25.4, 22.1, 22.0, 21.3, 13.8; HRMS (ESI) calcd. For $C_{21}H_{22}ClNO_5S$ $[M+H]^+$: 436.0985, Found: 436.0986. $[\alpha]^{20}_D$ = 88.2 (*c* = 5.0 mg/mL, $CHCl_3$). The er value was determined by HPLC (Chiralcel OD, hexane/isopropanol = 80:20, flow rate = 0.75 mL/min), retention time: t_1 = 15.2 min, t_2 = 21.8 min.

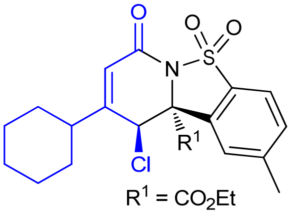

$R^1 = CO_2Et$
3e; 84%; 96:4 er

Ethyl 10-chloro-9-cyclohexyl-2-methyl-7-oxo-10,10a-dihydro-7H-benzo[4,5]isothiazolo

[2,3-a]pyridine-10a-carboxylate 5,5-dioxide (3e): Colorless oil. ^{1}H NMR (400 MHz, $CDCl_3$): δ 7.77 (d, J = 8.4 Hz, 1H), 7.50 (d, J = 7.6 Hz, 2H), 5.96 (d, J = 1.2 Hz, 1H), 5.35 (s, 1H), 4.30–4.09 (m, 2H), 2.53 (s, 3H), 2.30-2.24 (m, 1H), 2.00-1.74 (m, 5H), 1.38-1.31 (m, 3H), 1.28-1.16 (m, 5H); ^{13}C NMR (100 MHz, $CDCl_3$): δ 167.5, 161.1, 159.0, 145.8, 132.6, 132.5, 130.1, 124.1, 121.8, 120.6, 71.8, 64.2, 55.1, 43.0, 32.0, 31.0, 26.3, 25.9, 25.6, 22.0, 13.8; HRMS (ESI) calcd. For $C_{21}H_{24}ClNO_5S$ $[M+H]^+$: 438.1139, Found: 438.1142. $[\alpha]^{20}_D$ = 322.8 (c = 5.0 mg/mL, $CHCl_3$). The er value was determined by HPLC (Chiralcel OD, hexane/isopropanol = 85:15, flow rate = 0.70 mL/min), retention time: t_1 = 16.9 min, t_2 = 19.3 min.

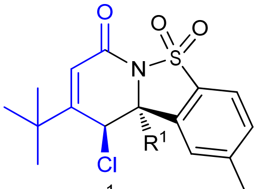

3f; 76%; 94:6 er

Ethyl 9-(tert-butyl)-10-chloro-2-methyl-7-oxo-7,10-dihydro-10aH-benzo[4,5]isothiazolo [2,3-a]pyridine-10a-carboxylate 5,5-dioxide (3f): Colorless oil. ^{1}H NMR (400 MHz, $CDCl_3$): δ 7.77 (d, J = 8.0 Hz, 1H), 7.54 (s, 1H), 7.51 (d, J = 8.0 Hz, 1H), 6.10 (s, 1H), 5.42 (s, 1H), 4.30-4.21 (s, 1H), 4.15-4.06 (s, 1H), 2.54 (s, 3H), 1.25 (s, 9H), 1.20 (t, J = 7.1 Hz, 3H). ^{13}C NMR (100 MHz, $CDCl_3$): δ 167.4, 163.7, 159.2, 145.8, 132.7, 132.6, 130.2, 124.2, 121.8, 121.0, 72.3, 64.2, 53.3, 36.5, 29.0, 22.0, 13.8; IR ν (cm^{-1}) 1754, 1687, 1479, 1351, 1190, 873; HRMS (ESI) calcd. For $C_{19}H_{22}ClNO_5S$ $[M+H]^+$: 412.0985, Found: 412.0988. $[\alpha]^{20}_D$ = 249.8 (c = 5.0 mg/mL, $CHCl_3$). The er value was determined by HPLC (Chiralcel OD, hexane/isopropanol = 80:20, flow rate = 0.75 mL/min), retention time: t_1 = 12.6 min, t_2 = 17.3 min.

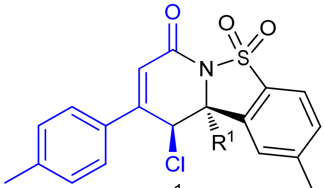

3g; 90%; 91:9 er

Ethyl 10-chloro-2-methyl-7-oxo-9-(p-tolyl)-10,10a-dihydro-7H-benzo[4,5]isothiazolo [2,3-a]pyridine-10a-carboxylate 5,5-dioxide (3g): Colorless oil. ^{1}H NMR (400 MHz, $CDCl_3$): δ 7.81 (d, J = 8.0 Hz, 1H), 7.57-7.52 (m, 4H), 7.30 (d, J = 8.0 Hz, 2H), 6.42 (s, 1H), 5.90 (s, 1H), 4.28-4.10 (m, 2H), 2.55 (s, 3H), 2.34 (s, 3H), 1.17 (t, J = 7.2 Hz, 3H). ^{13}C NMR (100 MHz, $CDCl_3$): δ167.6, 159.1, 152.2, 145.9, 142.2, 132.7, 132.7, 130.8, 130.2, 130.0, 126.7, 124.1, 121.9, 119.9, 72.0, 64.3, 54.9, 22.0, 21.4, 13.8; IR ν (cm^{-1}) 1736, 1690, 1458, 1342, 1018, 841; HRMS (ESI) calcd. For $C_{22}H_{20}ClNO_5S$ $[M+H]^+$: 446.0829, Found: 446.0851. $[\alpha]^{20}_D$ = 272.8 (c = 5.0 mg/mL, $CHCl_3$). The er value was determined by HPLC (Chiralcel OD, hexane/isopropanol = 85:15, flow rate = 0.70 mL/min), retention time: t_1 = 22.7 min, t_2 =

26.0 min.

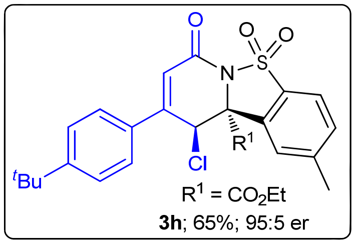

Ethyl 9-(4-(tert-butyl)phenyl)-10-chloro-2-methyl-7-oxo-7,10-dihydro-10aH-benzo[4,5] isothiazolo[2,3-a]pyridine-10a-carboxylate 5,5-dioxide (3h): Colorless oil. ^{1}H NMR (400 MHz, $CDCl_3$): δ 7.80 (d, J = 8.0 Hz, 1H), 7.60-7.56 (m, 3H), 7.55-7.49 (m, 3H), 6.43 (s, 1H), 5.93 (s, 1H), 4.27-4.10 (m, 2H), 2.55 (s, 3H), 1.34 (s, 9H), 1.16 (t, J = 7.2 Hz, 3H). ^{13}C NMR (100 MHz, $CDCl_3$): δ 167.6, 159.2, 155.2, 152.1, 145.9, 132.7, 132.6, 130.6, 130.0, 126.5, 126.4, 124.1, 121.9, 119.9, 72.0, 64.3, 54.8, 35.0, 31.1, 22.0, 13.8; HRMS (ESI) calcd. For $C_{25}H_{26}ClNO_5S$ $[M+H]^+$: 488.1298, Found: 488.1295. $[\alpha]^{20}_{D}$ = 217.6 (c = 5.0 mg/mL, $CHCl_3$). The er value was determined by HPLC (Chiralcel OD, hexane/isopropanol = 80:20, flow rate = 0.75 mL/min), retention time: t_1 = 13.2 min, t_2 = 16.7 min.

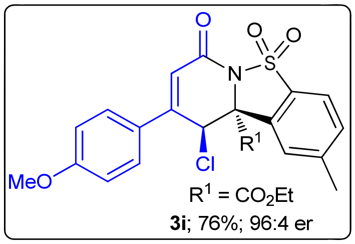

Ethyl 10-chloro-9-(4-methoxyphenyl)-2-methyl-7-oxo-10,10a-dihydro-7H-benzo[4,5] isothiazolo[2,3-a]pyridine-10a-carboxylate 5,5-dioxide (3i): Colorless oil. ^{1}H NMR (400 MHz, $CDCl_3$): δ 7.83 (d, J = 8.8 Hz, 1H), 7.53 (d, J = 8.0 Hz, 2H), 7.30 (d, J = 8.0 Hz, 2H), 7.23- 7.18 (m, 2H), 6.42 (s, 1H), 5.90 (s, 1H), 4.28-4.10 (m, 2H), 3.96 (s, 3H), 2.42 (s, 3H), 1.17 (t, J = 7.2 Hz, 3H). ^{13}C NMR (100 MHz, $CDCl_3$): δ 167.6, 162.3, 159.2, 151.7, 145.8, 132.7, 132.7, 130.0, 128.4, 125.7, 124.0, 122.0, 118.6, 114.9, 71.9, 64.3, 55.5, 54.9, 22.0, 13.8; HRMS (ESI) calcd. For $C_{22}H_{20}ClNO_6S$ $[M+H]^+$: 462.0778, Found: 462.0778. $[\alpha]^{20}_{D}$ = 271.6 (c = 5.0 mg/mL, $CHCl_3$). The er value was determined by HPLC (Chiralcel AS-H, hexane/isopropanol = 80:20, flow rate = 0.75 mL/min), retention time: t_1 = 63.5 min, t_2 = 82.3 min.

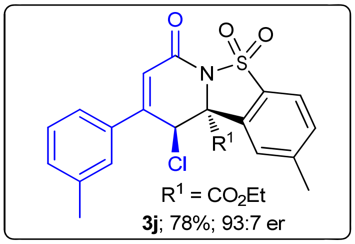

Ethyl 10-chloro-2-methyl-7-oxo-9-(m-tolyl)-7,10-dihydro-10aH-benzo[4,5]isothiazolo [2,3-a]pyridine-10a-carboxylate 5,5-dioxide (3j): Colorless oil. ^{1}H NMR (400 MHz, $CDCl_3$): δ 7.81 (d, *J* = 8.0 Hz, 1H), 7.58 (s, 1H), 7.53 (d, *J* = 8.0 Hz, 1H), 7.44-7.31 (m, 4H), 6.42 (s, 1H), 5.89 (s, 1H), 4.29-4.14 (m, 2H), 2.55 (s, 3H), 2.44 (s, 3H), 1.17 (t, *J* = 7.2 Hz, 3H). ^{13}C NMR (100 MHz, $CDCl_3$): δ 167.6, 159.0, 152.6, 145.9, 139.2, 133.7, 132.8, 132.6, 132.2, 129.9, 129.2, 127.3, 124.1, 123.9, 121.9, 120.8, 72.0, 64.4, 55.0, 22.0, 21.4, 13.8; HRMS (ESI) calcd. For $C_{22}H_{20}ClNO_5S$ $[M+H]^+$: 446.0829, Found: 446.0827. $[\alpha]^{20}_{D}$ = 236.7 (*c* = 5.0 mg/mL, $CHCl_3$). The er value was determined by HPLC (Chiralcel OD, hexane/isopropanol = 80:20, flow rate = 0.75 mL/min), retention time: t_1 = 16.2 min, t_2 = 20.0 min.

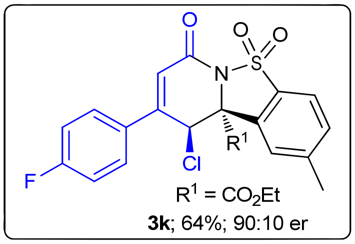

Ethyl 10-chloro-9-(4-fluorophenyl)-2-methyl-7-oxo-7,10-dihydro-10aH-benzo[4,5] isothiazolo[2,3-a]pyridine-10a-carboxylate 5,5-dioxide (3k): Colorless oil. ^{1}H NMR (400 MHz, $CDCl_3$): δ 7.83-7.79 (m, 1H), 7.66-7.60 (m, 2H), 7.56-7.52 (m, 2H), 7.23-7.16 (m, 2H), 6.38 (s, 1H), 5.85 (s, 1H), 4.29-4.13 (m, 2H), 2.55 (s, 3H), 1.17 (t, *J* = 7.2 Hz, 3H). ^{13}C NMR (100 MHz, $CDCl_3$): δ 167.6, 164.6 (*J* = 253.6 Hz), 158.8, 151.4, 146.0, 132.8, 132.6, 130.0 (*J* = 3.3 Hz), 129.8, 129.0 (*J* = 8.7 Hz), 124.0, 122.0, 120.9, 116.7 (*J* = 22.0 Hz). ^{19}F NMR (300 MHz, $CDCl_3$): δ -107.7; IR ν (cm^{-1}) 1756, 1694, 1414, 1346, 1192, 845; HRMS (ESI) calcd. For $C_{21}H_{17}ClFNO_5S$ $[M+H]^+$: 450.0578, Found: 450.0591. $[\alpha]^{20}_{D}$ = 242.0 (*c* = 5.0 mg/mL, $CHCl_3$). The er value was determined by HPLC (Chiralcel OD, hexane/isopropanol = 80:20, flow rate = 0.75 mL/min), retention time: t_1 = 18.9 min, t_2 = 24.0 min.

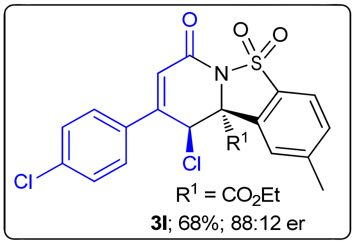

Ethyl 10-chloro-9-(4-chlorophenyl)-2-methyl-7-oxo-10,10a-dihydro-7H-benzo[4,5] isothiazolo[2,3-a]pyridine-10a-carboxylate 5,5-dioxide (3l): Colorless oil. ^{1}H NMR (400 MHz, $CDCl_3$): δ 7.82 (d, *J* = 8.8 Hz, 1H), 7.58-7.47 (m, 6H), 6.42 (s, 1H), 5.85 (s, 1H), 4.29–4.13 (m, 2H), 2.56 (s, 3H), 1.17 (t, *J* = 7.2 Hz, 3H); ^{13}C NMR (100 MHz, $CDCl_3$): δ 167.6, 158.7, 151.3, 146.1, 137.8, 132.9, 132.6, 132.2, 129.7, 128.1, 124.0, 122.0, 121.3, 71.9, 64.5, 54.9, 22.0, 13.8; HRMS (ESI) calcd. For $C_{21}H_{17}C_{12}NO_5S$ $[M+H]^+$: 466.0283, Found: 466.0281. $[\alpha]^{20}_{D}$ = 171.8 (*c* = 5.0 mg/mL, $CHCl_3$). The er value was determined by HPLC (Chiralcel AS-H, hexane/isopropanol = 85:15, flow rate = 0.75 mL/min), retention time: t_1 =

84.4 min, t_2 = 98.5 min.

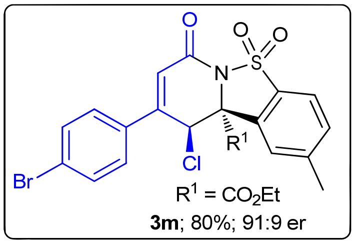

Ethyl 9-(4-bromophenyl)-10-chloro-2-methyl-7-oxo-10,10a-dihydro-7H-benzo[4,5] isothiazolo[2,3-a]pyridine-10a-carboxylate 5,5-dioxide (3m): Colorless solid, m.p. 238-240. ^{1}H NMR (400 MHz, $CDCl_3$): δ 7.20 (d, *J* = 8.4 Hz, 1H), 7.66-7.48 (m, 6H), 6.43 (s, 1H), 5.84 (s, 1H), 4.27-4.10 (m, 2H), 2.56 (s, 3H), 1.17 (t, *J* = 7.2 Hz, 3H); ^{13}C NMR (100 MHz, $CDCl_3$): δ 167.5, 158.7, 151.3, 146.0, 132.9, 132.7, 132.6, 132.5, 129.7, 128.2, 126.1, 124.0, 122.0, 121.3, 71.9, 64.5, 54.8, 22.1, 13.8; HRMS (ESI) calcd. For $C_{21}H_{17}BrClNO_5S$ $[M+H]^+$: 511.9757, Found: 511.9776. $[\alpha]^{20}_D$ = 208.8 (*c* = 5.0 mg/mL, $CHCl_3$). The er value was determined by HPLC (Chiralcel AS-H, hexane/isopropanol = 80:20, flow rate = 0.60 mL/min), retention time: t_1 = 72.4 min, t_2 = 86.0 min.

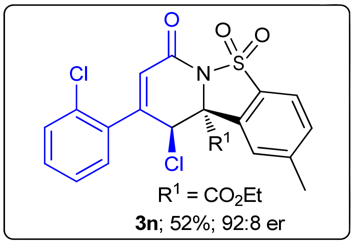

Ethyl 10-chloro-9-(2-chlorophenyl)-2-methyl-7-oxo-10,10a-dihydro-7H-benzo[4,5] isothiazolo[2,3-a]pyridine-10a-carboxylate 5,5-dioxide (3n): Colorless oil. ^{1}H NMR (400 MHz, $CDCl_3$): δ 7.82 (d, *J* = 8.0 Hz, 1H), 7.59-7.52 (m, 3H), 7.47-7.38 (m, 3H), 6.34 (s, 1H), 5.76 (s, 1H), 4.40-4.20 (m, 2H), 2.56 (s, 3H), 1.29 (t, *J* = 7.2 Hz, 3H); ^{13}C NMR (100 MHz, $CDCl_3$): δ167.2, 158.5, 150.1, 145.8, 133.8, 132.7, 132.2, 132.2, 131.3, 130.6, 130.4, 130.2, 127.3, 126.8, 124.8, 121.7, 71.7, 64.6, 56.7, 22.0, 13.8; HRMS (ESI) calcd. For $C_{21}H_{17}C_{l2}NO_5S$ $[M+H]^+$: 466.0283, Found: 466.0282. $[\alpha]^{20}_D$ = 106.8 (*c* = 5.0 mg/mL, $CHCl_3$). The er value was determined by HPLC (Chiralcel OD, hexane/isopropanol = 80:20, flow rate = 0.75 mL/min), retention time: t_1 = 17.5 min, t_2 = 32.9 min.

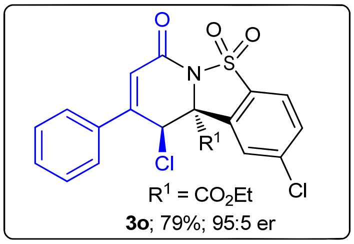

Ethyl 10-chloro-7-oxo-9-(p-tolyl)-7,10-dihydro-10aH-benzo[4,5]isothiazolo[2,3-a]

pyridine-10a-carboxylate 5,5-dioxide (3o): Colorless oil. ^{1}H NMR (400 MHz, $CDCl_3$): δ 7.94 (d, *J* = 7.8 Hz, 1H), 7.85-7.78 (m, 2H), 7.78-7.72 (m, 1H), 7.52 (d, *J* = 8.2 Hz, 2H), 7.31 (d, *J* = 8.0 Hz, 2H), 6.43 (s, 1H), 5.92 (m, 1H), 4.26-4.13 (m, 2H), 2.42 (s, 3H), 1.16 (t, *J* = 7.1 Hz, 3H). ^{13}C NMR (100 MHz, $CDCl_3$): δ 167.4, 159.1, 152.2, 142.2, 135.3, 134.4, 131.8, 130.7, 130.1, 129.8, 126.7, 124.1, 122.2, 119.9, 72.1, 64.4, 54.8, 21.4, 13.8; HRMS (ESI) calcd. For $C_{21}H_{20}ClNO_5S$ $[M+H]^+$: 452.0829, Found: 452.0816. $[\alpha]^{20}_D$ = 173.5 (*c* = 5.0 mg/mL, $CHCl_3$). The er value was determined by HPLC (Chiralcel OD, hexane/isopropanol = 80:20, flow rate = 0.75 mL/min), retention time: t_1 = 15.2 min, t_2 = 18.8 min.

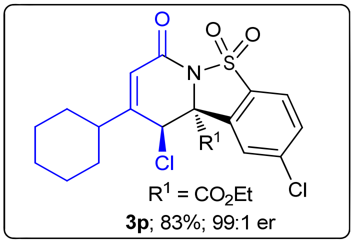

Ethyl 2,10-dichloro-9-cyclohexyl-7-oxo-10,10a-dihydro-7H-benzo[4,5]isothiazolo[2,3-a] pyridine-10a-carboxylate 5,5-dioxide (3p): Colorless oil. ^{1}H NMR (400 MHz, $CDCl_3$): δ 7.84 (d, *J* = 8.4 Hz, 1H), 7.12-7.67 (m, 2H), 5.99 (s, 1H), 5.33 (s, 1H),, 4.32-4.13 (m, 2H), 2.32-2.24 (m, 1H), 1.99-1.75 (m, 5H), 1.39-1.21 (m, 8H); ^{13}C NMR (100 MHz, $CDCl_3$): δ 166.9, 161.0, 158.8, 141.0, 133.6, 132.4, 131.8, 124.4, 123.3, 120.6, 71.6, 64.6, 43.0, 32.0, 31.0, 26.3, 54.9, 25.9, 25.6, 13.8; HRMS (ESI) calcd. For $C_{20}H_{21}Cl_2NO_5S$ $[M+H]^+$: 456.0439, Found: 456.0424. $[\alpha]^{20}_D$ = 180.4 (*c* = 5.0 mg/mL, $CHCl_3$). The er value was determined by HPLC (Chiralcel OD, hexane/isopropanol = 85:15, flow rate = 0.75 mL/min), retention time: t_1 = 15.6 min, t_2 = 17.7 min.

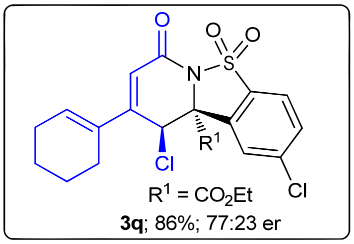

Ethyl 2,10-dichloro-9-(cyclohex-1-en-1-yl)-7-oxo-10,10a-dihydro-7H-benzo[4,5]isothiazolo[2,3-a]pyridine-10a-carboxylate 5,5-dioxide (3q): ^{1}H NMR (400 MHz, $CDCl_3$): δ 7.85 (d, *J* = 10.8 Hz, 1H), 7.74–7.68 (m, 2H), 6.62 (s, 1H), 6.04 (s, 1H), 5.69 (s, 1H), 4.31-4.17 (m, 2H), 2.35-2.20 (m, 4H), 1.80-1.60 (m, 4H), 1.88 (t, *J* = 9.2 Hz, 3H); ^{13}C NMR (100 MHz, $CDCl_3$): δ 166.9, 159.5, 151.7, 141.0, 135.9, 133.8, 132.3, 131.9, 131.5, 124.4, 123.4, 117.1, 71.7, 64.5, 52.5, 26.6, 25.4, 22.0, 21.3, 13.7; HRMS (ESI) calcd. For $C_{20}H_{19}C_{l2}NO_5S$ $[M+H]^+$: 456.0439, Found: 456.0424. $[\alpha]^{20}_D$ = 187.6 (*c* = 5.0 mg/mL, $CHCl_3$). The er value was determined by HPLC (Chiralcel OD, hexane/isopropanol = 85:15, flow rate = 0.75 mL/min), retention time: t_1 = 17.2 min, t_2 = 24.2 min.

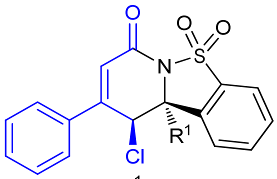

$R^1 = CO_2Et$

3r; 82%; 83:17 er

Ethyl 10-chloro-7-oxo-9-phenyl-7,10-dihydro-10aH-benzo[4,5]isothiazolo[2,3-a]pyridine-10a-carboxylate 5,5-dioxide (3r): Colorless oil. ^{1}H NMR (400 MHz, $CDCl_3$): δ 7.95 (d, J = 7.8 Hz, 1H), 7.85-7.73 (m, 3H), 7.65-7.60 (m, 2H), 7.55-7.47 (m, 3H), 6.45 (s, 1H), 5.92 (s, 1H), 4.29-4.13 (m, 2H), 1.17 (t, J = 7.1 Hz, 3H). ^{13}C NMR (100 MHz, $CDCl_3$): δ 167.4, 158.9, 152.4, 135.3, 134.5, 133.7, 131.8, 131.4, 129.7, 129.4, 126.8, 124.1, 122.2, 121.0, 72.1, 64.5, 55.0, 13.8; HRMS (ESI) calcd. For $C_{20}H_{16}ClNO_5S$ $[M+H]^+$: 418.0510, Found: 458.0512. $[\alpha]^{20}_D$ = 184.0 (c = 5.0 mg/mL, $CHCl_3$). The er value was determined by HPLC (Chiralcel AS-H, hexane/isopropanol = 80:20, flow rate = 0.75 mL/min), retention time: t_1 = 46.7 min, t_2 = 58.3 min.

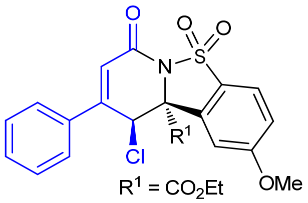

$R^1 = CO_2Et$

3s; 85%; 85:15 er

Ethyl 10-chloro-2-methoxy-7-oxo-9-phenyl-7,10-dihydro-10aH-benzo[4,5]isothiazolo[2,3-a]pyridine-10a-carboxylate 5,5-dioxide. ^{1}H NMR (400 MHz, $CDCl_3$): δ 7.83 (d, J = 8.7 Hz, 1H), 7.65-7.60 (m, 2H), 7.54-7.47 (m, 3H), 7.21 (dd, J = 8.7, 2.2 Hz, 1H), 7.17 (d, J = 2.2 Hz, 1H), 6.43 (s, 1H), 5.87 (s, 1H), 4.30-4.15 (m, 2H), 3.96 (s, 3H), 1.18 (t, J = 7.1 Hz, 3H). ^{13}C NMR (100 MHz, $CDCl_3$): δ 167.5, 164.5, 158.9, 152.3, 133.8, 132.0, 131.4, 129.4, 127.1, 126.8, 123.7, 121.0, 118.4, 108.2, 71.9, 64.4, 56.2, 55.0, 13.8. HRMS (ESI) calcd. For $C_{20}H_{16}ClNO_5S$ $[M+H]^+$: 448.0622, Found: 448.0629. $[\alpha]^{20}_D$ = 187.0 (c = 5.0 mg/mL, $CHCl_3$). The er value was determined by HPLC (Chiralcel OD, hexane/isopropanol = 80:20, flow rate = 0.75 mL/min), retention time: t_1 = 22.9 min, t_2 = 27.8 min.

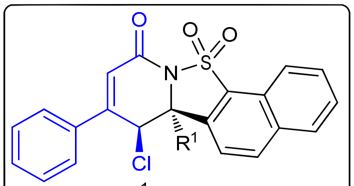

$R^1 = CO_2Et$

3t; 79%; 82:18 er

Ethyl 7-chloro-10-oxo-8-phenyl-7,10-dihydro-6bH-naphtho[2',1':4,5]isothiazolo[2,3-a]pyridine

-6b-carboxylate 12,12-dioxide. ^{1}H NMR (400 MHz, $CDCl_3$): δ 8.48 (d, J = 8.0 Hz, 1H), 8.25 (d, J = 8.6 Hz, 1H), 8.04 (d, J = 8.1 Hz, 1H), 7.83-7.71 (m, 3H), 7.68-7.63 (m, 2H), 7.55-7.48 (m, 3H), 6.48 (s, 1H), 6.03 (s, 1H), 4.29-4.12 (m, 2H), 1.16 (t, J = 7.1 Hz, 3H). ^{13}C NMR (100 MHz, $CDCl_3$): δ 167.3, 158.8, 152.5, 135.6, 134.3, 133.8, 131.4, 131.1, 131.0, 129.4, 129.1, 128.9, 128.5, 126.8, 125.1, 123.2, 121.1, 119.7, 72.1, 64.5, 54.7, 12.8. HRMS (ESI) calcd. For $C_{20}H_{16}ClNO_5S$ $[M+H]^+$: 468.9294, Found: 468.9298. $[\alpha]^{20}_D$ = 189.0 (c = 5.0 mg/mL, $CHCl_3$). The er value was determined by HPLC (Chiralcel OD-H, hexane/isopropanol = 80:20, flow rate = 0.75 mL/min), retention time: t_1 = 28.4 min, t_2 = 35.8 min.

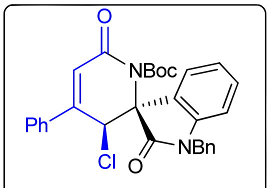

5a; 75%; 6:1 dr; 99:1 er

***tert*-butyl 1-benzyl-3'-chloro-2,6'-dioxo-4'-phenyl-3',6'-dihydro-1'H-spiro[indoline-3,2'-pyridine]-1'-carboxylate (5a):** Colorless solid, m.p. 180-182. ^{1}H NMR (400 MHz, $CDCl_3$): δ 7.64 (d, J = 10.0 Hz, 1H), 7.50-7.44 (m, 2H), 7.42-7.38 (m, 2H), 7.31-7.29 (m, 6H), 7.09 (t, J = 10.0 Hz, 1H), 6.85 (d, J = 10.0 Hz, 1H), 6.35 (s, 1H), 5.10 (d, J = 20.8 Hz, 1H), 4.85 (s, 1H), 4.12 (d, J = 20.8 Hz, 1H), 1.20 (s, 9H); ^{13}C NMR (100 MHz, $CDCl_3$): δ 172.7, 163.0, 150.3, 147.2, 142.5, 135.4, 134.5, 130.4, 130.3, 129.0, 128.9, 128.0, 127.6, 126.8, 126.4, 126.0, 122.5, 121.3, 109.5, 84.7, 67.5, 57.1, 44.4, 27.3; HRMS (ESI) calcd. For $C_{30}H_{27}ClN_2O_4$ $[M+H]^+$: 515.1738, Found: 515.1735. $[\alpha]^{20}_D$ = –78.6 (c = 5.0 mg/mL, $CHCl_3$). The er value was determined by HPLC (Chiralcel OD, hexane/isopropanol = 80:20, flow rate = 0.75 mL/min), retention time: t_1 = 11.0 min, t_2 = 16.0 min.

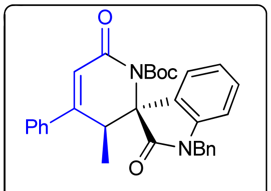

5b; 71%; 7:1 dr: >99:1 er

***tert*-butyl 1-benzyl-3'-methyl-2,6'-dioxo-4'-phenyl-3',6'-dihydro-1'H-spiro[indoline-3,2'-pyridine]-1'-carboxylate (5b):** Colorless oil. ^{1}H NMR (400 MHz, $CDCl_3$): δ 7.34-7.25 (m, 12H), 7.06 (t, J = 12.8 Hz, 1H), 6.85 (d, J = 10.4 Hz, 1H), 6.33 (s, 1H), 5.15 (d, J = 20.4 Hz, 1H), 4.61 (d, J = 20.4 Hz, 1H), 3.19–3.12 (dd, J_1 = 9.2 Hz, J_2 = 18.4 Hz, 1H), 1.13 (s, 9H), 1.11 (d, J = 9.2 Hz, 1H); ^{13}C NMR (100 MHz, $CDCl_3$): δ175.7, 164.0, 154.4, 151.3, 142.6, 136.7, 135.9, 129.6, 129.6, 128.8, 128.8, 127.8, 127.7, 127.4, 126.4, 124.7, 122.3, 119.3, 109.4, 84.1, 67.6, 44.1, 39.0, 27.5, 16.1; HRMS (ESI) calcd. For $C_{31}H_{30}N_2O_4$ $[M+H]^+$: 495.2284, Found: 495.2294. $[\alpha]^{20}_D$ = –87.0 (c = 5.0 mg/mL, $CHCl_3$). The er value was determined by HPLC (Chiralcel IA, hexane/isopropanol = 80:20, flow rate = 0.75 mL/min), retention time: t_1 = 17.5 min, t_2 = 52.8 min.

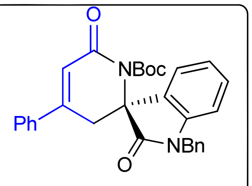

5c; 81%; 93:7 er

***tert*-butyl 1-benzyl-2,6'-dioxo-4'-phenyl-3',6'-dihydro-1'H-spiro[indoline-3,2'-pyridine]-1'-carboxylate (5c):** Colorless oil. ^{1}H NMR (400 MHz, $CDCl_3$): δ 7.41-7.17 (m, 12H), 6.99-6.92 (m, 1H),6.78-6.75 (d, *J* = 10.4 Hz, 1H),6.53 (s, 1H), 5.06 (d, *J* = 21.6 Hz, 1H), 4.87 (d, *J* = 21.6 Hz, 1H), 3.51 (d, *J* = 22.8 Hz, 1H), 2.80 (d, *J* = 21.6 Hz, 1H), 1.40 (s, 9H); ^{13}C NMR (100 MHz, $CDCl_3$): δ 175.2, 163.5, 151.7, 148.6, 141.4, 136.5, 135.5, 130.3, 130.3, 129.4, 128.9, 128.8, 127.7, 127.4, 125.9, 122.9, 122.4, 119.7, 109.7, 84.2, 64.7, 44.3, 37.6, 29.7, 27.8; HRMS (ESI) calcd. For $C_{30}H_{28}N_2O_4$ $[M+H]^+$: 481. 2127, Found: 481.2118. $[\alpha]^{20}_{D}$ = –88.6 (*c* = 5.0 mg/mL, $CHCl_3$). The er value was determined by HPLC (Chiralcel OD, hexane/isopropanol = 80:20, flow rate = 0.75 mL/min), retention time: t_1 = 11.9 min, t_2 = 16.8 min.

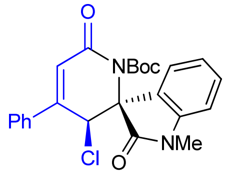

5d; 65%; 7:1 dr; 98:2 er

***tert*-butyl 3'-chloro-1-methyl-2,6'-dioxo-4'-phenyl-3',6'-dihydro-1'H-spiro[indoline-3,2'-pyridine]-1'-carboxylate (5d):** Colorless oil, 65%; 7:1 dr; 98:2 er. ^{1}H NMR (400 MHz, $CDCl_3$): δ 7.70-7.68 (d, *J* = 7.6 Hz, 1H), 7.56-7.54 (m, 2H), 7.46-7.41 (m, 4H), 7.14 (t, *J* = 7.6 Hz, 1H), 6.90 (d, *J* = 7.6 Hz, 1H), 4.83 (s, 1H), 3.20 (s, 3H), 1.15 (s, 9H); ^{13}C NMR (100 MHz, $CDCl_3$): δ 172.8, 163.0, 150.0, 147.0, 143.5, 134.5, 130.6, 130.4, 129.0, 127.1, 126.4, 126.0, 122.6, 121.0, 108.7, 84.5, 67.4, 56.7, 27.2, 26.8; HRMS (ESI) calcd. For $C_{24}H_{23}ClN_2O_4$ $[M+H]^+$: 439.1345, Found: 439.1340. $[\alpha]^{20}_{D}$ = –30.0 (*c* = 5.0 mg/mL, $CHCl_3$). The er value was determined by HPLC (Chiralcel OD, hexane/isopropanol = 80:20, flow rate = 0.75 mL/min), retention time: t_1 = 8.8 min, t_2 = 113.9 min.

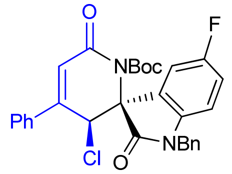

5e; 83%; 20:1 dr; 99:1 er

***tert*-Butyl 1-benzyl-3'-chloro-5-fluoro-2,6'-dioxo-4'-phenyl-3',6'-dihydro-1'H-spiro[indoline-3,2'-pyridine]-1'-carboxylate (5e):** Colorless oil. ^{1}H NMR (400 MHz, $CDCl_3$): δ 7.51-7.47 (m, 2H), 7.46-7.41 (m, 3H), 7.39 (dd, *J* = 8.0, 2.5 Hz, 1H), 7.33-7.28 (m, 5H), 7.01 (td, *J* = 8.8, 2.6 Hz, 1H), 6.74 (td, *J* = 8.6, 4.1 Hz, 1H), 6.54 (s, 1H), 5.05 (d, *J* = 15.6 Hz, 1H), 4.90 (s, 1H), 4.68 (d, *J* = 15.6 Hz, 1H), 1.29 (s, 9H). ^{13}C NMR (100 MHz, $CDCl_3$): δ 172.5,

162.6, 158.7 (J = 242.6 Hz), 150.7, 147.3, 138.4, 135.0, 134.3, 130.5, 129.0, 128.9, 128.0, 127.6 (J = 8.4 Hz), 125.5, 126.4, 121.3, 116.6 (J = 23.3 Hz), 114.7 (J = 26.1 Hz), 110.2 (J = 8.1 Hz), 85.0, 67.6, 56.9, 44.6, 27.4. ^{19}F NMR (300 MHz, $CDCl_3$): δ -119.2; HRMS (ESI) calcd. For $C_{30}H_{26}ClFN_2O_4$ $[M+H]^+$: 533.1655, Found: 533.1659. $[\alpha]^{20}_D$ = –89.0 (c = 5.0 mg/mL, $CHCl_3$). The er value was determined by HPLC (Chiralcel IA, hexane/isopropanol = 80:20, flow rate = 0.75 mL/min), retention time: t_1 = 16.9 min, t_2 = 61.0 min.

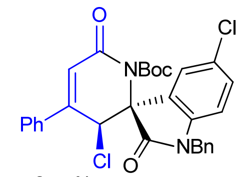

5f; 73%; 7:1; dr; 95:5 er

***tert*-Butyl 1-benzyl-3',5-dichloro-2,6'-dioxo-4'-phenyl-3',6'-dihydro-1'H-spiro[indoline -3,2'-pyridine]-1'-carboxylate (5f):** Colorless oil. ^{1}H NMR (400 MHz, $CDCl_3$): δ 7.60 (d, J = 2.1 Hz, 1H), 7.50-7.47 (m, 2H), 7.45-7.39 (m, 3H), 7.30-7.26 (m, 6H), 6.74 (d, J = 8.4 Hz, 1H), 6.53 (s, 1H), 5.03 (d, J = 15.6 Hz, 1H), 4.90 (s, 1H), 4.70 (d, J = 15.6 Hz, 1H), 1.29 (s, 9H). ^{13}C NMR (100 MHz, $CDCl_3$): δ 172.4, 162.6, 150.7, 147.3, 141.0, 134.8, 134.3, 130.5, 130.1, 129.0, 128.9, 128.1, 128.1, 127.6, 127.5, 126.7, 126.4, 121.3, 110.6, 85.1, 67.5, 56.8, 44.5, 27.4; HRMS (ESI) calcd. For $C_{30}H_{26}Cl_2N_2O_4$ $[M+H]^+$: 594.1348, Found: 594.1365. $[\alpha]^{20}_D$ = –99.6 (c = 5.0 mg/mL, $CHCl_3$). The er value was determined by HPLC (Chiralcel IA, hexane/isopropanol = 80:20, flow rate = 0.75 mL/min), retention time: t_1 = 15.0 min, t_2 = 57.8 min.

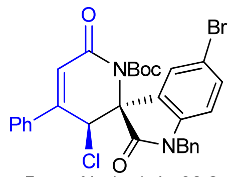

5g; 77%; 17:1 dr; 98:2 er

***tert*-Butyl 1-benzyl-5-bromo-3'-chloro-2,6'-dioxo-4'-phenyl-3',6'-dihydro-1'H -spiro[indoline-3,2'-pyridine]-1'-carboxylate (5g):** Colorless solid, m.p. 191-193. ^{1}H NMR (400 MHz, $CDCl_3$): δ 7.73 (d, J = 2.0 Hz, 1H), 7.50-7.47 (m, 2H), 7.45-7.41 (m, 4H), 7.32-7.29 (m, 5H), 6.69 (d, J = 8.4 Hz, 1H), 6.53 (s, 1H), 5.02 (d, J = 15.6 Hz, 1H), 4.89 (s, 1H), 6.70 (d, J = 15.6 Hz, 1H), 1.29 (s, 9H). ^{13}C NMR (100 MHz, $CDCl_3$): δ 172.3, 162.6, 150.7, 147.3, 141.5, 134.8, 134.3, 133.0, 130.5, 129.4, 129.0, 128.9, 128.0, 127.9, 127.5, 126.4, 121.3, 115.2, 111.0, 85.1, 67.5, 56.8, 44.5, 27.4; HRMS (ESI) calcd. For $C_{30}H_{26}BrClN_2O_4$ $[M+H]^+$: 595.0837, Found: 595.0822. $[\alpha]^{20}_D$ = –49.6 (c = 5.0 mg/mL, $CHCl_3$). The er value was determined by HPLC (Chiralcel IA, hexane/isopropanol = 80:20, flow rate = 0.75 mL/min), retention time: t_1 = 15.7 min, t_2 = 60.0 min.

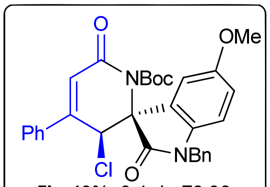

5h, 40%; 3:1 dr, 70:30 er

***tert*-Butyl 1-benzyl-3'-chloro-4'-(4-methoxyphenyl)-2,6'-dioxo-3',6'-dihydro-1'H-spiro[indoline-3,2'-pyridine]-1'-carboxylate (5h).** ^{1}H NMR (400 MHz, $CDCl_3$): δ 7.64 (dd, *J* = 7.6, 0.7 Hz, 1H), 7.48-4.43 (m, 2H), 7.34-7.27 (m, 6H), 7.09 (td, *J* = 7.6, 0.6 Hz, 1H), 6.95-6.90 (m, 2H), 6.84 (d, *J* = 7.8 Hz, 1H), 6.50 (s, 1H), 5.11 (d, *J* = 15.5 Hz, 1H), 4.82 (s, 1H), 4.61 (d, *J* = 15.5 Hz, 1H), 3.83 (s, 3H), 1.19 (s, 9H). ^{13}C NMR (100 MHz, $CDCl_3$): δ 172.8, 163.2, 161.5, 150.4, 146.5, 142.4, 135.4, 130.3, 128.9, 127.9, 127.9, 127.6, 126.9, 126.5, 126.2, 122.4, 119.1, 114.5, 109.5, 84.5, 67.4, 56.9, 55.4, 44.4, 27.3. HRMS (ESI) calcd. For $C_{30}H_{26}BrClN_2O_4$ $[M+H]^+$: 545.1843, Found: 545.1840. $[\alpha]^{20}_{D}$ = –75.6 (*c* = 5.0 mg/mL, $CHCl_3$). The er value was determined by HPLC (Chiralcel IA, hexane/isopropanol = 80:20, flow rate = 0.75 mL/min), retention time: t_1 = 19.3 min, t_2 = 96.5 min.

ORTEPS drawing of 3b, 3m, 5f and 5g from X-ray crystallographic analysis

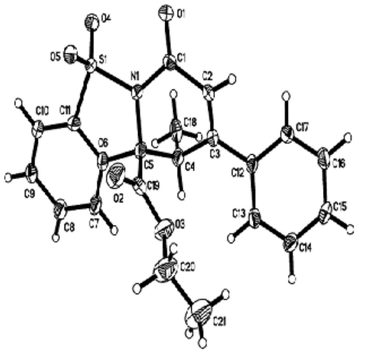

Supplementary Dataset 1
3b CCDC 988901

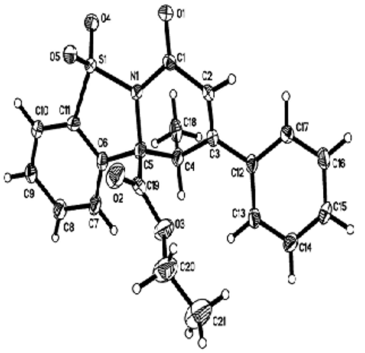

Supplementary Dataset 2
3m CCDC 988902

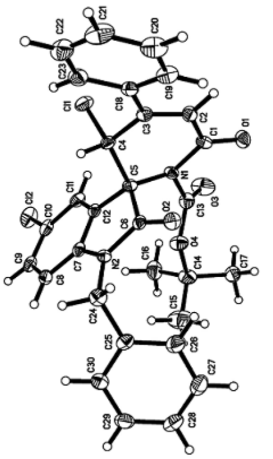

Supplementary Dataset 3
5f CCDC 1011138

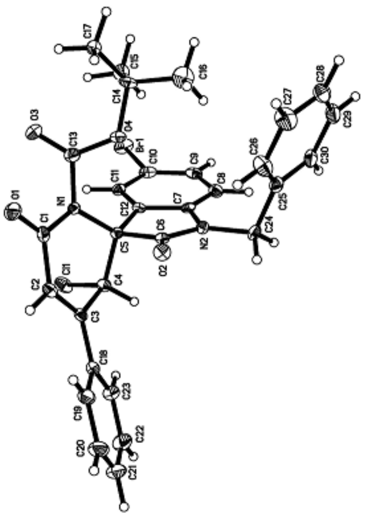

Supplementary Dataset 4
5g CCDC 1011137

Supplementary References

1. Wang, H., Jiang, T. & Xu, M.-H. Simple Branched Sulfur–Olefins as Chiral Ligands for Rh-Catalyzed Asymmetric Arylation of Cyclic Ketimines: Highly Enantioselective Construction of Tetrasubstituted Carbon Stereocenters. *J. Am. Chem. Soc.*, **135**, 971, (2013).
2. Yan, W. Wang, D., Feng, J., Li, P., Zhao, D. & R. Wang. Synthesis of N-Alkoxycarbonyl Ketimines Derived from Isatins and Their Application in Enantioselective Synthesis of 3-Aminooxindoles. *Org. Lett*. **14**, 2512, (2012).
3. Sugimoto, K., Hayashi, R., Nemoto, H., Toyooka, N. & Matsuya, Y. Efficient Approach to 1,2-Diazepines via Formal Diazomethylene Insertion into the C–C bond of Cyclobutenones. *Org. Lett.* **14**, 3510, (2012).
